# Dynamic effects of psychiatric vulnerability, loneliness and isolation on distress during the first year of the COVID-19 pandemic

**DOI:** 10.1038/s44220-024-00371-6

**Published:** 2025-01-09

**Authors:** Lauren Y. Atlas, Cristan Farmer, Jacob S. Shaw, Alison Gibbons, Emily P. Guinee, Juan Antonio Lossio-Ventura, Elizabeth D. Ballard, Monique Ernst, Shruti Japee, Francisco Pereira, Joyce Y. Chung

**Affiliations:** 1 National Center for Complementary and Integrative Health, National Institutes of Health, Bethesda, MD, USA.; 2 National Institute of Mental Health, National Institutes of Health, Bethesda, MD, USA.; 3 National Institute on Drug Abuse, National Institutes of Health, Baltimore, MD, USA.; 4 Office of the Clinical Director, National Institute of Mental Health, National Institutes of Health, Bethesda, MD, USA.; 5 Machine Learning Core, National Institute of Mental Health, National Institutes of Health, Bethesda, MD, USA.; 6 Experimental Therapeutics and Pathophysiology Branch, National Institute of Mental Health, National Institutes of Health, Bethesda, MD, USA.; 7 Branch of Child and Adolescent Psychiatry, National Institute of Mental Health, National Institutes of Health, Bethesda, MD, USA.; 8 Laboratory of Brain and Cognition, National Institute of Mental Health, National Institutes of Health, Bethesda, MD, USA.; 9 National Institutes of Health Clinical Center, Bethesda, MD, USA.

## Abstract

The COVID-19 pandemic’s impact on mental health is challenging to quantify because pre-existing risk, disease burden and public policy varied across individuals, time and regions. Longitudinal, within-person analyses can determine whether pandemic-related changes in social isolation impacted mental health. We analyzed time-varying associations between psychiatric vulnerability, loneliness, psychological distress and social distancing in a US-based study during the first year of the pandemic. We surveyed 3,655 participants about psychological health and COVID-19-related circumstances every 2 weeks for 6 months. We combined self-reports with regional social distancing estimates and a classifier that predicted probability of psychiatric diagnosis at enrollment. Loneliness and psychiatric vulnerability both impacted psychological distress. Loneliness and distress were also linked to social isolation and stress associated with distancing, and psychiatric vulnerability shaped how regional distancing affected loneliness across time. Public health policies should address loneliness when encouraging social distancing, particularly in those at risk for psychiatric conditions.

The COVID-19 pandemic has had well-documented impacts throughout society, including on mental health. While some epidemiological studies indicate that mental health problems increased in response to the pandemic^1^ and remained elevated through the summer of 2020,^2,3^ other studies suggest psychiatric symptoms were not elevated relative to pre-pandemic levels.^4,5^ Many studies indicate responses were heterogeneous across participants^6–9^ and that mental health differed as a function of both sociodemographic and clinical factors. Two meta-analyses of cohort studies that evaluated mental health both before and during the pandemic indicated that studies that measured outcomes in the spring of 2020 observed increases in symptoms, whereas symptoms returned to pre-pandemic levels in studies that measured outcomes in May–July 2020, particularly for studies that measured anxiety, depression or general mental health.^10,11^ However, other meta-analyses indicate that mental health deterioration was present throughout the first year of the pandemic.^12^ Results within each meta-analysis varied widely, pointing to study-specific factors (for example, regional variations in public health policies). Thus, although relationships are well documented through epidemiological and cross-sectional data, much remains unknown in terms of the pandemic’s potentially time-dependent impact on mental health. To address this gap, we need longitudinal within-person analyses that measure mental health at regular intervals over time while considering changes in pandemic-related factors.

Loneliness is a risk factor for physical^13^ and mental health^14^ that was of particular concern during the pandemic in light of public health policies on ‘social distancing,’ which may have increased social isolation. Findings are mixed regarding the impact of social distancing on loneliness and relationships between loneliness, distancing and mental health during the pandemic. One population-based UK study^15^ found that individuals who reported often being lonely between April and July 2020 were 16 times more likely to report a common mental disorder than those who reported hardly being lonely. Yet other studies indicated that population-based levels of loneliness did not change during the pandemic,^8^ and a January 2021 meta-analysis reported that lockdown measures increased anxiety and depression but not loneliness.^16^ The impact of the pandemic on loneliness and mental health might have also differed as a function of psychiatric vulnerability. One longitudinal study from Denmark^9^ found that individuals with previous mental illness reported elevated loneliness that was stable over time, whereas loneliness fluctuated across time in individuals without mental illness, presumably in relation to societal factors such as mandatory social distancing. Importantly, social distancing varied across regions^17^ and based on individuals’ preferences and/or circumstances (for example, essential worker status). Thus, it remains unclear how loneliness affected mental health during the pandemic, whether loneliness and mental health were impacted by social distancing and whether these relationships varied as a function of psychiatric vulnerability. Our goal was to examine dynamic relationships between these variables and to consider relationships with both objective and self-reported measures of distancing and social isolation.

To address these questions, we conducted an internet-based longitudinal study during the first year of the pandemic (Fig. 1). Over 3,600 participants enrolled, more than half of whom reported previous psychiatric treatment.^18^ Participants reported on current mental health, physical health and COVID-19-related circumstances every 2 weeks for 6 months. As the study was led by researchers at the National Institutes of Health (NIH), most participants were based in the United States, where social distancing policies varied widely across regions and individuals. We thus incorporated both self-reported and community-based estimates of distancing to capture relationships between social distancing, loneliness and mental health. We focused on relationships both across individuals and within individuals over time to capture fluctuations in both the pandemic’s impact and individuals’ behaviors and psychological distress. Importantly, a subset of participants had undergone clinical assessments and psychiatric diagnostic interviews at NIH before the pandemic, which allowed us to train a classifier to predict each participant’s likelihood of having a psychiatric diagnosis on enrollment^18^ (that is, psychiatric vulnerability). We combined this classifier with biweekly survey data to evaluate (1) the trajectory of mental health during the first year of the pandemic, (2) whether mental health varied as a function of psychiatric vulnerability, loneliness or their interaction and (3) whether social distancing impacted loneliness and mental health. Our overall goal was to evaluate the joint contribution of psychiatric vulnerability, loneliness and social isolation to mental health over time and determine which factors had the strongest impact on mental health during the first year of the COVID-19 pandemic.

## Results

### Psychiatric vulnerability varies on the basis of demographics

**Fig. 1 |:**
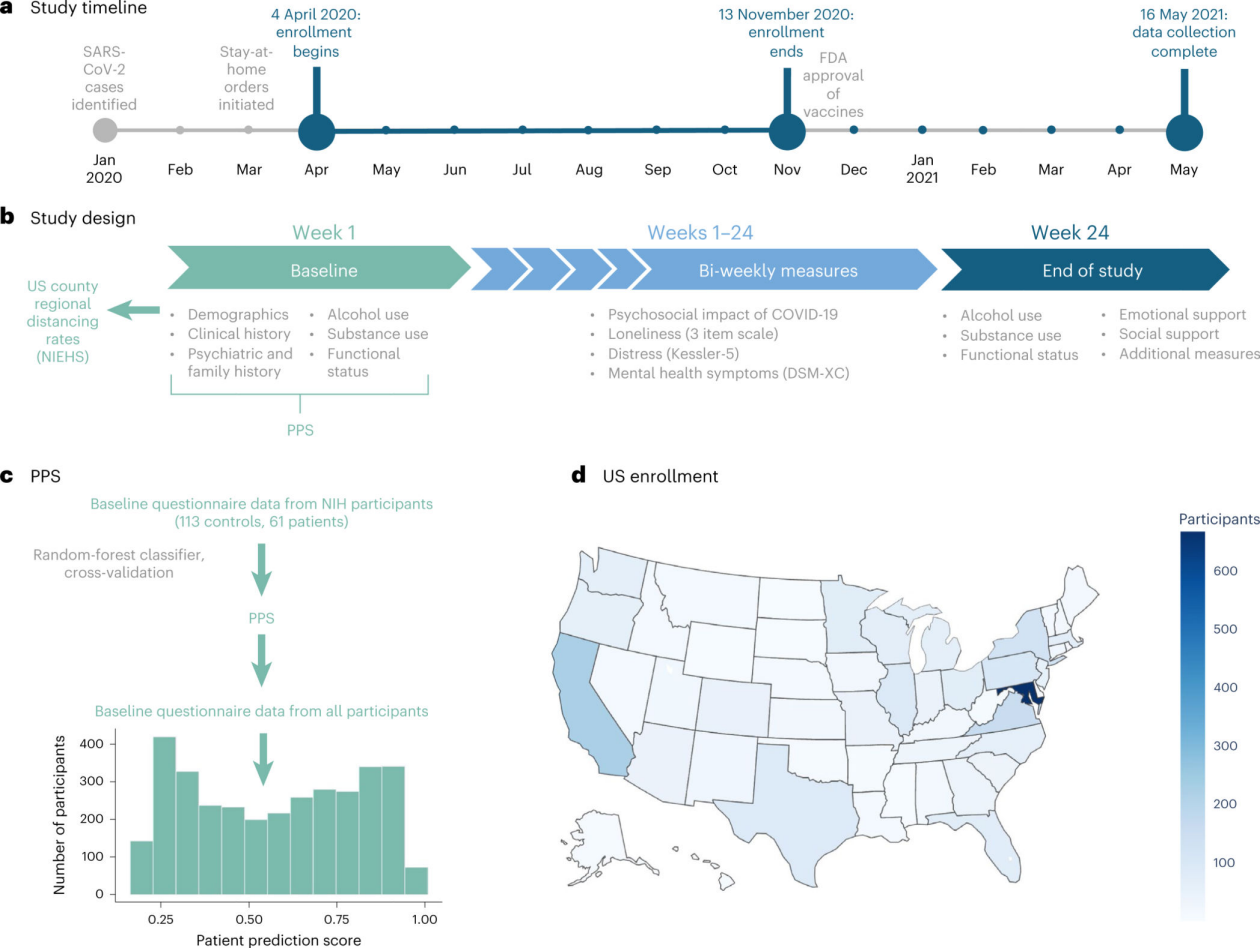
Study description. **a**, Between 4 April 2020 and 13 November 2020, 3,655 participants enrolled in a 6 month study that consisted of a set of internet-based questionnaires to be completed every 2 weeks. Data collection proceeded from April 2020 through May 2021. **b**, Participants were invited to complete questionnaires for 24 weeks. At each interval, participants were asked to complete the psychosocial impact of COVID-19 survey, which included questions about social context as well as the three-item loneliness scale,^44^ as well as the Kessler-5^40^ and DSM-XC.^26^ Additional questionnaires were administered at baseline, which were used to compute a PPS and regional estimates of social distancing based on zip code (Methods), and at the end of the study. The current paper focuses on the relationship between loneliness and psychological distress, and whether these factors vary as a function of one’s likelihood of having a psychiatric diagnosis and social distancing. **c**, Baseline questionnaire data from 174 participants who had previously undergone structured clinical interviews for diagnosis at NIH were used to construct a classifier to predict each participant’s likelihood of having had a psychiatric diagnosis. This classifier was applied to baseline questionnaire data from all participants to generate a PPS for each individual. For complete details, see ref. 18. **d**, Participants represented all US states and territories, as well as 16 countries outside of the United States. Zip code information for US participants (*n* = 3,614) was used to supplement self-report data with regional estimates of social distancing based on cell phone mobility data (Methods). PPS, patient probability score; NIEHS, National Institute of Environmental Health Sciences.

Between 4 April and 13 November 2020, 3,655 participants enrolled in the study. Participants were primarily white (90.81%) and female (80.55%), ranging from 18 to 87 years old (mean (*M*) = 46.64, s.d. = 14.85). Most respondents (98.9%; *n* = 3,614) were located in the United States or its territories (Fig. 1). Forty-one participants enrolled from 16 different countries across Europe, South America and Africa.

Patient probability score (PPS), our measure of psychiatric vulnerability, varied on the basis of gender, racial identity, setting, education and age, but not ethnicity (Table 1). We thus included demographic factors as covariates in all models. There was no difference in PPS as a function of whether individuals lived in the United States (US participants: *M* = 0.56, s.d. = 0.22, range = 0.21–0.97; non- US participants: *M* = 0.58, s/d/ = 0.21, range = 0.23–0.93; *t* = 0.65, *P* > 0.5). PPS scores were slightly higher in those living alone than in those living with others (living alone: *n* = 768, *M* = 0.58, s.d. = 0.22; living with others: *n* = 2,813, *M* = 0.56, s.d. = 0.22; *t* = 2.47, *P* = 0.014), but PPS did not vary as a function of household size (*P* > 0.6). Thus we conclude that psychiatric vulnerability and social isolation are distinct factors. The following analyses ask whether these factors impacted time-varying mental health during the first year of the pandemic.

### Distress is linked to psychiatric vulnerability and loneliness

**Fig. 2 |:**
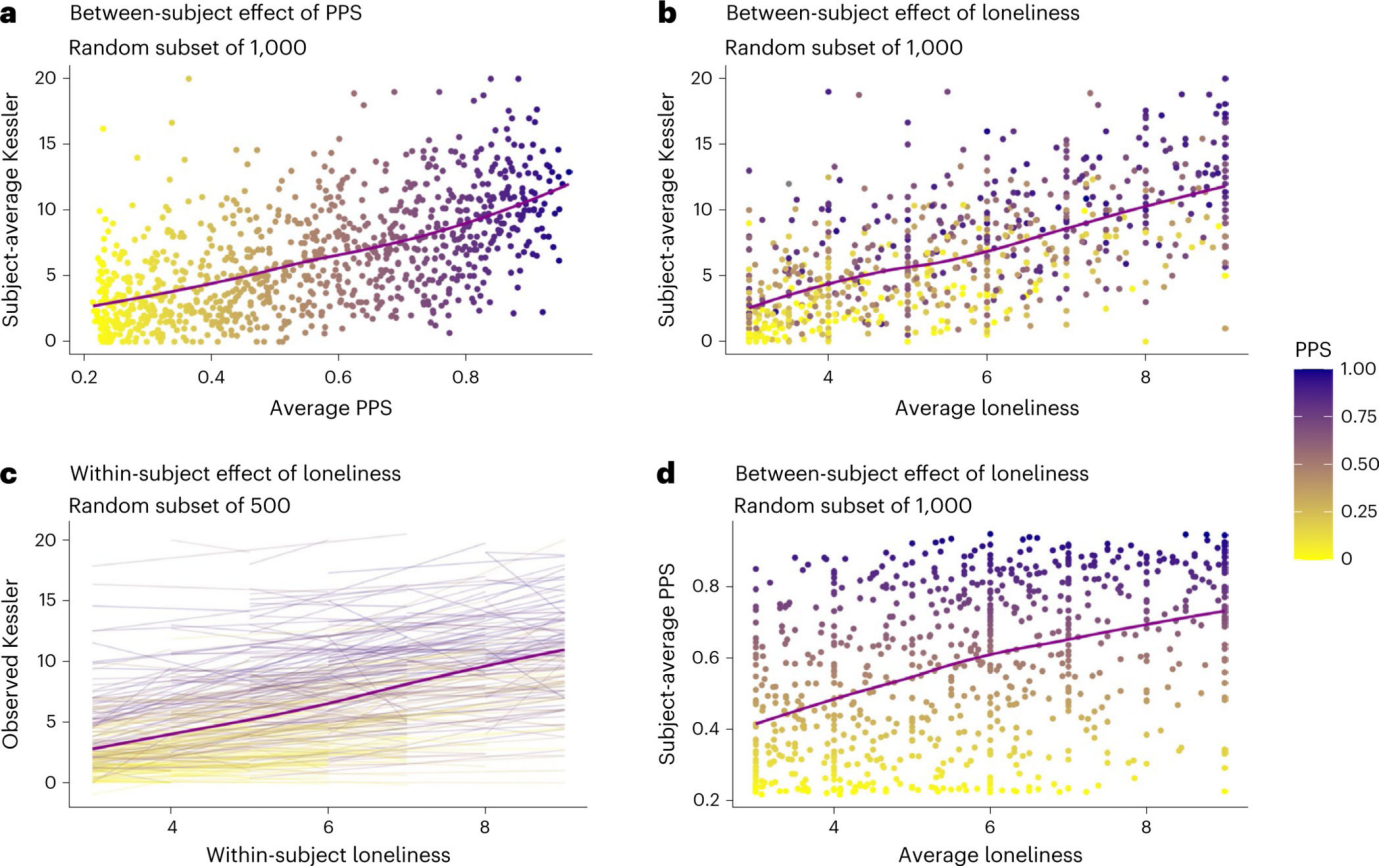
Associations between distress, loneliness and PPS over time during the COVID-19 pandemic. Analyses focused on psychological distress, a mental health outcome measure operationalized through biweekly responses on the Kessler-5 scale^40^, as a function of time, loneliness and PPS. Each figure depicts a random subset of participants, with locally estimated scatterplot smoothing regression to capture the overall trend (purple line). We depict only findings from linear mixed models that were practically significant based on Bayesian models (<2.5% of posterior estimates in region of partial equivalence (ROPE^57^)) and statistically significant at **P** < 0.001 (two-sided) in frequentist models to account for the large sample size. Multiplicity correction was not applied. For complete results, see Table 2. **a**, PPS was positively associated with average psychological distress (B = 0.77(0.02), confidence interval (CI): [0.72, 0.82], **P** < 0.001; 0% in ROPE), such that mean psychological distress across time was 0.77 units higher in individuals with a likely diagnosis (purple) relative to those likely to have no diagnosis (yellow) based on PPS. **b**, We observed positive associations between average loneliness and average distress (B = 0.95(0.03), CI: [0.89, 1.00], **P** < 0.001; 0% in ROPE), such that individuals with 1 unit higher loneliness reported 0.95 units higher distress across time. **c**, Changes in loneliness over time within individuals were also positively associated with changes in psychological distress (B = 0.57(0.02), CI: [0.53, 0.60], **P** < 0.001; 0% in ROPE), such that an increase of 1 unit loneliness was associated with an increase of 0.57 units distress at that time. **d**, Although psychological distress was positively associated with both loneliness and PPS, associations between PPS and average loneliness were only moderately correlated (**r** = 0.44, **P** < 0.001), indicating that psychiatric vulnerability and loneliness capture separate constructs.

Our main longitudinal model focused on psychological distress as a measure of mental health during the pandemic (Fig. 2). Across individuals, average psychological distress was positively associated with PPS (B = 0.77 (0.02), *P* < 0.001; *b* = 0.42; Fig. 2a), such that psychological distress was 0.77 units higher in individuals with a likely diagnosis (PPS = 1; purple in Fig. 2) relative to those likely to have no diagnosis (PPS = 0; yellow in Fig. 2), and this effect was practically significant based on Bayesian models. Although frequentist models indicated that average psychological distress decreased over time across individuals, and that the association between PPS and psychological distress varied as a function of average participation date and decreased over time within individuals (Table 2), main effects of time and interactions between PPS and time were consistent with the null hypothesis based on Bayesian models. Together, these findings suggest that there were no practically significant shifts in psychological distress as a function of time during the early pandemic. Instead, distress varied across individuals in relation to one’s likelihood of having had a psychiatric diagnosis at baseline.

Although psychological distress and its association with psychiatric vulnerability (PPS) were stable across time, we observed robust associations between psychological distress and loneliness, which varied both across individuals and within individuals over time. Lonelier individuals reported higher average distress (coefficient (B ) = 0.95(0.03), *P* < 0.001; standardized coefficient (*b*) = 0.42; Fig. 2b) and changes in loneliness within individuals were positively associated with distress (B = 0.57(0.02), *P* < 0.001; *b* = 0.26; Fig. 2c). Both effects were practically significant. Although we observed interactions between within-subjects and between-subjects loneliness and between loneliness and duration based on frequentist statistics, these interactions were consistent with the null hypothesis based on Bayesian models (Table 2). Since loneliness and PPS were both associated with distress, we evaluated their pairwise correlation: average loneliness and PPS were only moderately correlated (*r* = 0.44; Fig. 2d), indicating that they were independent predictors.

Psychological distress also varied across demographic categories, such that men reported lower average distress than women (B = −0.48(0.12), *P* < 0.001; *b* = *−*0.04), and average distress was lower in individuals with advanced professional degrees than in those with less than a bachelor’s degree (B_BS_ = 0.57(0.15), *P* < 0.001; *b* = 0.05), although these effects were undecided practical significance (Table 2). We observed additional associations with age that were significant in frequentist but not Bayesian statistics (Table 2). Associations with education, racial identity and ethnicity did not survive our conservative statistical threshold and were of undecided practical significance (Table 2), although we note that our sample was not equally balanced across demographic groups, as we address in ‘Discussion’. We evaluated formal interactions with gender and age in Supplementary Results but observed no practically significant interactions (Supplementary Table 3). Finally, associations with distress were essentially identical when we controlled for regional fluctuations in pandemic-related factors within US-based participants, as captured by NIEHS Pandemic Vulnerability Index (PVI; Supplementary Table 4), although we note that we observed interactions between PVI, PPS and time, such that psychologically vulnerable individuals in areas with high pandemic vulnerability reported the largest reductions in psychological distress across time (Supplementary Fig. 5). Associations with PPS, loneliness and time were largely consistent when we evaluated other mental health outcomes (Supplementary Tables 5 and 6) or focused on clinically significant mental health (Supplementary Table 7).

### Social isolation impacts loneliness and distress

**Extended Data Fig. 1 |:**
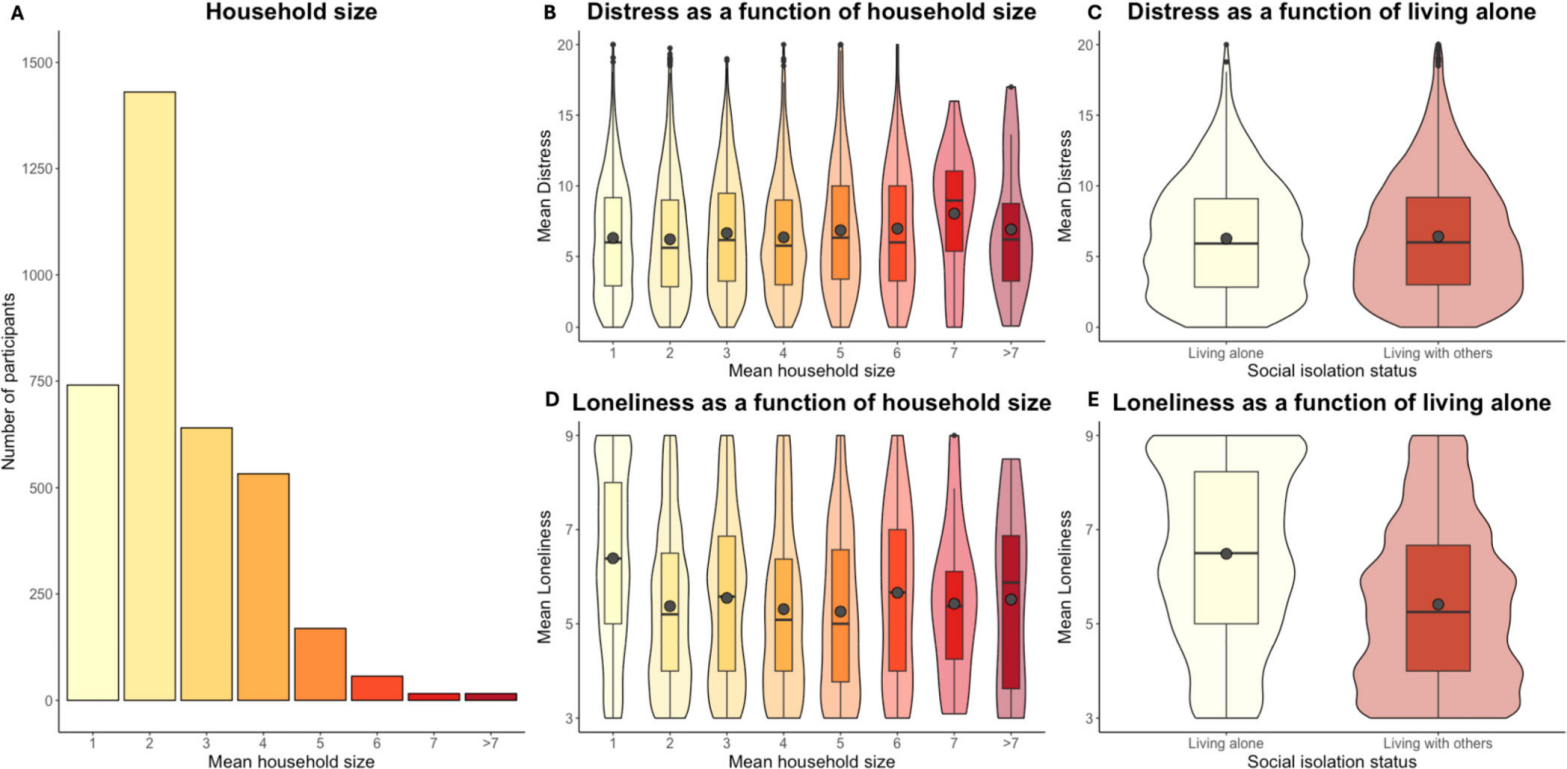
Associations between social isolation, distress, and loneliness. To differentiate between subjective loneliness and objective social isolation, we measured associations between objective social isolation, distress, and loneliness. Social isolation was treated both continuously (Household size) and categorically (Living alone vs Living with others). We report results of linear mixed models using both frequentist statistics (thresholded at p < .001 two-sided, without multiplicity correction) and Bayesian statistics (practical significance defined as <2.5% of posterior estimates in region of partial equivalence [ROPE].^57^) Boxplots present medians, first and third quartiles, and 1.5 × the interquartile range (whiskers). Gray circles denote the mean for each category. **A**) Histogram of mean household size. 768 participants (21.36%) of participants reported that they lived alone at baseline; 649 participants reported living alone at every timepoint throughout their participation. **B**) Psychological distress was positively associated with household size (B = 0.29, CI = [0.19, 0.40], p < .001), such that an increase of one additional household member was associated with an increase of 0.29 units of distress, although this effect was consistent with the null hypothesis based on Bayesian models (99.8% in ROPE; see Supplementary Table 8). **C**) Respondents who lived alone reported less distress than those who lived with others (B = −0.56, CI = [−0.76,− 0.39], p < .001), and this effect was of undecided significance based on Bayesian models (10.69% in ROPE; Extended Data Table 1). Loneliness still predicted distress when controlling for Household Size or Living Alone (see Extended Data Table 1 and Supplementary Table 8). **D**) In contrast to Psychological Distress, Loneliness was negatively associated with household size (B = −0.28, CI = [−0.33, −0.22], p < .001), such that an increase of one additional household member was associated with a reduction of 0.28 units loneliness, and this effect was practically significant (0.11% in ROPE; see Supplementary Table 9). **E**) Consistent with results of continuous models, individuals who lived alone reported an increase of 0.49 units loneliness compared to those living with others (B = 0.49, CI = [0.46, 0.52], p < .001), and differences were practically significant (0% in ROPE; see Extended Data Table 2).

Variations in loneliness were robustly associated with psychological distress during the pandemic, independent of psychiatric vulnerability. To gain further insight on the role of social context, we evaluated the impact of objective social isolation, which is distinct from loneliness. Social isolation was indexed both categorically (that is, whether a respondent was currently living alone or with others) and continuously (that is, household size). Extended Data Fig. 1 depicts distributions of household size and relationships with distress and loneliness. We focus on categorical effects of social isolation (that is, living alone versus living with others) in the main paper and report associations with household size in Supplementary Tables 8 and 9. When controlling for all other factors, individuals living alone reported less psychological distress than those living with others, who were the intercept in these categorical models (B = −0.56, *P* < 0.001; *b* = −0.11); this effect was of undecided practical significance (10.69% in region of partial equivalence (ROPE); Extended Data Table 1). Variations in loneliness both within and across individuals still predicted psychological distress when controlling for whether an individual lived alone (Extended Data Table 1). The effect of living alone did not vary as a function of time; neither did it interact with loneliness or PPS based on our conservative statistical thresholds. For complete results, see Extended Data Table 1.

We also asked how objective social isolation impacted self-reported loneliness (Extended Data Table 2). In contrast to psychological distress, individuals who lived alone reported higher loneliness than those who lived with others (B = 0.49, *P* < 0.001; *b* = 0.21; Extended Data Fig. 1), and this effect was practically significant. Although we observed statistically significant interactions between living alone, PPS and time, Bayesian analyses indicated that interactions were not sufficient to reject the null hypothesis. As reported in Supplementary Table 9, findings were consistent when we evaluated associations between loneliness and household size. We report associations with other social factors (relationship quality, social support and emotional support) in Supplementary Results (Supplementary Tables 10–13).

### Impact of social distancing on loneliness and distress

**Extended Data Fig. 2 |:**
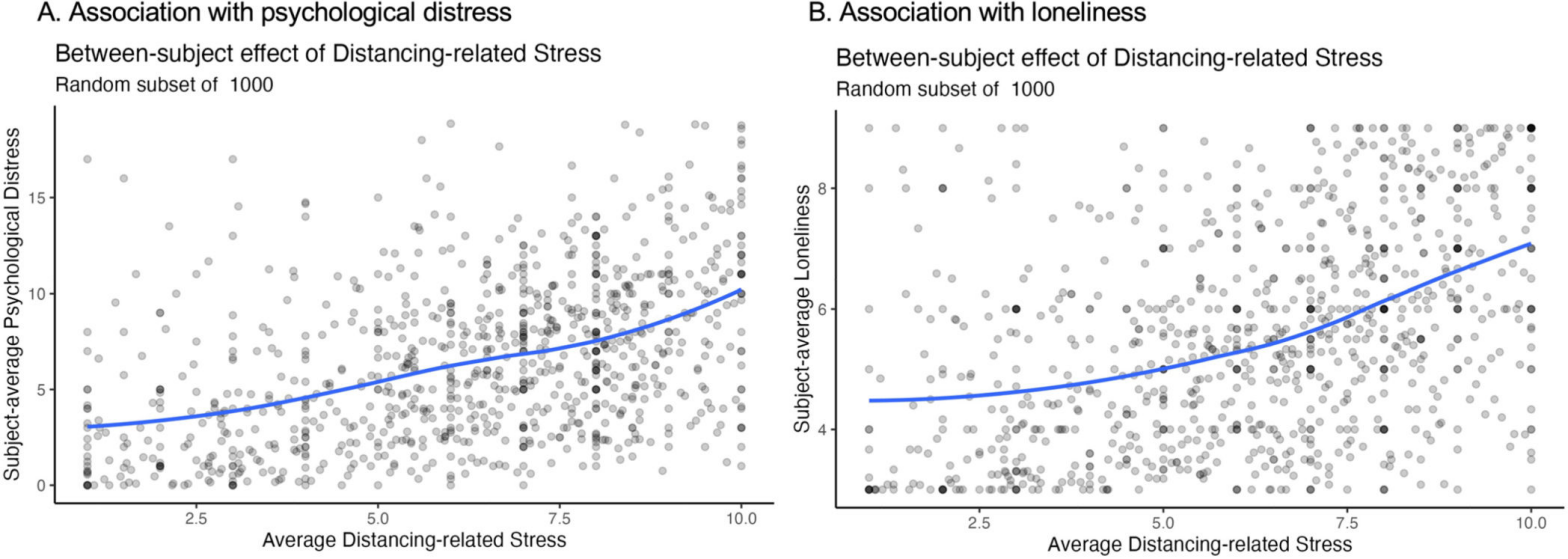
Impact of social distancing-related stress. We evaluated associations between self-reported social distancing and mental health during the pandemic. Each figure depicts a random subset of 1000 participants. Although we observed statistically significant associations with numerous measures of distancing based on linear mixed models using frequentist statistics (see Supplementary Tables 14–17), the only practically significant predictor of psychological distress and loneliness based on Bayesian statistics was individual differences in self-reported stress associated with distancing. We note that p-values are two-sided and do not include multiplicity correction. **A**) Individuals who reported higher average distancing-related stress also reported higher psychological distress across the pandemic (B = 0.5, CI = [0.44, 0.55], p < .001), such that an increase in one unit of average distancing-related stress was associated with 0.5 units higher psychological distress. This effect was practically significant when modeled alone (0.13 in ROPE; see Supplementary Table 14) and of undecided significance when controlling for other social distancing measures (4% in ROPE; see Extended Data Table 2). **B**) We also observed positive associations across individuals between mean distancing-related stress and mean loneliness (B = 0.25, CI = [0.23, 0.28], p < .001), such that an increase in one unit of average distancing-related stress was associated with 0.25 units higher loneliness. This effect was practically significant based on Bayesian models whether or not other distancing measures were included in the model (0.02% in ROPE; see Extended Data Table 3 and Supplementary Table 15).

Although social isolation impacted loneliness and psychological distress, associations may not be specific to the pandemic. We next asked whether pandemic-related social distancing was associated with psychological distress and loneliness. When we tested self-reported social distancing items independently, between-subject variations in distancing-related stress (that is, responses to the item ‘How stressful has it been for you to maintain social distancing?’) were positively associated with both psychological distress and loneliness (Supplementary Tables 14 and 15). This was also the case when all distancing measures were included in the same model: distancing-related stress was positively associated with psychological distress (B = 0.5, *P* < 0.001; *b* = 0.3; Extended Data Fig. 2a and Extended Data Table 3) and loneliness (B = 0.25, *P* < 0.001; *b* = 0.34; Extended Data Fig. 2b and Extended Data Table 4). Associations between distancing-related stress and loneliness were practically significant in both analyses (Extended Data Table 4 and Supplementary Table 15), while associations between distancing-related stress and psychological distress were practically significant when tested independently (Supplementary Table 14) and of undetermined significance (4% in ROPE) when controlling for other distancing measures (Extended Data Table 3). Although we observed additional associations with other self-reported distancing measures both across individuals and within individuals over time based on frequentist models, all within-subject effects were consistent with the null hypothesis based on Bayesian models (Extended Data Tables 3 and 4 and Supplementary Tables 14 and 15). See Supplementary Tables 16 and 17 for interactions between self-reported distancing, age and gender.

**Fig. 3 |:**
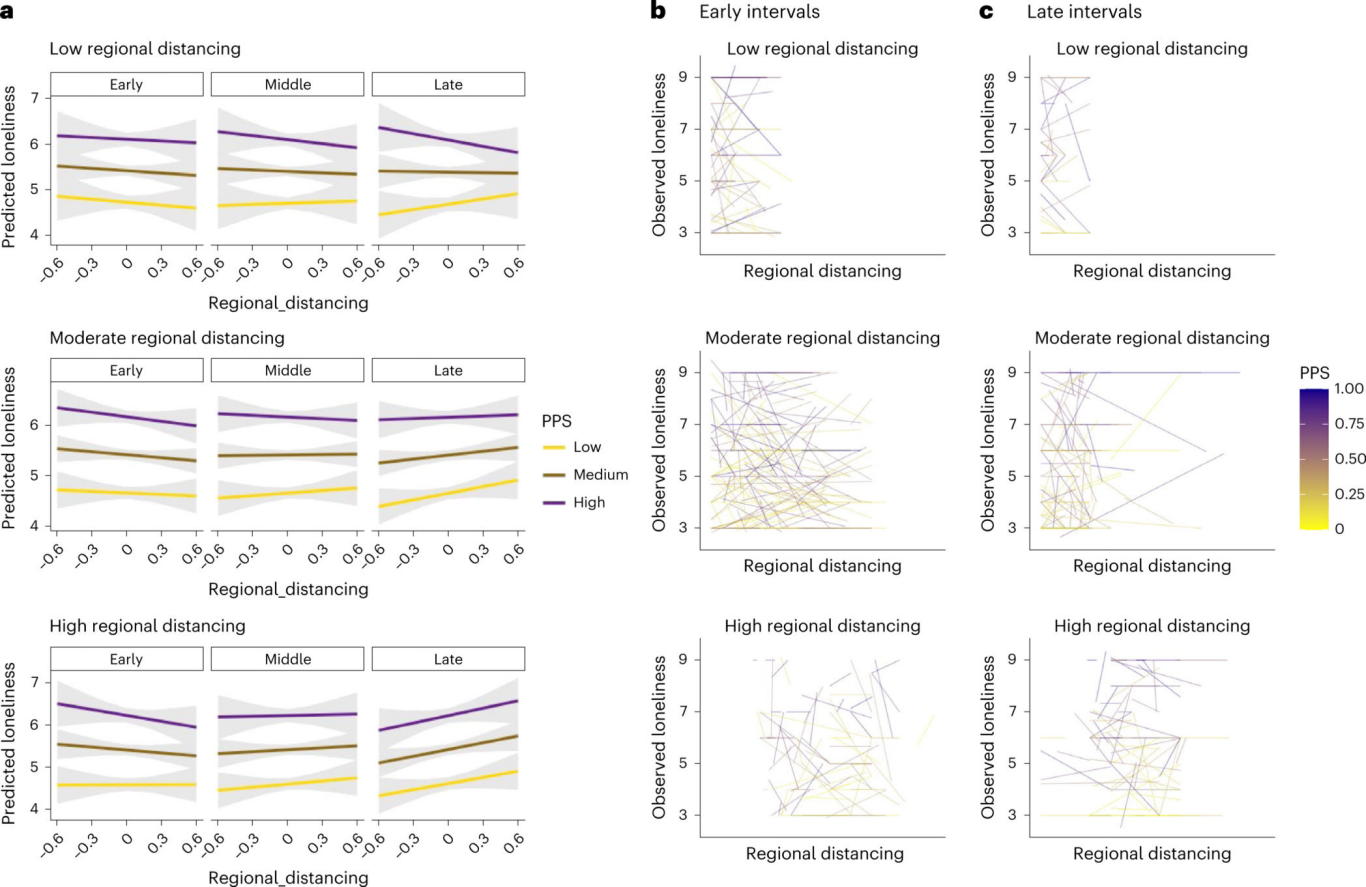
Relationships between psychiatric vulnerability, regional distancing and loneliness vary over time. We used linear mixed models to evaluate associations between regional estimates of social distancing, time and loneliness as a function of PPS (Extended Data Table 5). **a**–**c**, Model predictions (**a**) and observed data (**b**,**c**) as a function of average regional distancing based on quartiles. Models revealed interactions between all factors (B = 0.64, CI: [0.36, 0.91], **P** < 0.001, 0.08% in ROPE), such that individuals in communities of low regional distancing (top rows) exhibited stable relationships over time, reporting higher loneliness at times of less distancing, whereas individuals in regions with higher rates of distancing (bottom row) showed changes in associations between distancing and loneliness over time. This effect also interacted with PPS (B = 0.39, CI: [0.27, 0.51], **P** < 0.001, 0.06% in ROPE): individuals with low likelihood of having a psychiatric diagnosis based on PPS (yellow) showed changes in the relationship between distancing and loneliness over time regardless of regional distancing rates, such that more regional distancing was associated with higher loneliness in late intervals, whereas those with a high probability of having a psychiatric diagnosis based on PPS (purple) showed no change over time if they lived in areas with low rates of social distancing (top row), and generally reported more loneliness at times of less distancing in the community. Panel **a** depicts marginal effects, with error bands representing the 95% confidence intervals. Spaghetti plots in panels **b** and **c** reflect linear regressions between regional distancing and loneliness for each of 700 randomly selected participants as a function of regional distancing. Panel **b** depicts relationships during early enrollment (first quartile of intervals) while panel **c** depicts late enrollment (fourth quartile of intervals). All **P** values are two-sided and do not include multiplicity correction.

There were no significant associations between regional distancing and psychological distress (Supplementary Table 18). When we examined associations between regional distancing and loneliness (Extended Data Table 5), we observed practically significant interactions between duration, regional distancing per observation and average regional distancing (B = 0.64, *P* < 0.001; *b* = 0.02), such that the association between distancing and loneliness was consistent over time for individuals in areas of low distancing, whereas associations increased over time for individuals in communities with higher rates of distancing. We also observed a practically significant four-way interaction between these factors and PPS (B = 0.39, *P* < 0.001; *b* = 0.02), such that psychiatrically vulnerable individuals in communities of low regional distancing (purple lines in top row of Fig. 3) reported greater loneliness at times of less distancing across the entire study, whereas participants in areas with more distancing (Fig. 3, bottom) showed increases in associations between regional distancing and loneliness across time, as did those who had low likelihood of being patients regardless of regional distancing (yellow in Fig. 3).

### Mediation by loneliness

**Fig. 4 |:**
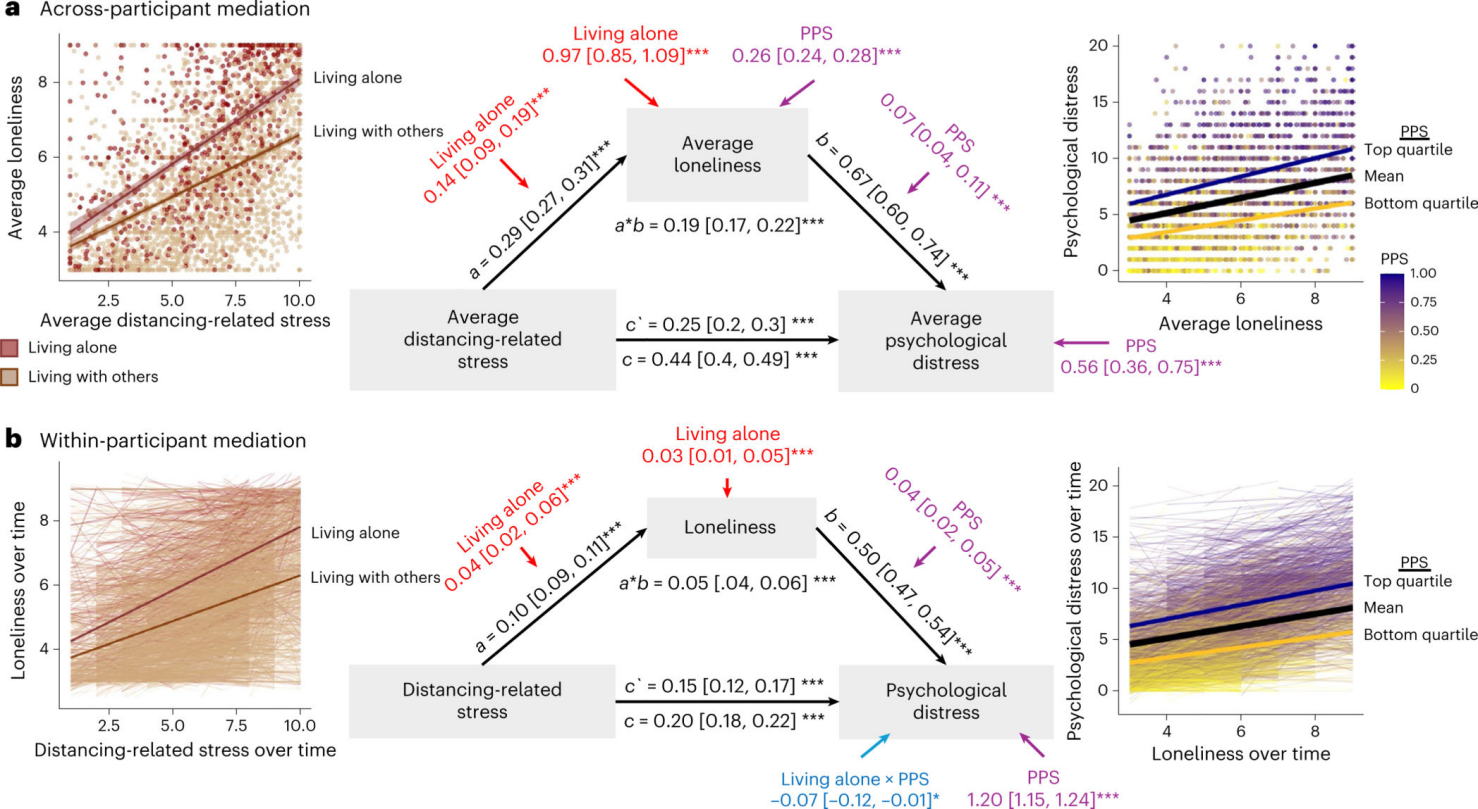
Loneliness mediates associations between self-reported distancing and psychological distress. We used mediation analyses to evaluate whether the relationship between distancing-related stress and psychological distress was mediated by changes in loneliness. PPS and living alone were treated as moderators, and we report results with and without moderators in Extended Data Table 6. Coefficients in Fig. 4 depict results from models including moderators. **a**, Single-level mediation was used to examine relationships across participants. There was a significant association between distancing-related stress and loneliness (path **a**), such that individuals who reported higher distancing-related stress on average also reported higher loneliness. This effect was moderated by living alone, such that associations were stronger in those living alone (dark red) than in those living with others (tan); error bands denote standard error of the means. Loneliness was positively associated with distress, while controlling for distancing-related stress (path **b**), such that lonelier individuals reported higher psychological distress regardless of distancing-related stress. This effect was moderated by PPS, such that associations were stronger in individuals with who were more vulnerable. We observed significant mediation by loneliness (path a*b), such that individual differences in loneliness explained 43.5% of the variance between distancing-related stress and psychological distress. **b**, We used multilevel mediation to examine whether changes in loneliness also mediated dynamic associations between distancing-related stress and psychological distress over time within individuals. Results were highly consistent with findings across individuals. Participants reported higher loneliness at time points when they reported higher distancing-related stress (path **a**), and this effect was stronger in those living alone. Fluctuations in loneliness were positively associated with psychological distress when controlling for distancing-related stress (path **b**), and these within-person associations were stronger for individuals with high PPS scores. Finally, the dynamic association between distancing-related stress and psychological distress was reduced when controlling for fluctuations in loneliness (path a*b), such that loneliness explained 25.5% of the total effect. All **P** values are two-sided and do not include multiplicity correction. ***, **P** < .001; *, **P** < .05.

Our analyses indicate that stress associated with social distancing was associated with both loneliness and psychological distress. We next asked whether loneliness formally mediated the relationship between distancing-related stress and psychological distress by measuring associations across individuals (that is, single-level mediation) and within individuals over time (that is, multilevel mediation). We also used moderated mediation to ask whether relationships between distancing-related stress, loneliness and psychological distress varied as a function of PPS and/or social isolation. As mediation results were similar whether or not moderators were included, we focus on results of moderated mediation (Fig. 4). Extended Data Table 6 reports complete results with and without moderators.

Path *a* captures the association between distancing-related stress and loneliness. We observed positive path *a* effects both across individuals (path *a* = 0.29, *P* < 0.001; Fig. 4a) and within individuals over time (path *a* = 0.10, *P* < 0.001; Fig. 4b). In both models, living alone moderated path *a* (Extended Data Table 6), such that individuals who lived alone (dark red in Fig. 4) showed stronger positive associations between distancing-related stress and loneliness than those who lived with others (tan in Fig. 4). There was no association between path *a* and PPS, and no interactions between PPS and living alone, but we observed positive associations between average loneliness and both PPS and living alone when controlling for distancing-related stress in our between-subjects mediation (Fig. 4a), and between average loneliness and living alone in the within-subjects mediation (Fig. 4b).

Path *b* captures the relationship between loneliness and psychological distress when controlling for distancing-related stress. We observed significant path *b* effects both across individuals (*b* = 0.67, *P* < 0.001) and within individuals over time (*b* = 0.50, *P* < 0.001). PPS moderated path *b* (Extended Data Table 6), such that the association between loneliness and distress, when controlling for distancing-related stress and social isolation, was strongest for individuals with high PPS scores, whether we examined variations across individuals (Fig. 4a) or within individuals over time (Fig. 4b). Associations with PPS are visualized in the right panels of Fig. 4. Associations between living alone and path *b* did not survive our conservative statistical threshold but are included in Extended Data Table 6 for completeness.

Path *c’* captures direct effects, or the association between distancing-related stress and psychological distress when controlling for loneliness (as well as PPS and living alone). We observed reductions from the total effect (c, consistent with results reported in the preceding) to the direct effect (c’) whether we measured associations across individuals (total effect: *c* = 0.44, *P* < 0.001; direct effect: c’ = 0.25, *P* < 0.001) or within individuals over time (total effect: *c* = 0.21, *P* < 0.001; direct effect: c’ = 0.14, *P* < 0.001). These reductions were consistent with mediation in both models based on the magnitude of the indirect effect through loneliness (that is, path a*b; Extended Data Table 6). Loneliness accounted for 43.5% of the variance between distancing-related stress and psychological distress across participants (indirect effect a*b = 0.19, *P* < 0.001) and 25.46% of the variance between distancing-related stress and psychological distress within participants over time (indirect effect a*b = 0.05, *P* < 0.001). PPS and living alone did not moderate the direct effect or indirect effect in either model (although we saw associations between PPS and the indirect effect in the across-subjects model that did not survive our conservative threshold; see Extended Data Table 6), suggesting that loneliness explained associations between distancing-related stress and psychological distress similarly regardless of social isolation or psychiatric vulnerability.

Finally, we observed main effects of PPS on psychological distress when controlling for distancing-related stress, loneliness and living alone in both models (Fig. 4), driven by positive associations between PPS and psychological distress. The multilevel mediation also revealed a negative PPS × living alone interaction that did not survive our stringent statistical threshold but was significant in Bayesian models (Fig. 4). As reported in Supplementary Results, reverse mediation models that tested whether distancing-related stress mediated associations between loneliness and psychological distress provided worse descriptions of the data than the models we report here (Supplementary Table 19).

## Discussion

We examined the impact of psychiatric vulnerability and loneliness on psychological distress during the first year of the COVID-19 pandemic in a large cohort of individuals, over half of whom reported previous psychiatric treatment. Psychiatric vulnerability upon enrollment, operationalized by a classifier trained on data from patients who were clinically evaluated before the pandemic, was a robust predictor of subsequent psychological distress over the course of the pandemic. Loneliness, which varied both within and across individuals, was also strongly associated with distress. Stress associated with social distancing was associated with both distress and loneliness, and loneliness mediated associations between distancing-related stress and mental health, as operationalized by psychological distress, both across individuals and within individuals over time. Here we discuss these findings and outstanding questions.

Psychological distress was strongly associated with baseline psychiatric vulnerability, as operationalized by PPS. Building on our initial preprint,^1^ we validated PPS against self-reported psychiatric treatment history in the entire sample and related PPS with self-reported treatment history, indicating that training a classifier on patients who were clinically evaluated in person before the pandemic can indeed estimate the likelihood of previous psychiatric diagnosis upon enrollment. Bayesian analyses indicated that there were no practically significant changes over time in the association between PPS and psychological distress or our other mental health outcomes. Thus, one’s psychiatric vulnerability at baseline was a stable predictor of mental health during the first year of the pandemic. This has been confirmed using cross-validation and data-driven analyses of other mental health factors in the current dataset^19^ and builds on studies that evaluate the influence of pre-existing mental health conditions during the early phase of the pandemic.^5,10,20^

Loneliness is a well-documented risk factor for impaired mental health, physical health and reduced longevity.^13,14,21,22^ Our data support associations between loneliness and mental health, as psychological distress was higher when individuals felt lonelier, and lonely individuals reported more distress on average across the first year of the pandemic. These findings build on studies that report associations between loneliness and mental health in the first few months of the pandemic.^23–25^ Importantly, associations between loneliness and psychological distress were as strong as the association between psychological distress and baseline psychiatric vulnerability. Although our findings are correlational and therefore preclude causal inference, these strong positive associations support the efforts of various public health agencies to target loneliness and social isolation in the interest of improving mental health.^26,27^

The relationship between loneliness and mental health might reflect pandemic-related changes in social connectedness. We investigated this by measuring (1) objective social isolation and (2) self-reported and community-based social distancing. Social isolation is distinct from loneliness; individuals with large social networks can still feel lonely, and individuals who have few social connections may not feel lonely. Although social isolation was associated with distress and loneliness, loneliness still predicted distress while controlling for social isolation or household size. Interestingly, participants reported less loneliness but more distress as a function of living with others and increasing household size. This is consistent with other work on the impact of stay-at-home orders on family dynamics and conflict^28,29^ due to new challenges such as distance learning, remote work and reduced social engagements. Our findings indicate that these factors also have deleterious impacts on mental health.

Associations between distress, psychiatric vulnerability, social isolation and loneliness are unlikely to be unique to the pandemic. To explore whether pandemic-specific factors were associated with psychological distress and loneliness, we focused on social distancing, based on self-report and community-based data. Although we observed associations with multiple measures of distancing, one’s average stress associated with social distancing was the only practically significant predictor of psychological distress, together with loneliness and likelihood of having a psychiatric diagnosis. Mediation analyses revealed that the relationship between distancing-related stress and psychological distress was mediated by changes in loneliness, whether we examined variations across individuals or within individuals over time. Our findings suggest that those who experience more stress in response to distancing report higher psychological distress in part due to increased loneliness. In other words, when individuals felt lonely in response to distancing-related stress, they reported greater distress than when they did not feel lonely in response to distancing. Moreover, respondents who lived alone exhibited stronger associations between distancing-related stress and loneliness, while psychiatrically vulnerable individuals exhibited stronger associations between loneliness and psychological distress. These findings further support the idea that loneliness is a key target for intervention, and that addressing social connectedness might prevent social isolation from affecting mental health, consistent with several recent efforts aimed at reducing suicide risk in older adults.^30–32^ We note that our mediation analysis depends on measures that were collected at the same time and thus preclude causal inference. Although we explored reverse mediation models and results support the hypothesized directionality, future analyses would benefit from considering time-lagged analyses to determine whether distancing leads to loneliness, which in turn leads to psychological distress.

Finally, when we evaluated regional estimates of distancing in US-based participants rather than self-reported measures, we observed that associations between regional distancing and loneliness varied over time and as a function of psychiatric vulnerability. Vulnerable individuals in regions with less distancing experienced more loneliness with less regional distancing throughout the first year of the pandemic, whereas vulnerable individuals in communities with higher levels of distancing showed changes over time and reported higher loneliness at intervals with greater regional distancing in later, but not earlier, intervals. Thus psychiatric vulnerability and social context both played a role in how distancing affected loneliness during the first year of the pandemic. We observed related findings when we used a general measure of pandemic vulnerability: psychologically vulnerable individuals in regions that were most impacted by the pandemic showed the strongest reductions in psychological distress over time.

Our study had several limitations that could be addressed by other studies and population-based work. First, our sample was not representative of the US population. Although all states were represented, participants were overwhelmingly white, female and educated. Thus, conclusions may not be generalizable to the US population at large or to other nations, and results are unlikely to reflect the population who experienced the most adverse impacts of the pandemic in the United States, namely Black and Latinx individuals^33,34^ and those of low socioeconomic status.^35^ Although we observed some variations by sociodemographic factors, Bayesian analyses suggested that these were of undecided practical significance. However, we did observe associations between demographic factors and likelihood of responding across multiple intervals, particularly for those with less than an associate degree who were less likely to respond across multiple intervals than those with higher levels of education. Our findings should be confirmed in more representative samples, as only 2.75% of our participants had less than an associate degree, 3.4% were Black/African American, and 5.6% were Latinx.

Generalizability may be further limited because the study recruited a convenience sample from existing lists of previous National Institute of Mental Health (NIMH)/National Center for Complementary and Integrative Health (NCCIH) research participants, online flyers and direct mail postcards, and was completely voluntary and uncompensated. Participants were asked to dedicate time to complete questionnaires every 2 weeks for 6 months; ultimately, 568 participants (16% of sample) completed every interval. This might have affected conclusions in two ways. First, individuals may have missed time points when they were most severely impacted, and thus distress may be underestimated by assuming that data were missing at random. Second, participants may have used the study as a coping tool during the pandemic. Many participants used a free response item within the COVID-19 Survey to describe ongoing challenges and triumphs, and reported on how helpful it was to be a participant when completing the study.^36^ Insofar as participation aided coping, analyses might underestimate the distress that would have been experienced had participants not enrolled in the study. To avoid the potential for collider bias,^37,38^ our conclusions should be confirmed with epidemiological and clinical samples that did not rely on self-selection. In addition, we focused on general mental health outcomes rather than specific psychiatric symptoms or syndromes. Conclusions should be compared with longitudinal studies that measure specific psychiatric symptoms or clinical conditions.

## Conclusions

Our large longitudinal study of mental health measured dynamic relationships between loneliness, social distancing and mental health throughout the first year of the COVID-19 pandemic. Our results indicate that likelihood of previous psychiatric diagnosis and week-to-week fluctuations in loneliness were the strongest predictors of psychological distress across time. Individuals’ responses to social distancing also predicted distress and loneliness, and the association between distancing-related stress and psychological distress was mediated by changes in loneliness. The impact of loneliness was both related to, and distinct from, the impact of objective social isolation. This highlights loneliness as a key target for interventions to improve mental health, particularly in future public health crises that require social distancing.

### Methods

#### Participants

Between 4 April and 13 November 2020, 3,655 participants provided consent. Participants were asked to complete questionnaires every 2 weeks for 6 months (13 intervals, including baseline and end-of-study measures). As participation was entirely voluntary (that is, uncompensated), participants could skip intervals if they did not wish to respond on a given week. Of those who consented and completed baseline questionnaires, 3,149 participants (86.2%) provided responses on more than one time point, and 568 participants (15.6%) completed every interval; we report associations between likelihood of repeat response and baseline characteristics in Supplementary Results and Supplementary Fig. 4. See ref. 18 for baseline data and preliminary results within participants who enrolled between 4 April and 16 May 2020 (*n* = 1,992).

#### Materials and questionnaires

Consent forms and questionnaires were administered through a secure online platform, Clinical Trials Survey System. Following consent, participants provided baseline demographics and clinical history (see ref. 18 for specific measures). Subsequently, they completed measures of mental health, social support and pandemic-specific factors every 2 weeks for 6 months (Fig. 1) and end-of-study measures at the final time point. The current paper focuses on the associations between mental health and social connectedness. Additional outcomes are considered in separate work.^19,36,39^

#### Mental health outcomes

Three items measured mental health at all intervals: (1) the Kessler-5;^40^ (2) the DSM-5 self-rated level 1 cross-cutting symptom measure-adult (DSM-XC)^26^ and (3) the patient health questionnaire (PHQ-2) (ref. 27) embedded in the DSM-XC. We computed overall scores for the Kessler-5 and the PHQ-2 and computed general mental health factor scores from a bifactor model of the DSM-XC.^41^ Pairwise correlations between the three mental health outcomes were all >0.78 (Supplementary Table 1). We therefore focused on Kessler-5, a measure of psychological distress,^40^ as our primary dependent measure of mental health. Conclusions from PHQ-2 and DSM-XC are provided in Supplementary Results, Supplementary Tables 5 and 6 and Supplementary Fig. 4.

#### Social context

Social factors were measured through a 45-item survey we developed to assess pandemic-related circumstances, behaviors and responses^42,43^. Three items assessed social distancing: How much have you been social distancing?; How stressful has it been for you to maintain social distancing?; and How much has your time with other people changed compared to how you acted before the COVID-19 outbreak? Pairwise correlations between these items were low (all *r*’s < 0.4; Supplementary Table 2), suggesting they were dissociable. To measure loneliness, we incorporated the Three-Item Loneliness Scale^44^ in the COVID-19 survey. We also asked participants to report on household size and quality of relationships. We used household size to differentiate between loneliness and objective social isolation (that is, the impact of living alone). Associations with relationship quality are reported in Supplementary Results (Supplementary Tables 10 and 11), along with self-reported social and emotional support, which was measured at the end of the study using Patient-Reported Outcomes Measurement Information System measures.^45^

#### Patient probability score

A subset of participants (*n* = 174) underwent a diagnostic interview using the Structured Clinical Interview for *DSM*^46^ at NIH before March 2020. Of these, 61 were diagnosed with a current or previous psychiatric diagnosis, and 113 had no psychiatric history. We trained a classifier on baseline responses to five questionnaires (a modified Family Interview for Genetic Studies,^47^ the World Health Organization Disability Assessment Schedule,^48^ the Alcohol Use Disorders Identification Test,^49^ the *DSM*-5 level 2 Substance Use–Adult^50^ and a demographic questionnaire) from these participants to generate PPSs^18^ for each respondent who completed all five questionnaires (*n* = 3,648). PPS ranges from 0 to 1 and corresponds to the probability of an individual having a psychiatric diagnosis at the time of study enrollment. In previous work, we validated PPS with self-reported treatment history (including previous mental health hospitalization, treatment for alcohol and/or drug abuse, and/or medication for a mental health condition) in an initial wave of participants during lockdown (*n* = 1,992)^18^ and determined the area under the curve of the receiver operating characteristic was 0.87. In the complete sample, we obtain an area under the curve of 0.86 (see Supplementary Results and Supplementary Fig. 1). PPS therefore estimates each participant’s likelihood of having received a previous psychiatric diagnosis as a summary function of the different types of information in the five questionnaires (which may include treatment history). It has the advantage of being transdiagnostic, continuous and based on data from patients who were clinically evaluated before the pandemic, thus providing more insight than self-reported treatment history or diagnosis alone. We thus used PPS to index each participant’s baseline psychiatric vulnerability. Participants without PPS scores (*n* = 7) were excluded from analyses involving PPS. For additional information on the relationship between PPS and self-reported mental health history upon enrollment, see Supplementary Results and Supplementary Fig. 2.

#### Regional distancing and PVI

At baseline, 3,046 US participants provided the first three digits of their zip code, which was used in conjunction with PVI data^51^ to examine regional COVID-19 risk and social distancing. Regional social distancing was operationalized by NIEHS ‘Social Distancing Metrics’ (‘Intervention_Social_Distancing’), based on regional cell phone mobility data.^51^ Values in the NIEHS dataset are proportional relative to the previous year, such that higher values in the dataset denote less social distancing, and outcomes are positively associated with PVI (that is, increased mobility should be associated with higher vulnerability to regional spread). In the present study, to aid interpretation and ensure consistency with our self-report data, in which higher values reflect more distancing, we reverse-scored the NIEHS values, so that our higher regional distancing values reflect more distancing in a community. In Supplementary Results, we modeled overall PVI score as a factor to ensure findings were consistent when accounting for variations in the pandemic’s public health impact and to evaluate potential interactions (Supplementary Table 4 and Supplementary Fig. 5).

#### Procedures

Study ethics were evaluated and approved by NIH’s institution review board (clinicaltrials.gov ID NCT04339790; principal investigator: J.Y.C.) and launched on 4 April 2020. We initially contacted former research participants via email from six labs of the NIMH and NCCIH Intramural Research Programs and invited them to participate. The study was also advertised online through NIMH’s social media outlets, listservs, clinicaltrials.gov and direct mail postcards. Interested parties were directed to a study website that contained a study description and a link to provide informed consent through Clinical Trials Survey System. Following consent, participants completed baseline measures that were used to derive PPS for each participant.^18^ Participants were subsequently contacted every 2 weeks and asked to complete the Psychosocial Impact of COVID-19 Survey, Kessler-5 and DSM-XC. Participants could forego any intervals or items they did not wish to complete. After 12 intervals, participants were asked to complete end-of-study measures. We sent two flyers about study progress during data collection to encourage continued participation. Enrollment concluded after 6 months (final enrollment date: 13 November 2020), leading to a sample size of 3,655 participants.

#### Statistical analyses

We used linear mixed models to evaluate associations between PPS, loneliness and psychological distress and to test whether associations varied over time. For each time point, psychological distress was treated as the outcome measure while loneliness, time and PPS were treated as predictors. Follow-up analyses used linear mixed models to evaluate whether time and PPS affected loneliness over time. We also used linear mixed models to test whether psychological distress and loneliness were impacted by social factors. We focused on social distancing and social isolation in the main paper and reported associations with household size, relationship quality and social support in Supplementary Results. We included all self-report measures of social distancing in one model in the main paper and report separate evaluations of each measure in Supplementary Results (Supplementary Tables 14–17). We evaluated associations with regional distancing separately from self-reported social distancing.

We used linear models implemented with the function ‘summary_factorlist’ from the R package ‘finalfit’^52^ to evaluate whether PPS varied on the basis of respondent demographics. Because there were indeed differences in PPS as a function of demographic factors (Results and Table 1), we included covariates for racial identity, ethnicity, gender, setting, education and age in all models. Participants who were missing age data (*n* = 45) were excluded from analyses. Covariates other than age were modeled as categorical variables, with the dominant subgroup (white non-Hispanic suburban women with advanced professional degrees) as the intercept. Analyses that tested interactions with gender (restricted to men and women) and age are reported in Supplementary Results and acknowledged in the main paper when significant.

Time was decomposed into level 2 (between-subject calendar time, the number of days from initiation of the study to the individual’s mean participation date; referred to as ‘average participation date’) and level 1 (within-subject duration of study participation, the number of days from the individual’s consent date at each observation; referred to as ‘duration’) effects. The same decomposition was applied to all time-varying predictors (for example, loneliness, social distancing), which were modeled both within participants (per observation) and across subjects (subject-level average). For all models, we tested main effects of time, PPS and loneliness, and all interactions. Each analysis includes all participants with complete data for at least one time point for the specific measures of interest. As this leads to slightly different sample sizes across analyses, we provide sample sizes for each analysis in tables. Missing intervals were not included in analyses. With the exception of demographic covariates, all predictors were mean-centered before analysis. Subject-level intercepts and slopes were treated as random. Inspection of residuals and Q–Q plots from base models revealed that residuals were normally distributed (Supplementary Methods and Supplementary Fig. 3).

We used ‘lmer’ in the R package ‘lme4’^53^ to evaluate linear mixed models and used the R package ‘effectsize’^54^ to compute pseudo-standardized coefficients^28^ and compare effect size across predictors within models. Coefficients from standardized models are reported in italics (‘*b*’) to differentiate from coefficients in original units (‘B’). Due to our large sample size and the relationship between sample size and likelihood of false positives, we set a threshold for statistical significance of 0.001. To further ensure the statistically significant findings were robust and unlikely to be due to chance, we employed Bayesian analyses, which are more conservative and allow researchers to determine whether evidence is sufficient to accept or reject the null hypothesis. We used the R package ‘brms’^55^ to confirm results from our main models with Bayesian statistics. Models were fit with normal priors centered at 0 (s.d. = 2.5). We evaluated practical significance using the ROPE through the package BayesTestR,^56^ as in ref. 57. Effects were considered practically significant, that is, having enough evidence to reject the null hypothesis, when fewer than 2.5% of posterior estimates were in the ROPE, and the null hypothesis was accepted when more than 97.5% of posterior estimates were in the ROPE. We report both frequentist and Bayesian statistics for ease of interpretation, and only make inferences on findings that were both statistically significant in frequentist models and were practically significant based on Bayesian analyses; complete results are reported in tables.

We used mediation analysis to ask whether loneliness mediated the dynamic relationship between social distancing-related stress and psychological distress. We evaluated single-level mediation, which examines associations across individuals, and multilevel mediation, which examines associations within individuals over time. Single-level mediation was implemented with the ‘mediate’ and ‘test.modmed’ functions from R toolbox mediation,^58^ which includes non-parametric bootstrapping. Bayesian multilevel mediation was implemented with the R toolbox ‘bmlm’^59,60^ for models without moderators, and through ‘brms’ for models with moderators. We used bootstrapping to evaluate significance of the mediation effect^61^ and report results of Bayesian hypothesis testing based on posterior estimates as well as frequentist statistics. Our main model assumed that social distancing-related stress was the input variable (*X*), and psychological distress was the output variable (*Y*), and we tested for mediation by self-reported loneliness (*M*). We also tested whether PPS and social isolation (that is, the effect of living alone) moderated any paths. Multilevel analyses included random intercepts and slopes, as well as a within-subjects factor for time. Results were evaluated using the ‘hypothesis’ function of ‘brms’^55^ (using an alpha value of 0.001, consistent with our other results), results from ‘lmer’ within the lme4 package^53^ and the ‘mediation’ function of ‘bayestestR,’^56^ with the exception of tests for moderated mediation (that is, associations between the mediation effect a*b and moderators), which were computed using linear regression. Participants were included in mediation analyses if they had at least one time point with valid data for all three measures. Sample sizes for each analysis are provided in Extended Data Table 6. We evaluated reverse mediation models (Supplementary Methods) and report model comparisons between hypothesized and reverse models in Supplementary Results and Supplementary Table 19.

#### Reporting summary

Further information on research design is available in the Nature Portfolio Reporting Summary linked to this article.

## Extended Data

**Extended Data Table 1 |:** Longitudinal model of distress as a function of loneliness and social isolation

	Term	Coefficient	95% CI	Coefficient (stand.)	95% CI (stand.)	t-statistic	df	p.value	% In ROPE
Main effects	**(Intercept)**	6.40	[6.23, 6.57]	0	[0.00, 0.00]	75.08	3631	0	0%
	**Average loneliness**	1.00	[0.93, 1.06]	0.44	[0.42, 0.47]	30.91	4278	0	0%
	**PPS**	0.78	[0.73, 0.83]	0.42	[0.39, 0.45]	29.48	3985	0	0%
	**Loneliness overtime**	0.56	[0.52, 0.60]	0.26	[0.24, 0.28]	26.86	2265	0	0%
	*Living alone*	−0.56	[−0.76, −0.39]	−0.11	[−0.15, −0.08]	−6.28	14340	0	10.69%
	Average participation date	−0.1	[−0.15, −0.04]	−0.06	[−0.09, −0.03]	−3.95	4161	0	100%
Interactions	Average loneliness × Living alone × PPS	0.05	[0.01, 0.10]	0.06	[0.01, 0.10]	2.49	11430	0.013	100%
	Average participation date × Duration	−0.02	[−0.04, −0.01]	−0.05	[−0.07, −0.02]	−3.84	2784	0	100%
	Average participation date × PPS	−0.04	[−0.06, −0.01]	−0.05	[−0.08, −0.02]	−3.23	4246	0.001	100%
	Average participation date × Living alone	0.09	[0.01,0.17]	0.05	[0.00, 0.10]	2.13	10520	0.033	100%
	Duration × PPS	−0.02	[−0.04, −0.01]	−0.04	[−0.07, −0.02]	−3.89	2825	0.000	100%
	Average loneliness × Duration	0.03	[0.01, 0.041	0.04	[ 0.02, 0.06]	3.36	2808	0.001	100%
	Average loneliness × Loneliness over time	0.05	[0.02, 0.08]	0.03	[0.01, 0.05]	3.42	2822	0.001	100%
	Average loneliness × Average participation date × Duration	0.01	[0.00, 0.02]	0.03	[0.01, 0.06]	2.63	2744	0.009	100%
	Average participation date × Duration × PPS	−0.01	[−0.01, 0.00]	−0.03	[−0.05,0.00]	−2.29	2789	0.022	100%
	Average loneliness × Loneliness overtime × PPS	−0.01	[−0.03, 0.00]	−0.02	[−0.04, 0.00]	−2.06	2957	0.039	100%
	Average participation date × Duration × Living Alone × PPS	0.01	[0.00, 0.02]	0.02	[0.00, 0.05]	1.97	3109	0.048	100%
	Average loneliness × Loneliness over time × PPS × Time within × Living Alone	0.02	[0.00, 0.03]	0.02	[0.01, 0.04]	2.63	19050	0.009	100%
	Loneliness over time × PPS	0.02	[0.00, 0.04]	0.02	[0.00, 0.04]	2.25	2320	0.024	100%
Covariates	*Education: Less than Associates*	0.85	[0.26, 1.37]	0.03	[0.01, 0.06]	2.94	3679	0.003	9.88%
	*Racial identity: AA*	−0.58	[−1.09, −0.10]	−0.03	[−0.05, 0.00]	−2.28	3668	0.023	30.23%
	*Education: Less than Bachelors*	0.52	[0.23, 0.80]	0.04	[ 0.02, 0.07]	3.56	3586	0	32.87%
	*Gender: Man*	−0.48	[−0.71, −0.23]	−0.04	[−0.07, −0.02]	−3.85	3431	0	42.79%
	*Ethnicity: Latino*	0.45	[0.03, 0.88]	0.03	[0.00, 0.05]	2.12	3480	0.034	49.83%
	Age	−0.03	[−0.04, −0.03]	−0.13	[−0.15, −0.10]	−10.58	3433	0	100%

This table reports results of linear mixed models predicting psychological distress as a function of Social Distancing, Time, and Patient Probability Score (PPS), while controlling for demographic categories in 3584 participants with sufficient data. Social isolation was modeled as a categorical factor, with those who lived with others as the intercept; the term ‘Living alone’ thus accounts for differences in distress as a function of living alone, relative to living with others. We report results that were significant at p < .05 for brevity. Consistent with Table 2, practically significant factors are bolded, statistically significant factors of undecided practical significance are italicized, and effects that were consistent with the null hypothesis are reported in plain text. For complete results, including factors whose p-values exceeded p =.05, see Supplementary Table 21. Results were evaluated using the following model: distress ~ Gender + Education + Ethnicity + Racial identity + Setting + Age + Time between*Time within*Loneliness between*Loneliness within*Living Alone*PPS + (1 + Time within + Loneliness within| SUBJECT_NUMBER).

**Extended Data Table 2 |:** Longitudinal model of loneliness as a function of social isolation

	term	Coefficient	95% CI	Coefficient (stand.)	95% CI (stand.)	t-statistic	df	p.value	% in ROPE
Main effects	**(Intercept)**	5.36	[5.32. 5.53]	0	[0.00. 0.00]	113.14	3569	0	0%
	**PPS**	0.32	[0.29. 0.34]	0.39	[0.36. 0.42]	24.45	3647	0	0%
	**Living alone**	0.49	[0.46. 0.52]	0.21	[0.20. 0.23]	31.82	255700	0	0%
	Average participation date	−0.03	[−0.05. −0.01]	−0.04	[−0.08. −0.01]	−2.83	3730	0.005	100%
Interactions	Average participation date × Living alone	0.09	[0.07. 0.10]	0.11	[0.09. 0.13]	11.31	207300	0	100%
	Living alone × PPS	0.06	[0.04. 0.07]	0.06	[0.05. 0.08]	8.26	250500	0	100%
	Duration × Living alone × PPS	0.02	[0.01. 0.03]	0.04	[0.03. 0.06]	5.54	57450	0	100%
	Average participation date × Living alone × PPS	−0.01	[−0.01. 0.00]	−0.02	[−0.04. 0.00]	−2.14	226500	0.032	100%
	Average participation date × Duration × Living alone × PPS	−0.01	[−0.01. 0.00]	−0.03	[−0.04. 0.01]	−2.91	43200	0.004	100%
Covariates	*Education: less than bachelors degree*	0.29	[0.16. 0.47]	0.06	[0.02. 0.09]	3.49	3557	0	7.18%
	*Education: less than advanced degree*	0.12	[0.01. 0.25]	0.03	[0.00. 0.06]	2.00	3543	0.045	88.13%
	Age	−0.01	[−0.01.−0.01]	−0.8	[−0.11. −0.05]	−5.04	3549	0	100%

This table reports results of linear mixed models predicting loneliness as a function of Time, Patient Probability Score (PPS), and social isolation (see Extended Data Table 1), while controlling for demographic categories in 3588 participants with sufficient data. We report results that were significant at p < .05 for brevity. Consistent with Table 2, practically significant factors are bolded, statistically significant factors of undecided practical significance are italicized, and effects that were consistent with the null hypothesis are reported in plain text. For complete results, including factors whose p-values exceeded p =.05, see Supplementary Table 22. Results were evaluated using the following model: Loneliness ~ Gender + Education + Ethnicity + Racial identity + Setting + Age + Time between*Time within*Living Alone*PPS + (1 + Time within | SUBJECT_NUMBER).

**Extended Data Table 3 |:** Psychological distress as a function of social distancing

	Term	Coefficient	95% CI	Coefficient (stand.)	95% CI (stand.)	t-statistic	df	p.value	% in ROPE
Main effects	**(Intercept)**	6.11	[5.94, 6.28]	**0.00**	**[0.00**, 0.00]	69.88	3485	**0.000**	0%
	**PPS**	0.95	[0.90, 1.00]	0.52	[0.49, 0.54]	38.30	3498	**0.000**	0%
	*Average Social Distancing Stress (Social Distancing Stress between)*	0.50	[0.44, 0.55]	0.30	[0.27, 0.33]	19.06	3585	**0.000**	4%
	Social distancing stress within	0.19	[0.17, 0.21]	0.14	[0.12, 0.15]	17.54	2259	**0.000**	100%
	Average participation date	−0.10	[−0.15, −0.06]	−0.06	[−0.09, −0.03]	−4.40	3600	**0.000**	100%
	Average Social Distancing (Social Distancing between)	0.14	[ 0.06, 0.22]	0.05	[ 0.02, 0.08]	3.53	3578	**0.000**	100%
	Time with others within	0.07	[ 0.05, 0.09]	0.05	[ 0.04, 0.07]	6.45	1910	**0.000**	100%
	Duration	−0.03	[−0.06, −0.01]	−0.03	[−0.05, −0.01]	−2.61	2706	0.009	100%
	Social distancing within	0.04	[0.00, 0.07]	0.02	[0.00, 0.04]	2.10	1978	0.036	100%
Interactions	Average participation date × Duration	−0.03	[−0.04, −0.02]	−0.05	[−0.08, −0.03]	−5.10	2610	**0.000**	100%
	Social Distancing Stress within × Average Social Distancing Stress	0.04	[0.03, 0.05]	0.05	[ 0.04, 0.07]	6.94	2670	**0.000**	100%
	Duration × Average Social Distancing	0.04	[0.02, 0.06]	0.04	[0.02, 0.06]	4.21	2879	**0.000**	100%
	Average Social Distancing × PPS	0.05	[0.02, 0.08]	0.04	[0.01, 0.07]	3.03	3517	0.002	100%
	Average participation date × PPS	−0.03	[−0.05, 0.01]	−0.04	[−0.07, −0.01]	−2.88	3607	0.004	100%
	Duration × PPS	−0.02	[−0.04, −0.01]	−0.04	[−0.06, −0.02]	−4.41	2700	**0.000**	100%
	Average participation date × Duration × PPS × Average Social Distancing Stress	**0.00**	[0.00, 0.01]	0.03	[ 0.00, 0.06]	2.19	2624	0.029	100%
	Average participation date × Duration × Average Social Distancing	−0.01	[−0.02, 0.00]	−0.02	[−0.04, 0.00]	−2.20	2701	0.028	100%
	Duration × PPS × Social distancing stress within	0.01	[0.00, 0.01]	0.02	[ 0.00, 0.03]	1.97	19580	0.049	100%
	Duration × Social Distancing Stress within	−0.02	[−0.03,−0.01]	−0.02	[−0.04, −0.01]	−2.98	19170	0.003	100%
	Duration × Time with others within	0.01	[0.00, 0.03]	0.02	[0.00, 0.04]	2.26	9811	0.024	100%
	PPS × Time with others within	0.01	[0.00, 0.02]	0.02	[0.00, 0.04]	2.41	1902	0.016	100%
Covariates	**Education: Less than Bachelors**	0.82	[0.52, 1.13]	0.07	[0.04, 0.10]	5.30	3600	**0.000**	1%
	**Education: Less than Associates**	1.12	[0.50, 1.69]	0.05	[0.02, 0.07]	3.65	3684	**0.000**	2%
	Age	−0.04	[−0.05, −0.04]	−0.16	[−0.18, −0.13]	−11.85	3442	**0.000**	100%

This table reports results of linear mixed models predicting psychological distress as a function of Social Distancing, Time, and Patient Probability Score (PPS), while controlling for demographic categories in 3593 participants with sufficient data. Social distancing was operationalized through three self-reported social distancing measures (Distancing magnitude = ‘How much have you been social distancing?’; Distancing stress = ‘How stressful has it been for you to maintain social distancing?’; Time with others = ‘How much has your time with other people changed compared to how you acted before the COVID-19 outbreak?’). Consistent with Table 2, practically significant factors are bolded, statistically significant factors of undecided practical significance are italicized, and effects that were consistent with the null hypothesis are reported in plain text. For complete results, including factors whose p-values exceeded p =.05, see Supplementary Table 23. Results were evaluated using the following model: distress ~ Gender + Education + Ethnicity + Racial identity + Setting + Age + Time between*Time within*Social distancing within*Social Distancing between*PPS + Time between*Time within *Social Distancing Stress within*Social Distancing Stress between*PPS+ Time between*Time within *Time with others within*Time with others between*PPS+ (1 + Time within + Social distancing within+Social Distancing Stress within+Time with others within| SUBJECT_NUMBER).

**Extended Data Table 4 |:** Loneliness as a function of social distancing

	Term	Coefficient	95% CI	Coefficient (stand.)	95% CI (stand.)	t-statistic	df	p.value	% in ROPE
Main effects	**(Intercept)**	5.41	[5.32, 5.49]	0.00	[0.00, 0.00]	122.84	3545	0.000	0%
	**Average Social distancing stress**	0.25	[0.23, 0.28]	0.34	[0.31, 0.38]	19.8	3549	0.000	0.02%
	**PPS**	0.27	[0.24, 0.29]	0.32	[0.29, 0.35]	21.49	3544	0.000	0%
	Social distancing stress within	0.09	[0.07, 0.11]	0.14	[0.11, 0.17]	9.08	2226	0.000	100%
	Time with others within	0.06	[0.04, 0.08]	0.10	[0.06, 0.14]	4.79	2138	0.000	100%
	Average Time with others	0.11	[0.07, 0.14]	0.11	[0.08, 0.15]	6.29	3553	0.000	100%
	Social distancing within	0.05	[0.01, 0.08]	0.05	[0.02, 0.08]	2.84	2183	0.005	100%
Interactions	Average Social distancing × PPS	0.03	[0.01, 0.04]	0.04	[0.02, 0.07]	3.10	3535	0.002	100%
	Duration × Social distancing stress within	−0.01	[−0.02, −0.01]	−0.04	[−0.05, −0.04]	−17.15	284600	0.000	100%
	Duration × Average Social distancing	0.01	[0.00, 0.03]	0.03	[0.00, 0.06]	2.11	2226	0.035	100%
	Social distancing stress within × Average Social distancing stress	0.01	[0.00, 0.02]	0.03	[0.00, 0.05]	2.27	2319	0.024	100%
	Duration × Social distancing within × PPS	0.00	[−0.01, 0.00]	−0.01	[−0.02, −0.01]	−4.32	282200	0.000	100%
	Duration × Social distancing within × Average Social distancing	0.00	[0.00, 0.00]	−0.01	[−0.02, 0.00]	−2.79	284300	0.005	100%
	Duration × Social distancing stress within × Average Social distancing stress	0.00	[0.00, 0.00]	−0.02	[−0.02, −0.01]	−6.28	283200	0.000	100%
	Average participation date × Duration × Time with others within	0.00	[0.00, 0.00]	0.01	[0.00, 0.02]	3.32	282300	0.001	100%
	Average participation date × Duration × Social distancing stress within × PPS	0.00	[0.00, 0.00]	−0.01	[−0.01, 0.00]	−2.40	284500	0.016	100%
	Average participation date × Duration × Time with others within × PPS	0.00	[0.00, 0.00]	0.01	[0.00, 0.01]	1.97	282100	0.048	100%
	Duration × PPS × Time with others within × Average Time with others	0.00	[0.00, 0.00]	−0.01	[−0.02, −0.01]	−4.08	282900	0.000	100%
	Average participation date × Duration × PPS × Social distancing stress within × Average Social distancing stress	0.00	[0.00, 0.00]	−0.01	[−0.02, 0.00]	−3.06	284000	0.002	100%
	Average participation date × Duration × Social distancing within	0.00	[0.00, 0.00]	0.01	[0.00, 0.01]	2.52	282400	0.012	100%
	Duration × PPS × Social distancing stress within × Average Social distancing stress	0.00	[0.00, 0.00]	0.01	[0.00, 0.01]	2.28	282700	0.023	100%
	Duration × PPS × Social distancing stress within	0.00	[0.00, 0.00]	0.01	[0.00, 0.01]	2.23	284600	0.026	100%
	Duration × PPS × Time with others within	0.00	[0.00, 0.00]	−0.01	[−0.02, −0.01]	−3.53	282200	0.000	100%
Covariates	*Education: Less than Bachelors*	0.29	[0.14, 0.46]	0.06	[0.03, 0.08]	3.75	3550	0.000	10.9%
	*Racial identity: AAPI*	0.41	[0.11, 0.69]	0.04	[0.01, 0.07]	2.88	3539	0.004	8.65%
	*Gender: Man*	0.27	[0.15, 0.42]	0.06	[0.03, 0.08]	4.01	3539	0.000	11.75%
	*Education: Less than advanced*	0.13	[0.2, 0.24]	0.03	[0.01, 0.06]	2.35	3532	0.019	90.28%
	Age	−0.01	[−0.01, 0.00]	−0.05	[−0.08, −0.02]	−3.13	3531	0.002	100%

This table reports results of linear mixed models predicting loneliness as a function of Social Distancing, Time, and Patient Probability Score (PPS), while controlling for demographic categories in 3588 participants with sufficient data. Factors were identical to Extended Data Table 3, and we use the same reporting conventions: practically significant factors are bolded, statistically significant factors of undecided practical significance are italicized, and effects that were consistent with the null hypothesis are reported in plain text. Complete results, including those whose statistical significance exceeded p = .05 are reported in Supplementary Table 24. Results were evaluated using the following model: loneliness ~ Gender + Education + Ethnicity + Racial identity + Setting + Age + Time between*Time within*Social distancing within*Social Distancing between-subjects*PPS + Time between*Time within*Social Distancing Stress over time*Social Distancing Stress between-subjects*PPS+ Time between*Time within*Time with others over time*Time with others between*PPS+ (1 + Time within + Social distancing within+Social Distancing Stress within +Time with others within| SUBJECT_NUMBER).

**Extended Data Table 5 |:** Longitudinal model of loneliness as a function of regional social distancing

	Parameter	Coefficient	95% CI	Coefficient (stand.)	95% CI (stand.)	t-statistic	df	p.value	% in ROPE
Main effects	**(Intercept)**	5.48	[5.34, 5.58]	0.00	[ 0.00, 0.00]	107.63	3386	0.000	0.00%
	**PPS**	0.34	[0.32, 0.38]	0.41	[ 0.38, 0.45]	24.37	3385	0.000	0.00%
Interactions	**Duration × Regional distancing within × Average Regional distancing (Regional distancing between)**	0.64	[0.36, 0.91]	0.02	[0.01, 0.02]	4.63	267600	0.000	0.08%
	**Duration × Regional distancing within × Average Regional distancing × PPS**	0.39	[0.27, 0.51]	0.02	[ 0.02, 0.03]	6.46	267700	0.000	0.06%
	*Average regional distancing × PPS*	0.19	[0.00, 0.34]	0.03	[0.00, 0.07]	2.04	3386	0.041	57.37%
	*Average participation date × Duration × Regional distancing within × Average Regional distancing*	0.12	[ 0.02, 0.22]	0.01	[0.00, 0.01]	2.42	274200	0.016	93.35%
	Duration × Regional distancing within	0.13	[0.08,0.17]	0.03	[ 0.02, 0.04]	5.97	264000	0.000	100%
	Average participation date × PPS	0.01	[ 0.00, 0.02]	0.03	[0.00, 0.07]	1.99	3382	0.047	100%
	Average participation date × Duration × Regional distancing within × PPS	0.03	[ 0.02, 0.03]	0.03	[0.02, 0.03]	7.14	267700	0.000	100%
	Average participation date × Duration × Regional distancing within	0.02	[ 0.00, 0.03]	0.01	[0.00, 0.01]	4.63	267600	0.000	100%
	Duration × Regional distancing within × PPS	−0.02	[−0.04, - 0.01]	−0.01	[−0.02, 0.00]	−2.47	265800	0.014	100%
Covariates	*Education: Less than Bachelors*	0.28	[0.13, 0.44]	0.05	[0.02, 0.09]	3.19	3386	0.001	16.27%
	*Setting: Urban*	0.14	[ 0.02, 0.27]	0.04	[ 0.00, 0.07]	2.13	3378	0.033	74.91%
	Age	−0.01	[−0.01, 0.00]	−0.07	[−0.10, −0.04]	−4.16	3375	0.000	100%

This table reports results of linear mixed models predicting loneliness as a function of Regional Distancing (based on regional cell phone mobility data^51^ within US participants; higher values = less mobility / more distancing), Time, and Patient Probability Score (PPS), while controlling for demographic categories in 3415 participants with sufficient data. We use the same reporting conventions as Table 2 and Extended Data Tables 1–4: Practically significant factors are bolded, statistically significant factors of undecided practical significance are italicized, and effects that were consistent with the null hypothesis are reported in plain text. Complete results including non-significant factors (p > 0.05) are reported in Supplementary Table 25. Results were evaluated using the following model: loneliness ~ Gender + Education + Ethnicity + Racial identity + Setting + Age + Time between*Time within* Regional distancing within* Regional distancing between*PPS + (1 + Time within + Regional distancing within| SUBJECT_NUMBER).

**Extended Data Table 6 |:** Mediation models and moderation by Living Alone and PPS

	Model	Statistic	Path *a*	Path *b*	Path *c’*	Path *c*	Path a*b
Across participants (i.e., single level mediation)	No moderators	Coeff	0.35	1.09	0.34	0.73	0.39
		CI	[0.34, 0.38]	[1.01, 1.16]	[0.29, 0.40]	[0.67, 0.79]	[0.35, 0.42]
		P	<0.001	<.001	<.001	<.001	<.001
	With Moderators: Controlling for Moderators	Coeff	0.29	0.67	0.25	0.44	0.19
		CI	[0.27, 0.31]	[0.60, 0.74]	[0.2., 0.3]	[0.4, 0.49]	[0.17, 0.22]
		P	<0.001	<0.001	< 0.001	<0.001	<0.001
	With Moderators: Moderation by Living Alone	Coeff	0.14	−0.02	−0.04	0.04	−0.09
		CI	[0.09, 0.19]	[−0.2, 0.15]	[−0.17, 0.08]	[−0.07, 0.16]	[−0.16, −0.00]
		P	<0.001	**n.s.**	**n.s.**	**n.s.**	0.042
	With Moderators: Moderation by PPS	Coeff	0.00	0.07	−0.01	0.01	−0.08
		CI	[−0,01, 0.01]	[0.04, 0.11]	[−0.04, 0.01]	[−0.01, 0.03]	[−0.14, −0.03]
		P	**n.s.**	<0.001	**n.s.**	**n.s.**	0.008
	With Moderators: Moderation by PPS × Living Alone	Coeff	0.01	0.02	−0.01	0.01	0.00
		CI	[−0.01, 0.03]	[−0.06, 0.09]	[−0.07, 0.04]	[−0.04, 0.06]	[−0.04, 0.04]
		P	**n.s.**	**n.s.**	**n.s.**	**n.s.**	**n.s.**
Within participants (i.e., multilevel mediation)	No moderators	Coeff	0.11	0.54	0.14	0.21	0.07
		CI	[0.10, 0.12]	[0.51, 0.58]	[0.12, 0.16]	[0.19, 0.23]	[0.06, 0.08]
		P	<0.001	<0.001	< 0.001	<0.001	< 0.001
	With Moderators: First level	Coeff	0.10	0.50	0.15	0.20	0.05
		CI	[0.09, 0.11]	[0.47, 0.54]	[0.12, 0.17]	[0.18, 0.22]	[0.05, 0.06]
		P	<0.001	<0.001	<0.001	<0.001	< 0.001
	With Moderators: Second Level moderation by Living Alone	Coeff	0.04	0.09	0.02	0.05	0.001
		CI	[0.02, 0.06]	[0.01, 0.15]	[−0.03, 0.06]	[0.01, 0.10]	[−0.01, 0.01]
		P	0.001	.025	**n.s.**	0.026	**n.s.**
	With Moderators: Second Level moderation by PPS	Coeff	**0.00**	0.04	0.01	0.01	−0.001
		CI	[0, 0.01]	[0.02, 0.05]	[0.00, 0. 02]	[0, 0.021	[−0.003, 0.00]
		P	**n.s.**	<0.001	**n.s.**	0.013	0.04
	With Moderators: Second Level moderation by PPS × Living Alone	Coeff	**0.00**	0.01	−0.01	−0.01	0.002
		CI	[−0.01, 0.011	[−0.02, 0.05]	[−0.03, 0.00]	[−0.04, 0.01]	[−0.001, 0.005]
		P	**n.s.**	**n.s.**	**n.s.**	**n.s.**	**n.s.**

This table reports results of mediation models evaluating whether loneliness mediates associations between social distancing-related stress (X) and distress (Y). We used single level mediation to evaluated associations across participants (that is, one value per participant) and multilevel mediation to evaluate dynamic associations within participants over time. For each approach, Path a evaluates associations between social distancing-related stress and loneliness. Path b evaluates associations between loneliness and distress while controlling for distancing-related stress. Path c evaluates associations between social distancing-related stress and distress without controlling for loneliness, while c‵ evaluates relationships when controlling for loneliness. Path a*b evaluates the overall mediation effect, or indirect pathway through loneliness. All factors except Psychological Distress were centered in all models. For each mediation approach, we evaluated two mediation models: one that evaluated associations regardless of Living Alone or Patient Probability Score (PPS), and a second that evaluated whether mediation effects were moderated by Living Alone and/or PPS, thus generating effects while controlling for Living Alone and PPS and tests of which paths varied as a function of Living Alone and/or PPS. Sample sizes were as follows: 3564 participants in single level mediation without moderators (top row); 3543 participants in single level mediation with moderators (second rows); 3592 participants in multilevel mediation without moderators (third row); and 3584 participants in multilevel mediation with moderators (bottom rows). See Methods for full details of mediation analysis significance testing.

## Supplementary Material

Supplementary Material

## Figures and Tables

**Table 1 |:** PPS as a function of demographics

Demographic variable	Level	*N* (%)	PPS:Mean (s.d.)	Relationship with PPS
	Woman	2,894 (80.48)	0.57 (0.21)	
		
	Man	595 (16.55)	0.49 (0.22)	
		
Gender	Non-conforming	43 (1.2)	0.77 (0.16)	F(5, 3,590) = 29.09, *P* < 0.001
		
	Trans	9 (0.25)	0.73 (0.21)	
		
	Other	13 (0.36)	0.84 (0.07)	
		
	Missing	42 (1.17)	0.55 (0.23)	

	White	3,216 (89.43)	0.57 (0.22)	
		
	African American/Black	121 (3.36)	0.48 (0.21)	
		
	Asian American/Pacific Islander	109 (3.03)	0.45 (0.21)	
		
Racial Identity	American Indian/Native American	34 (0.95)	0.55 (0.17)	F(6, 3,589) = 8.75, *P* < 0.001
		
	Multiple	63 (1.75)	0.57 (0.22)	
		
	Unknown	13 (0.36)	0.57 (0.16)	
		
	Missing	40 (1.11)	0.56 (0.21)	

	Not Latino	3,243 (90.18)	0.56 (0.22)	
		
Ethnicity	Latino	200 (5.56)	0.57 (0.21)	F(3, 3,592) = 0.068, *P* = 0.977
		
Unknown	32 (0.89)	0.55 (0.21)
		
	Missing	121 (3.36)	0.56 (0.22)	

	Suburban	1,889 (52.53)	0.56 (0.22)	
		
Setting	Urban	1,173 (32.62)	0.53 (0.21)	F(3, 3,592) = 30.22, *P* < 0.001
		
	Rural	518 (14.40)	0.64 (0.20)	
		
	Missing	16 (0.44)	0.46 (0.19)	

	Advanced professional degree	1,875 (52.14)	0.51 (0.20)	
		
	Less than advanced professional degree	1,129 (31.4)	0.59 (0.22)	
		
Education	Less than bachelor’s degree	482 (13.4)	0.66 (0.21)	F(4, 3,591) = 65.91, *P* < 0.001
		
	Less than associate degree	99 (2.75)	0.69 (0.21)	
		
	Missing	11 (0.31)	0.55 (0.17)	

Age	[18, 87]	3,596 (100)	0.56 (0.22)	B = −0.002 (0.00), **t** = −7.17, *P* < 0.001

Household size	[1.00, 10.00]	3,581 (100)	0.56 (0.22)	B = −0.001 (.003), **t** = −0.45, *P* = 0.654

	Living with others	2,813 (78.23)	0.56 (0.22)	
		
Household status	Living alone	768 (21.36)	0.58 (0.22)	F(2, 3,593) = 3.391, *P* = 0.0338
		
	Missing	15 (0.42)	0.61 (0.20)	

This table presents mean PPS as a function of demographic categories across participants who completed baseline questionnaires (*n* = 3,596). We used the function ‘summary_factorlist’ in the R package ‘finalfit’^52^ to conduct separate linear models evaluating whether PPS varies significantly as a function of each demographic variable and to generate the results table. *P* values reflect results of the omnibus *F* test for each demographic category (two-tailed, no multiplicity correction). We included covariates for each category in longitudinal models.

**Table 2 |:** Main model: longitudinal associations with psychological distress

	Term	Coefficient^a^	95% CI^b^	Coefficient (stand)^c^	95% CI (stand)^c^	t-statistic^a^	d.f.^a^	*P* value^a^	% in ROPE^b^
	(Intercept)^†^	6.29	[6.12, 6.46]	0.00	[0, 0]	75.04	3,477	0.000	0
	
	Average loneliness (Loneliness between)^†^	0.95	[0.89, 1.00]	0.42	[0.40, 0.45]	32.60	3,551	0.000	0
	
	Patient probability score (PPS)^†^	0.77	[0.72, 0.82]	0.42	[0.39, 0.44]	31.10	3,471	0.000	0
	
Main effects	Loneliness over time (Loneliness within)^†^	0.57	[0.53, 0.60]	0.26	[0.24, 0.28]	30.94	2,065	0.000	0
	
	Average participation date (Time between)^§^	−0.07	[−0.11, −0.03]	−0.04	[−0.07, −0.02]	−3.49	3,606	0.001	100
	
	Duration (Time within)^§^	−0.03	[−0.05, −0.01]	−0.02	[−0.04, −0.01]	−2.80	2,636	0.005	100

	PPS × Average participation date^§^	−0.04	[−0.05, −0.01]	−0.05	[−0.07, −0.02]	−3.47	3,611	0.001	100
	
	PPS × Duration^§^	−0.03	[−0.03, −0.01]	−0.05	[−0.07, −0.03]	−5.08	2,514	0.000	100
	
Interactions	Loneliness between × Loneliness within^§^	0.06	[0.03, 0.08]	0.04	[0.02, 0.05]	4.53	2,640	0.000	100
	
	Average participation date × Duration^§^	−0.02	[−0.03, −0.01]	−0.04	[−0.06, −0.02]	−4.68	2,597	0.000	100
	
	Duration × Loneliness between^§^	0.03	[0.02, 0.05]	0.04	[0.02, 0.06]	3.99	2,568	0.000	100

	PPS × Loneliness between^§^	0.03	[0.01, 0.06]	0.03	[0.01, 0.05]	2.69	3,488	0.007	100

	PPS × Loneliness within^§^	0.02	[0.01, 0.04]	0.02	[0.01, 0.04]	2.69	2,017	0.007	100

	Education: less than associate degree^‡^	0.84	[0.28, 1.39]	0.03	[0.01, 0.06]	2.91	3,698	0.004	8.38
	
	Education: less than bachelor’s degree^‡^	0.57	[0.28, 0.85]	0.05	[0.02, 0.07]	3.84	3,610	0.000	19.98
	
	Racial identity: African American^‡^	−0.62	[−1.12, −0.11]	−0.03	[−0.05, −0.01]	−2.40	3,685	0.017	26.24
	
Covariates	Racial identity: Asian American/Pacific Islander^‡^	−0.53	[−1.05, 0]	−0.02	[−0.05, 0]	−1.98	3,479	0.048	39.75
	
	Gender: Man^‡^	−0.48	[−0.72, −0.23]	−0.04	[−0.07, −0.02]	−3.89	3,455	0.000	38.43
	
	Ethnicity: Latino^‡^	0.46	[0.05, 0.88]	0.03	[0, 0.05]	2.17	3,508	0.030	48.65
	
	Age^§^	–0.04	[−0.04, −0.03]	−0.13	[−0.16, −0.11]	−11.30	3,427	0.000	100

This table reports results of linear mixed models predicting psychological distress (measured by the Kessler-5^40^) as a function of time, loneliness and PPS, while controlling for demographic categories in 3,585 participants with sufficient data. With the exception of covariates, all predictors were mean-centered to facilitate interpretation of coefficients and interactions. The table reports terms whose associations were statistically significant based on frequentist models (*P* < 0.05); for all factors, see Supplementary Table 20. Within each category, factors are sorted on the basis of practical significance (that is, percentage in ROPE) and by absolute value of the standardized coefficient. Effects that are both statistically and practically significant (<2.5% of posterior estimates in ROPE) are marked with

† effects that are statistically significant but of uncertain practical significance (between 2.5% and 95% of posterior estimates in ROPE) are marked with

‡ and effects that are consistent with the null hypothesis (>97.5% in ROPE) are marked with §.

a Frequentist results were evaluated using the function ‘lmer’ from the R package ‘lme4’^53^ using the following model: distress ~ Gender + Education + Ethnicity + Racial Identity + Setting + Age + PPS*Time between + PPS*Time within + PPS*Loneliness between*Loneliness within +Loneliness between*Loneliness within*Time between*Time within + (1 + Interval + Loneliness within | SUBJECT_NUMBER)

b Bayesian results were evaluated using the function ’brms’ from the R package ‘brms’^55^ using the same model specified for ‘lme4.’ All coefficients were modeled with normal priors (set_prior(‘normal(0,2.5)’, class = ‘b’)) and we included 1,000 warm-up samples and 4,000 iterations. Posterior estimates, including 95% confidence intervals, and the ROPE were obtained using the ‘describe_posterior’ function from the package BayesTestR^56^ and interpreted as in ref. 57 to evaluate practical significance. The ROPE was defined as [–0.45, 0.45].

c We evaluated pseudo-standardized coefficients and confidence intervals using the package ‘effectsize.’^54^

## Data Availability

Participant-level data used for these analyses are available at https://osf.io/e7jrd/ (ref. 62) in the file ‘final_socmeasures_052824_n3605. csv’. The complete dataset can be found at https://dataverse.harvard.edu/dataset.xhtml?persistentId=doi:10.7910/DVN/L4LRM2 (ref. 63), which includes data obtained from the NIEHS COVID-19 Pandemic Vulnerability Index Dashboard.^51^ All data are publicly available without restrictions.
