# Supplementary material for: Dynamic effects of psychiatric vulnerability, loneliness and isolation on distress during the first year of the COVID-19 pandemic
Source: Nat Ment Health. Author manuscript; Available in PMC 2025 Mar 25. (PMC11934869; doi:10.1038/s44220-024-00371-6)
Supplement: Supplementary Material [file NIHMS2056759-supplement-Supplementary_Material.pdf]

nature mental health

Supplementary information https://doi.org/10.1038/s44220-024-00371-6

Dynamic effects of psychiatric vulnerability, loneliness and isolation on distress during the first year of the COVID-19 pandemic

In the format provided by the authors and unedited

Table of Contents

SECTION	Page
Supplementary Methods	2
Supplementary Results	3
Supplementary References	7
Figure S1. Receiver Operating Characteristic for relationship between PPS and self-reported mental health treatment history	8
Figure S2. Self-reported clinical history in participants with high PPS values.	9
Figure S3. Model validation	10
Figure S4. Likelihood of repeat response as a function of patient probability score, social isolation, and sociodemographic categories	11
Figure S5. Psychological distress as a function of pandemic vulnerability, PPS, and Duration	12
Figure S6. Associations between loneliness, PPS, and other mental health outcome measures	13
Figure S7. Loneliness and relationship quality	14
Figure S8. Associations between self-reported social-distancing, psychological distress, and loneliness	15
Table S1. Pair-wise correlations between mental health outcome measures	16
Table S2. Pair-wise correlations between social distancing measures	17
Table S3. Longitudinal model of distress with age and gender interactions	18
Table S4. Main longitudinal model controlling for changes in pandemic vulnerability	20
Table S5. Longitudinal model of mental health as operationalized by PHQ2	23
Table S6. Longitudinal model of mental health as operationalized by DSM-XC general mental health factor score	25
Table S7. Longitudinal model of clinically-significant mental health	27
Table S8. Longitudinal model of distress as a function of household size	28
Table S9. Longitudinal model of loneliness as a function of household size	30
Table S10. Longitudinal model of distress as a function of relationship quality	32
Table S11. Longitudinal model of loneliness as a function of relationship quality	34
Table S12. Longitudinal model of distress as a function of social and emotional support	35
Table S13. Longitudinal model of loneliness as a function of social and emotional support	36
Table S14. Longitudinal model of distress as a function of social distancing	37
Table S15. Longitudinal model of loneliness as a function of social distancing	39
Table S16. Longitudinal model of distress as a function of social distancing, age, and gender	41
Table S17. Longitudinal model of loneliness as a function of social distancing, age, and gender	43
Table S18. Longitudinal model of distress as a function of community-based social distancing	46
Table S19. Reverse mediation models	47
Table S20. Longitudinal associations with psychological distress: Complete results.[v]	48
Table S21. Longitudinal model of distress as a function of loneliness and social isolation: Complete results.	50
Table S22. Longitudinal model of loneliness as function of social isolation: Complete results.	53
Table S23. Psychological distress as a function of social distancing: Complete results.	55
Table S24. Loneliness as a function of social distancing: Complete results.	58
Table S25. Longitudinal model of loneliness as a function of regional social distancing: Complete results.	62

Supplementary Methods

Patient probability score validation. PPS scores were originally validated against self-reported treatment history in an initial "lockdown cohort" that enrolled between April 4 and May 15, 2020 (n = 1992)[18]. Comparing PPS with self-reported treatment history (i.e., whether an individual reported having undergone "mental health hospitalization", "treatment for alcohol and/or drug abuse", and/or "medication for a mental health condition" on a clinical history questionnaire at baseline) yielded an area-under-the-curve (AUC) of the receiver operating characteristic (ROC) of 0.87. To further validate this measure in the present dataset (n = 3596 with self-reported treatment history and sufficient data to calculate PPS), we used the program "auc_roc" in R's "mltools" package[62] and the same definition of self-reported treatment history. AUC for the complete dataset, excluding the NIH participants used in the training set, was 0.86, and the ROC curve is presented in Supplementary Figure S1.

As PPS is a dimensional measure reflecting psychiatric vulnerability, i.e., one's likelihood of having ever undergone treatment for mental health, it purposely does not reflect an individual's likelihood of having a specific diagnosis. Yet insight is possible in terms of the association between PPS and specific self-reported psychiatric diagnoses. While our previous manuscript reports the exact loadings between individual survey items and PPS score[18], here we evaluated free responses to self-reported treatment history from our clinical history questionnaire. In this item, participants reported whether they had ever undergone medical hospitalization, mental health hospitalization, treatment for alcohol and/or drug abuse, mental health counseling, medication for a mental health condition or none of the above. (As mentioned above, three of these items were used to validate PPS.) Participants were then asked "Please describe reason(s)". We extracted free responses to this item for participants in the top quartile of PPS scores (n = 899; range: [0.75, 0.97]) and used natural language processing to evaluate the frequency of distinct terms (after converting to lower case, removing numbers and white spaces, punctuation, and unimportant words based on the "Smart" stopwords in the R package tm[63,64]). Supplementary Figure S2 presents the frequency of terms in a histogram (top ten words) and word cloud, where the word size reflects frequency (word cloud generated with the R package wordcloud2[65]. As illustrated in Supplementary Figure S2, the top term was "depression" (538 occurrences), followed by "anxiety" (478 occurrences). Ignoring general terms ("disorder", "mental", "health", "counseling", "medication"), the next frequent words were "ptsd" (147 occurrences), "hospitalization", and "bipolar" (127 occurrences). We also observed mentions of "suicide" / "suicidal" (117 occurrences) and "adhd" (57 occurrences). Thus, based on free response, we may conclude that most participants report having been treated for mood disorders, PTSD, suicidality, and ADHD.

Outcome measure selection. We computed pair-wise correlations between mental health outcomes across intervals (Table S1). All correlations exceeded 0.79, and thus we concluded that the outcomes measured similar constructs. We did the same for social distancing measures (Table S2) and observed much weaker associations, thus supporting the idea that outcomes measure different aspects of distancing. We therefore separately analyzed associations with each measure of social distancing.

Model validation We inspected q-q plots and residuals of the main model using Kessler-5 as the dependent variable. Figure S3 presents q-q plot and residuals of the overall model as well as with respect to each of the main effects. Residuals were determined to be normally distributed.

Interactions with Age and Gender. Our main manuscript reports results while controlling for demographic factors including age, racial identity, ethnicity, education, and setting. Because our sample was not balanced, we used factor coding and thus the intercept and slopes reflect

results in the dominant subgroup. In supplementary analyses, we mean-centered all factors and formally tested for interactions with age and gender.

Supplementary Results

Repeat responders. Of the 3655 individuals who originally enrolled in the study, 3149 provided responses across multiple intervals, while 451 individuals responded only at baseline. We used logistic regression to evaluate whether repeat responders differed from those who replied at baseline only in terms of demographics, PPS, or social isolation. There was no impact of Gender or Living alone on one's likelihood of replying across multiple intervals (all p's > 0.1; see Supplementary Figure S4). Age was positively associated with likelihood of responding (B = 0.016, p < .001, Odds Ratio = 1.02), and we observed a significant effect of Education, such that those with less than a bachelor's degree were least likely to reply across multiple intervals (B = -0.722, p < .001, OR = 0.49). Associations between repeat responder status and PPS, racial identity, ethnicity, and setting did not survive our conservative statistical threshold; see Supplementary Figure S4 for complete results.

Influence of Age and Gender on associations with distress. Table S3 evaluates whether associations between Distress, Time, PPS, and Loneliness vary as a function of Age or Gender. Consistent with analyses reported in the main manuscript (Table 2), we observed a main effect of Age, such that distress was lower on average for older adults, and we also observed significant interactions between Age and Duration (B = 0.004, p < .001, *b* = 0.04), and Gender x Duration (B = -0.07, p < .001, *b* = -0.05). However, Bayesian models indicated that all of these effects were consistent with the null hypothesis (see Table S3). For additional interactions that did not survive our conservative threshold and were also consistent with the null hypothesis based on Bayesian models, see Table S3.

Relationships between distress, loneliness, and likelihood of psychiatric diagnosis when accounting for Pandemic Vulnerability Index (PVI). Associations between time, PPS, loneliness, and psychological distress reported in the main manuscript (Table 2) remained significant when we controlled for fluctuations in PVI within and across US participants (Table S4). There were no main effects of PVI (all p's > 0. 2). The only practically significant association with PVI was a three-way interaction between Duration, Average PVI, and PPS (B = -1.47, p < .001; *b* = -0.11), such that individuals with high PPS scores in areas with high pandemic vulnerability reported the largest reductions in psychological distress across time (see Figure S5). For complete results, see Table S4.

Association between loneliness, psychiatric vulnerability, and other mental health outcomes. Consistent with the high pair-wise correlations between our three mental health outcome measures (Table S1), associations between time, PPS, loneliness and depression or anxiety, as measured by PHQ-2[40] (Table S5), or general psychiatric health, as measured by a factor score derived from the DSM-XC[39,41] (Table S6), were largely comparable to associations with psychological distress, as measured by the Kessler-5[38] and reported in the main manuscript (Table 2). Figure S6 depicts practically significant associations with PPS and loneliness as a function of outcome measure for comparison with Figure 2 in the main manuscript.. The only substantial differences we observed when we used PHQ2 scores or DSM XC scores as mental health outcomes rather than Kessler-5 (i.e. cases when findings were practically significant in one model but not another) were that participants with less than a Bachelor's degree had higher PHQ2 scores and DSM-XC scores than those with advanced degrees (see Tables S5 and S6)

and participants with less than an Associate's Degree had higher DSM-XC scores than those with advanced degrees (Table S6). For complete results, see Tables S5 and S6.

Predicting clinically-significant mental health. While our main model treats distress as a continuous outcome, we were also interested in whether predictors were associated with clinically-significant responses to surveys at a given time point, as indicated by cutoff scores greater than 10 on the Kessler-5[38] and/or scores greater or equal to 3 on the PHQ-2[40]. Results of logistic regressions (Table S7) were largely consistent with analyses that treated distress as a continuous outcome. The only substantial differences we observed (i.e. cases when statistical thresholds were less than $p < .001$ in one model and exceeded $p > .05$ in the other) were that we no longer observed interactions between PPS and Duration or within- and between-subjects loneliness. For complete results and standardized coefficients, see Table S7.

Social isolation and household size impact loneliness and mental health during the pandemic. Because loneliness was strongly associated with fluctuations in mental health during the pandemic, we further explored the contribution of social factors to loneliness and mental health. We focused on objective social isolation (Tables S8 and S9), relationship quality (Tables S10 and S11) and social and emotional support (Tables S12 and S13).

Social isolation was indexed both categorically (i.e., whether a respondent was currently living alone or with others) and continuously (i.e., household size). We report results of categorical analyses in the main manuscript and evaluate associations with household size in supplementary materials. Consistent with categorical analyses, household size was positively associated with distress across individuals (B = 0.30, $p < .001$, *b* = 0.08; see Table S8), such that people in larger households reported more distress than those living alone or with few others, although this effect was consistent with the null hypothesis based on Bayesian models. Again, variations in loneliness both within and across individuals still predicted distress when controlling for the number of people in the household, and both effects were practically significant based on Bayesian analyses. For complete results, see Table S8.

We also asked how objective social isolation impacts self-reported loneliness. Consistent with categorical findings reported in the main manuscript, household size was negatively associated with loneliness, such that those who lived in larger households reported less loneliness on average (B = -0.28, $p < .001$; *b* = -0.16) and effects were practically significant based on Bayesian models (see Table S9). Statistically significant interactions between Household Size, PPS, and Time were not sufficient to reject the null hypothesis; for complete results, see Table S9.

Impact of relationship quality and social support. Given that household size had different effects on distress and loneliness, we next asked whether mental health and loneliness were associated with relationship quality and/or support. Results are reported in Table S10. Individuals who reported more positive relationships between members of their family/household on average also reported less distress (B = -0.28(0.03), $p < .001$, *b* = -0.12), and distress was inversely related to relationship quality within individuals over time (B = -0.21(0.01), $p < .001$, *b* = -0.12). However, both effects were consistent with the null hypothesis based on Bayesian models (see Table S10). There were no interactions with relationship quality that survived our conservative statistical threshold. For complete results, see Table S10.

Relationship quality also impacted loneliness, such that individuals who reported more positive relationships on average reported less loneliness (B = -0.43, $p < .001$, *b* = -0.42; see Figure S7) and individuals reported less loneliness at intervals when they reported that relationship quality was more positive (B = -0.17, $p < .001$, *b* = -0.21). In contrast to analyses of distress, associations between average relationship quality and loneliness across individuals were practically significant, while associations within individuals over time were consistent with

the null hypothesis (Table S11). We observed several significant interactions between relationship quality, Duration, Average Date, and PPS, although Bayesian analyses indicated these associations were consistent with the null hypothesis; for complete results, see Table S11.

Finally, we evaluated associations with social and emotional support. We examined whether individual differences in emotional and/or social support (reported at the end of the study) were associated with differences in self-reported distress and/or loneliness during participation. Interestingly, when the two were included in the same model (Table S12), emotional support was negatively linked to distress (B = -0.05, p <.001, *b* = -0.09), whereas social support was positively associated with distress (B = 0.04, p < .001, *b* = 0.10). However, both had negative associations when we examined simple correlations, and Bayesian analyses suggested these results were consistent with the null hypothesis (Table S12). We did not observe any interactions between support and other factors (see Table S12).

In contrast to distress, average loneliness was negatively associated with both emotional support (B = -0.07, p < .001, *b* = -0.29) and social support (B = -0.04, p < .001, *b* = 0.23), such that individuals who reported more support experienced less loneliness across the pandemic. We also observed a significant interaction between Duration and emotional support (B = -0.01, p < .001, *b* = -0.08) such that those who reported higher emotional support reported stronger reductions in loneliness over time. However, Bayesian analyses indicated that these main effects and interactions were not sufficient to reject the null hypothesis (Table S13). For complete results, including interactions that did not survive our stringent threshold, see Table S13.

Associations between distress, loneliness, and social distancing. Our main manuscript incorporates all self-reported social distancing measures in a single model, thus evaluating whether each measure was associated with distress and/or loneliness while controlling for all other measures. We also measured associations with each measure independently. Individual differences in relationships with distress and loneliness for each measure are depicted in Figure S8.

Table S14 reports associations with distress as a function of social distancing measure. Distress was positively associated with time with others and stress related to social distancing, both within individuals over time and across individuals based on our conservative statistical threshold (all p's < .001), while we did not observe significant associations with the magnitude of social distancing on average or within individuals (all p's > 0.03). Bayesian analyses indicated that within-subjects fluctuations in each measure were not practically significant predictors of distress, and that results were in fact consistent with the null hypothesis. In contrast, Bayesian analyses suggested that practical significance of between-subjects variations varied across measures: Individual differences in stress associated with distancing were practically significant predictors of distress (0.13% in ROPE), whereas individual differences in time spent with others and magnitude of distancing were consistent with the null hypothesis (>97.5% in ROPE). Table S14 displays other factors that differed across models, however none of these were found to be practical predictors of distress, with the exception of PPS.

Table S15 reports associations with loneliness as a function of social distancing measure. For each measure, loneliness was positively associated with variations over time within individuals (all p's < .001), and we also observed associations between average loneliness and average time with others and stress related to distancing (all p's < .001); associations with average magnitude of distancing did not survive our statistical threshold (p = 0.025). Similar to distress, within-subjects effects were not practically significant based on Bayesian models. Bayesian models indicated that individual differences in stress associated with distancing and time with others were both practically significant predictors of loneliness, whereas individual differences in social distancing magnitude was consistent with the null

hypothesis. Table S15 displays other factors that differed across models based on frequentist statistics including interactions with social distancing, but all of these were consistent with the null hypothesis based on Bayesian models.

Table S16 reports associations between distress, all three self-reported distancing measures, age, and gender, complementing the main manuscript's analyses which treated demographic factors as covariates. The only interaction that survived our conservative statistical threshold was a Gender x Time between x Time with others within x Time with others between interaction (B = -0.01, p < .001, *b* = -0.04), suggesting that the relationships between time with others and distress varied as a function of gender and time of enrollment, as well as individual differences in self-reported time with others. We also evaluated associations between all three self-reported distancing measures, age, gender, and loneliness (Table S17). Although we did not observe any simple interactions, we observed several three- and four-way interactions with Age and Gender. Complete results are reported in Table S17.

Finally, we evaluated associations with community-based social distancing, based on regional estimates of mobility from cell phone data. Our main manuscript reports associations between regional distancing and loneliness (Extended Data Table 5). Associations with distress are reported in Table S18. We did not observe any main effects of regional distancing on distress (all p's > 0.06). We did observe significant interactions between within-subjects variations in regional distancing and Duration (B = 0.35, p < 001, *b* = 0.03), such that distress increased over time when regional distancing was higher, and between regional distancing, Duration, and Average Date (B = -0.17, p < .001, *b* = -0.04); however both effects were consistent with the null hypothesis. For complete results, see Table S18.

Reverse mediation models. The mediation model tested in our main manuscript evaluates whether associations between distancing-related stress and psychological distress can be explained through variations in loneliness. To evaluate the directional nature of this hypothesized model, we evaluated reverse mediation models, in which we reversed the input variable and the mediator. Thus we tested whether associations between Loneliness and Psychological Distress can be explained by variations in Distancing-related stress. We included moderators for PPS and Living Alone and tested mediation both across and within participants (i.e., single level versus multilevel mediation). Although we observed significant mediation in both reverse models (see Table S19), Average distancing-related stress only explained 19.4% of the variance in associations between Average loneliness and Average Psychological Distress, in contrast to our hypothesized model in which loneliness explained 43.7% of the variance of associations between Average distancing-related stress and Average psychological distress. We evaluated model fit using leave-one-out cross-validation implemented using "loo" within the brms package[53] to compute the Bayesian LOO estimate of the expected log pointwise predictive density (ELPD). We performed model comparison between reversed models and hypothesized models (i.e., those reported in Table 8) using the function "loo_compare" in brms[53]. Model comparison revealed that our hypothesized single level model was a better fit for the data with ELPDs that exceeded 30 times the standard error of the difference between models (ELPD_diff = -1341.1, SE_diff = 44.6). Likewise, the reversed multilevel model indicated that changes in Distancing-related Stress explained only 6.3% of the variance between Loneliness and Psychological Distress, in contrast to the hypothesized model in which variations in Loneliness explained 25.5% of the variance between Distancing-related stress and Psychological Distress over time, and leave-one-out cross validation supported the hypothesized mode, indicating that ELPDs exceeded 80 times the standard error of the difference between models (ELPD_diff = -24235.1, SE_diff = 302.9). Finally, we note that we did not explore models that treated Psychological Distress as a predictor or mediator, to maintain consistency with our other analyses which treated this as an outcome.

Supplementary References

62. Gorman, B. mltools: Machine Learning Tools. R package version 0.3.5, https://CRAN.R-project.org/package=mltools. (2018).

63. Feinerer, I. & Hornik, K. tm: Text Mining Package. R package version 0.7-14. https://CRAN.R-project.org/package=tm. (2024).

64. Feinerer, I., Hornik, K. & Meyer, D. Text Mining Infrastructure in R. *Journal of Statistical Software* **25**, 1–54 (2008).

65. Lang, D. & Chien, G. wordcloud2: Create Word Cloud by ‘htmlwidget’. R package version 0.2.1. https://CRAN.R-project.org/package=wordcloud2. (2018)

66. R Core Team. R: A language and environment for statistical computing. R Foundation for Statistical Computing (1996).

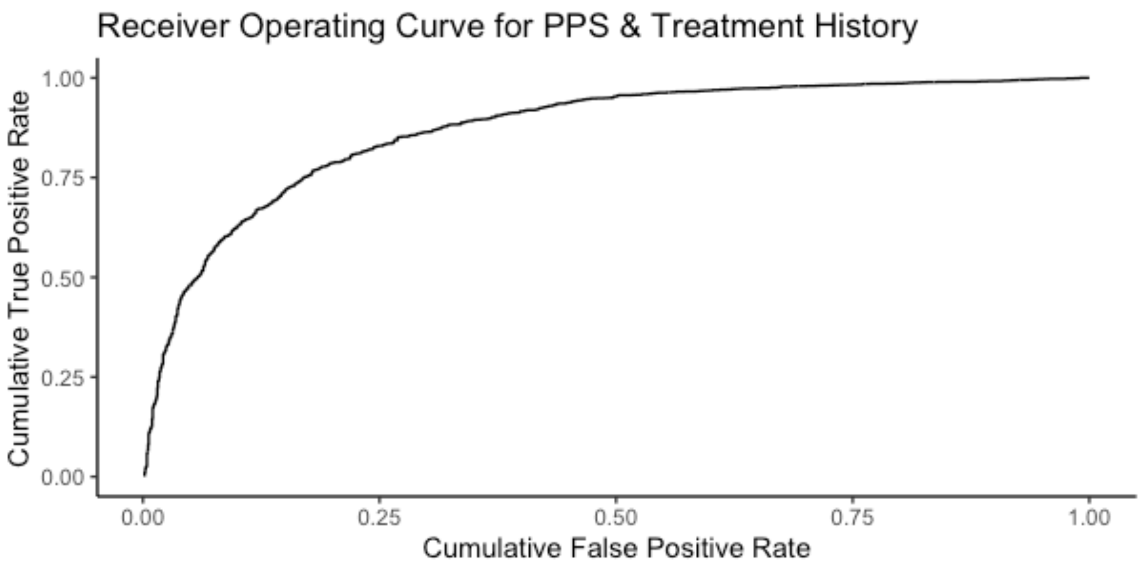

Figure S1. *Receiver Operating Characteristic for relationship between PPS and self-reported mental health treatment history.* This figure demonstrates the receiver operating characteristic (ROC) for the association between patient probability score (PPS) and self-reported treatment history in the present dataset (n = 3596). The area-under-the-curve of the ROC was 0.87.

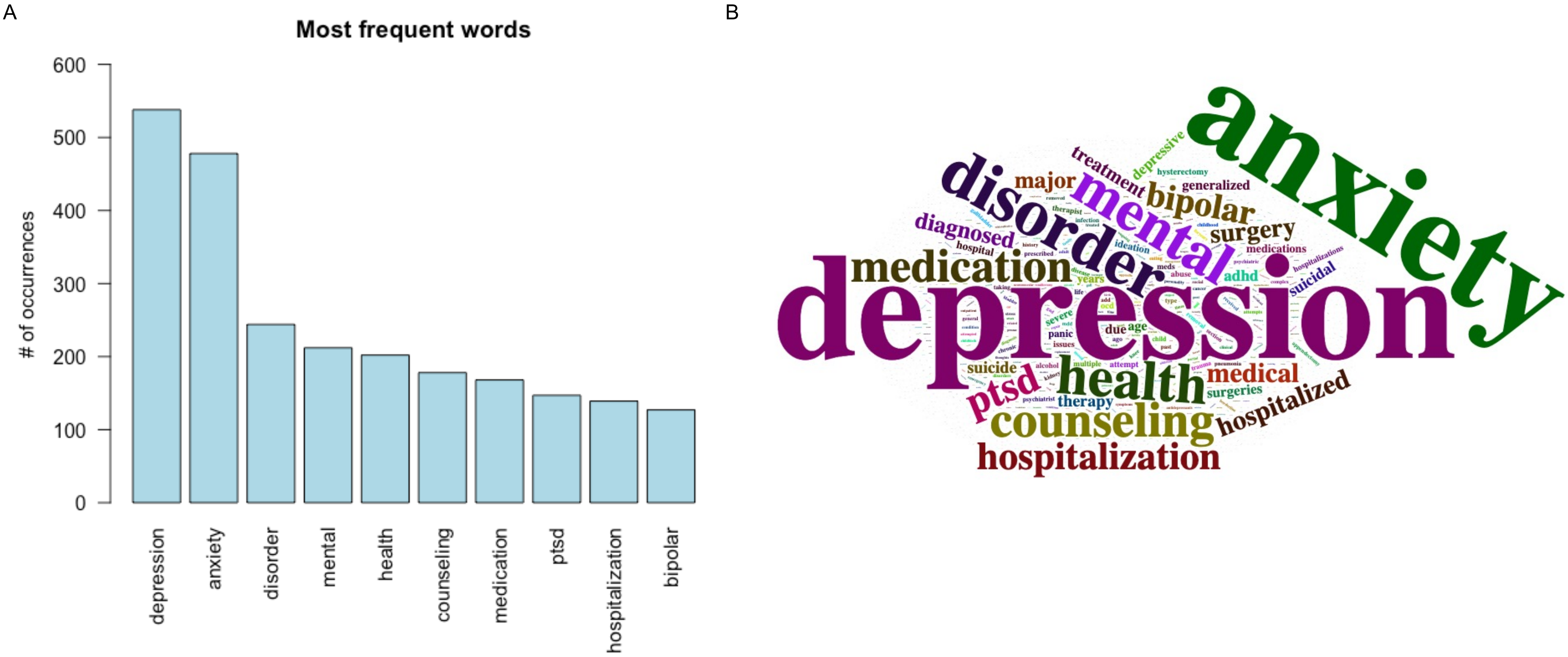

Figure S2. *Self-reported clinical history in participants with high PPS values.* We selected participants from the top quartile of PPS (scores ranging from 0.75 to 0.97; n = 899) and used natural language processing to identify the frequency of specific diagnoses. A) Frequency of term in self-reported clinical history in participants with high PPS values. B) Word cloud of terms used in self-reported clinical history in participants with high PPS values. Font size is proportional to frequency.

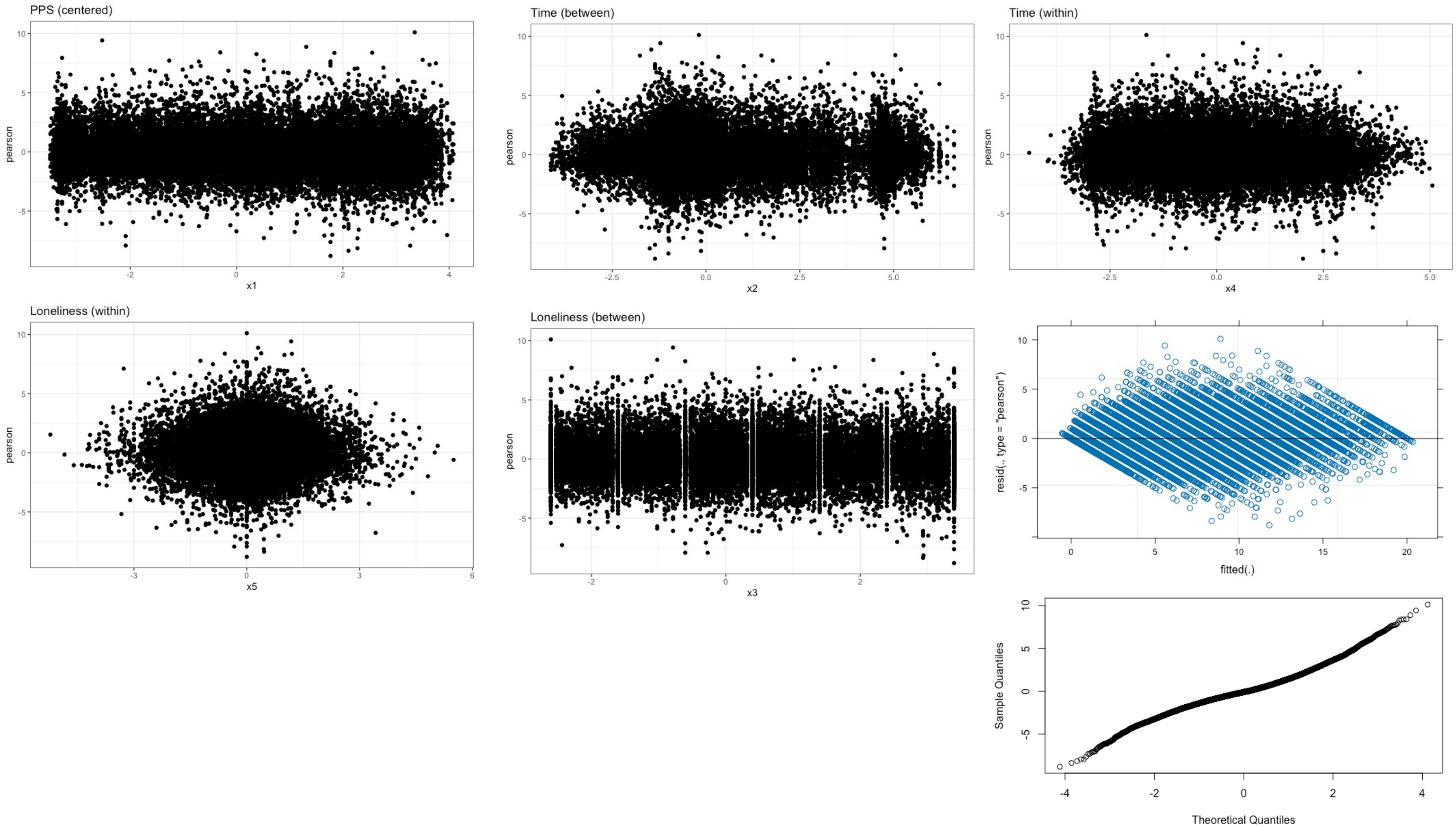

Figure S3. *Model validation.* This figure depicts residuals of the main model (Table 2) as a function of predictor, as well as the overall residuals relative to the fitted plot and the Q-Q plot.

Factor	Levels	Coefficient (CI, P-value)
PREDICTED_PATIENT_PROB	[0.2,1.0]	-0.00 (-0.06 to 0.05, p=0.919)
cov_gender	female	-
	male	0.02 (-0.01 to 0.05, p=0.235)
	nonconforming	-0.04 (-0.14 to 0.06, p=0.390)
	trans	0.15 (-0.06 to 0.36, p=0.167)
	other	0.11 (-0.07 to 0.29, p=0.239)
	missing	-0.00 (-0.10 to 0.10, p=0.974)
cov_race	caucasian	-
	AA	-0.02 (-0.07 to 0.04, p=0.623)
	AAPI	0.05 (-0.01 to 0.11, p=0.117)
	AI	-0.18 (-0.29 to -0.07, p=0.002)
	multiple	0.07 (-0.01 to 0.15, p=0.104)
	unknown	0.05 (-0.13 to 0.24, p=0.567)
	missing	-0.03 (-0.14 to 0.07, p=0.553)
cov_eth	notLatino	-
	Latino	-0.02 (-0.07 to 0.04, p=0.558)
	unknown	-0.26 (-0.37 to -0.14, p<0.001)
	missing	-0.00 (-0.06 to 0.06, p=0.912)
cov_setting	suburban	-
	urban	0.00 (-0.02 to 0.03, p=0.973)
	rural	-0.04 (-0.07 to -0.00, p=0.025)
	missing	0.00 (-0.17 to 0.17, p=0.981)
cov_educ	advancedprofessional	-
	ltadvanced	-0.03 (-0.05 to -0.00, p=0.022)
	ltBS	-0.09 (-0.12 to -0.06, p<0.001)
	ltAA	-0.08 (-0.15 to -0.01, p=0.023)
	missing	-0.02 (-0.24 to 0.19, p=0.853)
cov_age	[18.0,87.0]	0.00 (0.00 to 0.00, p<0.001)
Household_Size	[1.0,10.0]	-0.01 (-0.02 to -0.00, p=0.020)
living_alone	others	-
	alone	-0.00 (-0.04 to 0.03, p=0.789)
	missing	-

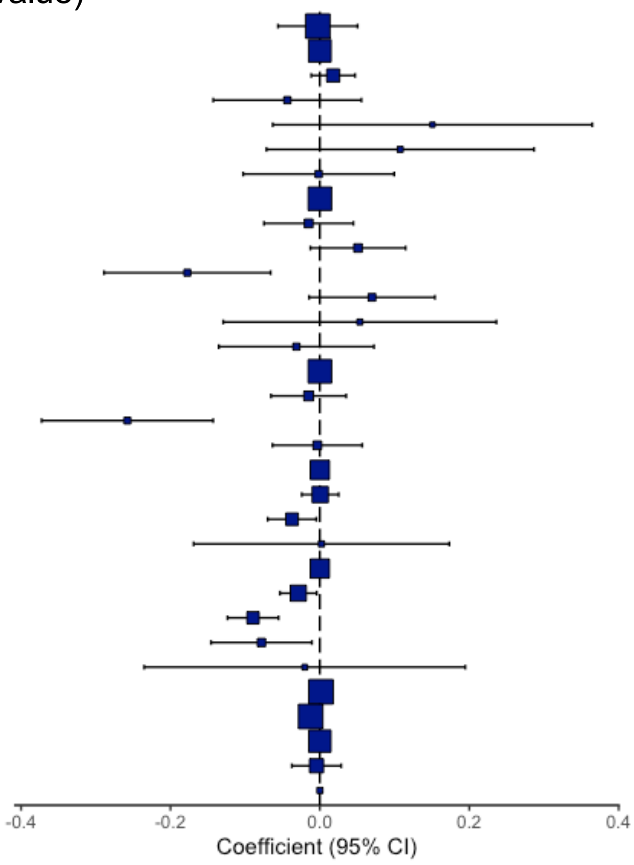

Figure S4. Likelihood of repeat response as a function of patient probability score, social isolation, and sociodemographic categories. This figure depicts results of a logistic model that tested whether PPS, demographics, or social isolation influenced participants' likelihood of replying across multiple intervals versus responding only once. Results and coefficients from the multivariate model are visualized using the function "coefficient_plot" from the R package finalfit. We note that p-values are two-sided and do not include multiplicity correction. In the coefficient plot, box sizes reflect relative sample size, error bars denote the 95% confidence intervals, and 0 represents the likelihood of repeat response for the reference group (white non-Hispanic suburban women with advanced degrees who live with others).

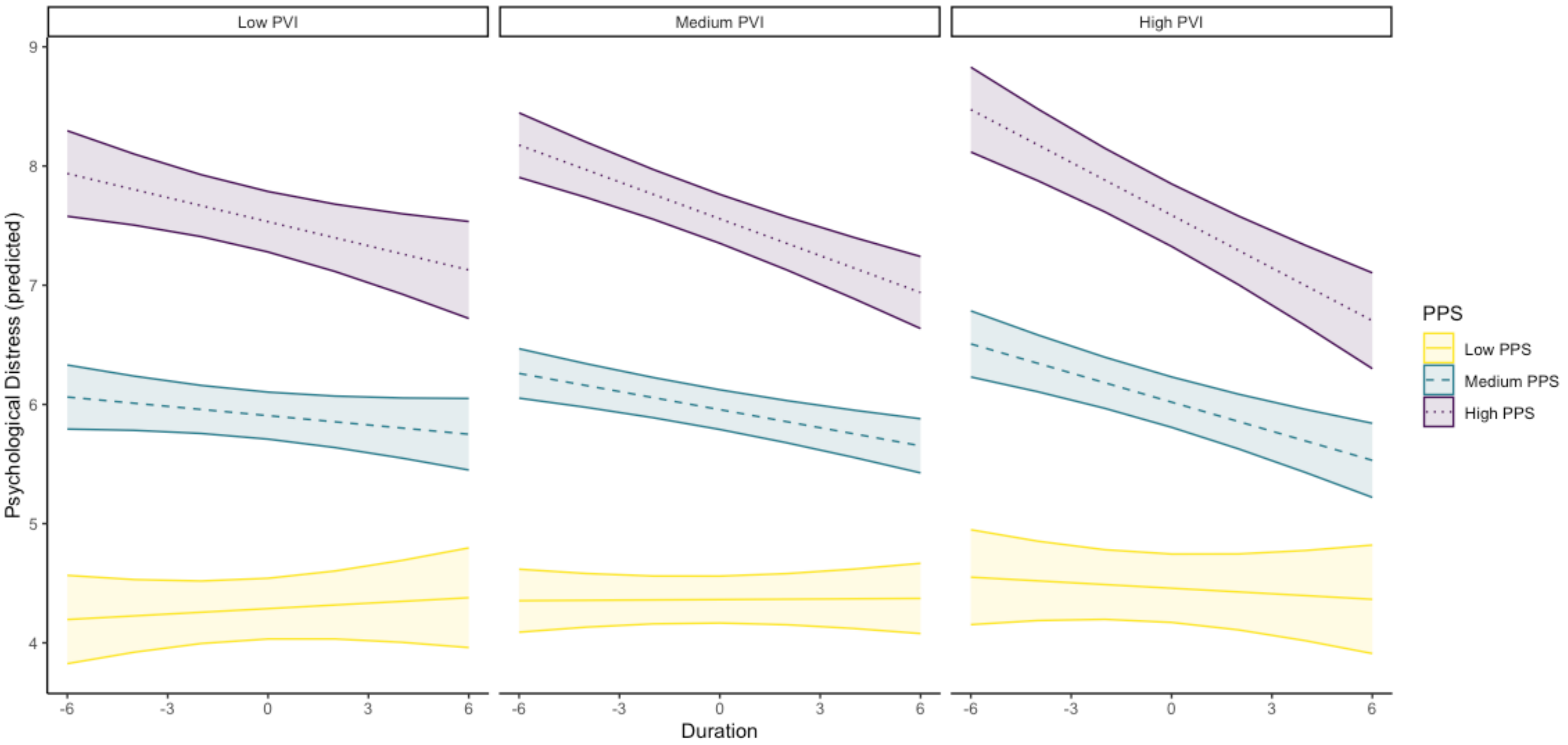

Figure S5. *Psychological distress as a function of pandemic vulnerability, PPS, and Duration.* We used linear mixed models to examine relationships when controlling for pandemic vulnerability index (PVI). Results were thresholded at p < .001 (two-sided, no multiplicity correction) in frequentist models and combined with Bayesian statistics to evaluate practical significance based on the region of partial equivalence (ROPE). We observed a practically significant three-way interaction between Duration, Average pandemic vulnerability index (PVI), and PPS (B = -1.47, CI = [-2.08, -0.53], p < .001; *b* = -0.11, 1.47% in ROPE), such that individuals with high PPS scores in areas with high pandemic vulnerability reported the largest reductions in psychological distress across time. The figure depicts model predictions generated using the function “ggpredict”. Error bands denote 95% confidence intervals. For complete results, see Table S4.

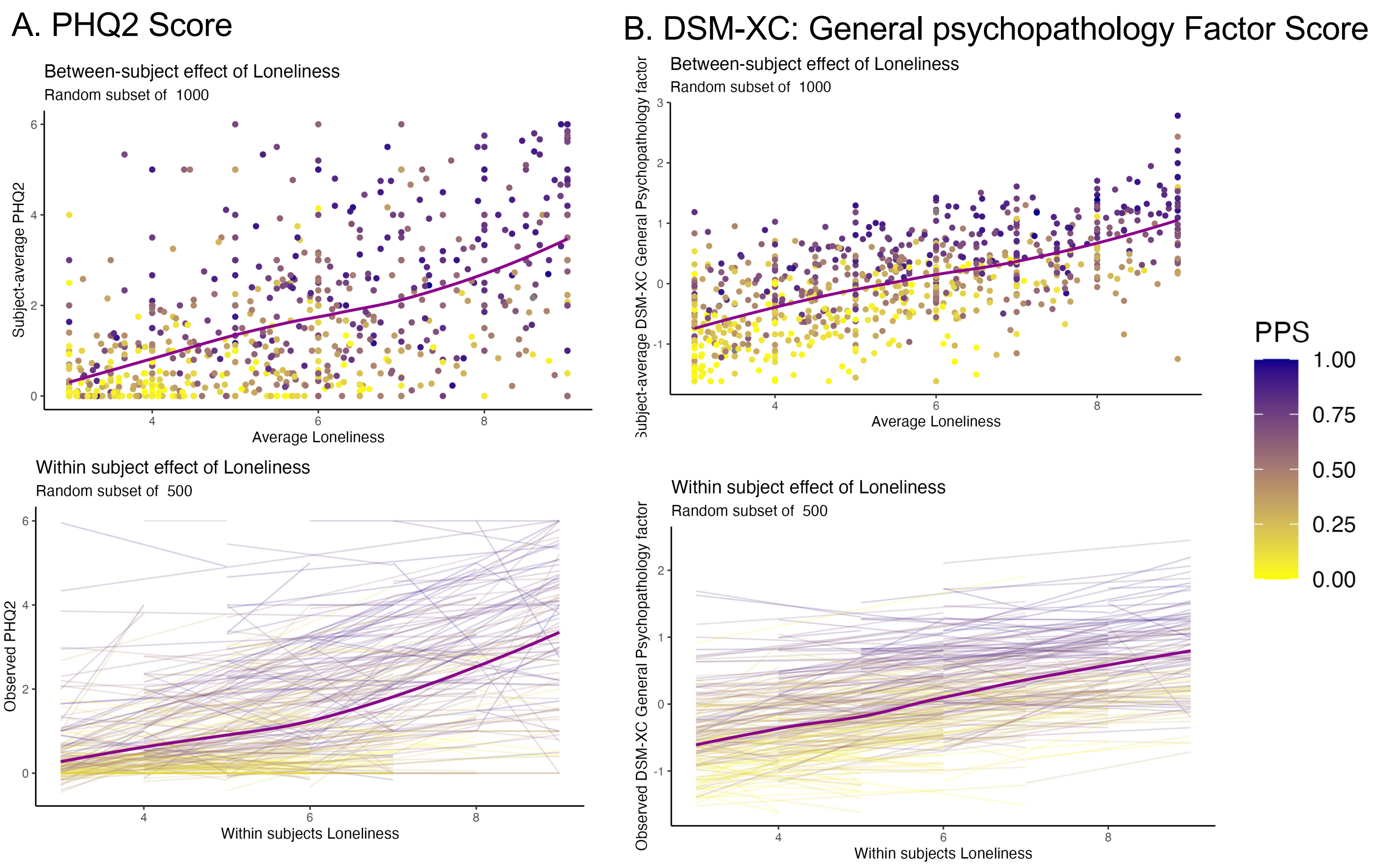

Figure S6. *Associations between loneliness, PPS, and other mental health outcome measures.* Conclusions were similar when we examined associations with other mental health outcome measures rather than Psychological Distress. We observed positive associations with loneliness both across subjects (top rows) and within subjects over time (bottom rows) for both PHQ2 scores (A) and the general psychopathology factor score from the DSM-XC (B).

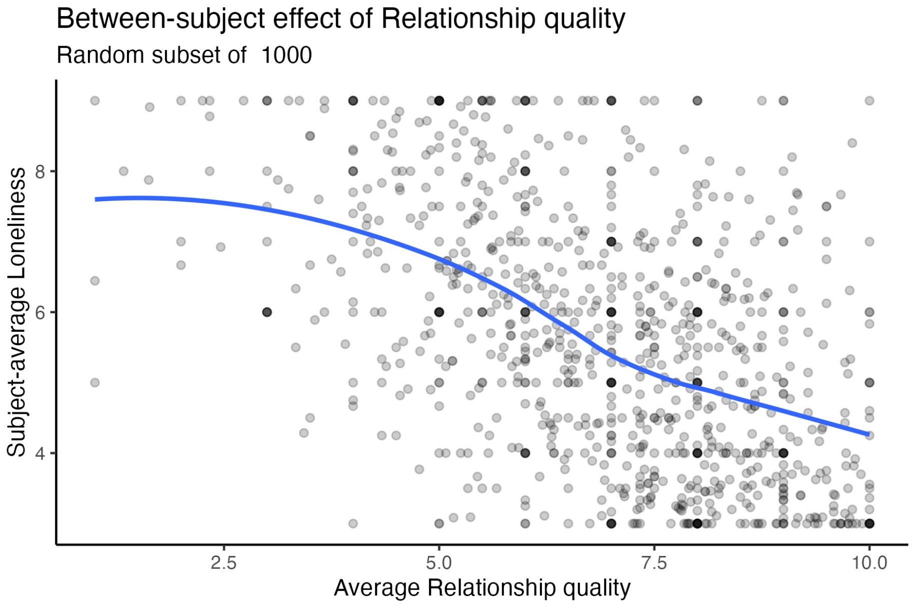

Figure S7. *Loneliness and relationship quality.* We observed practically significant associations between average relationship quality and average loneliness over the duration of study participation (see Table S11).

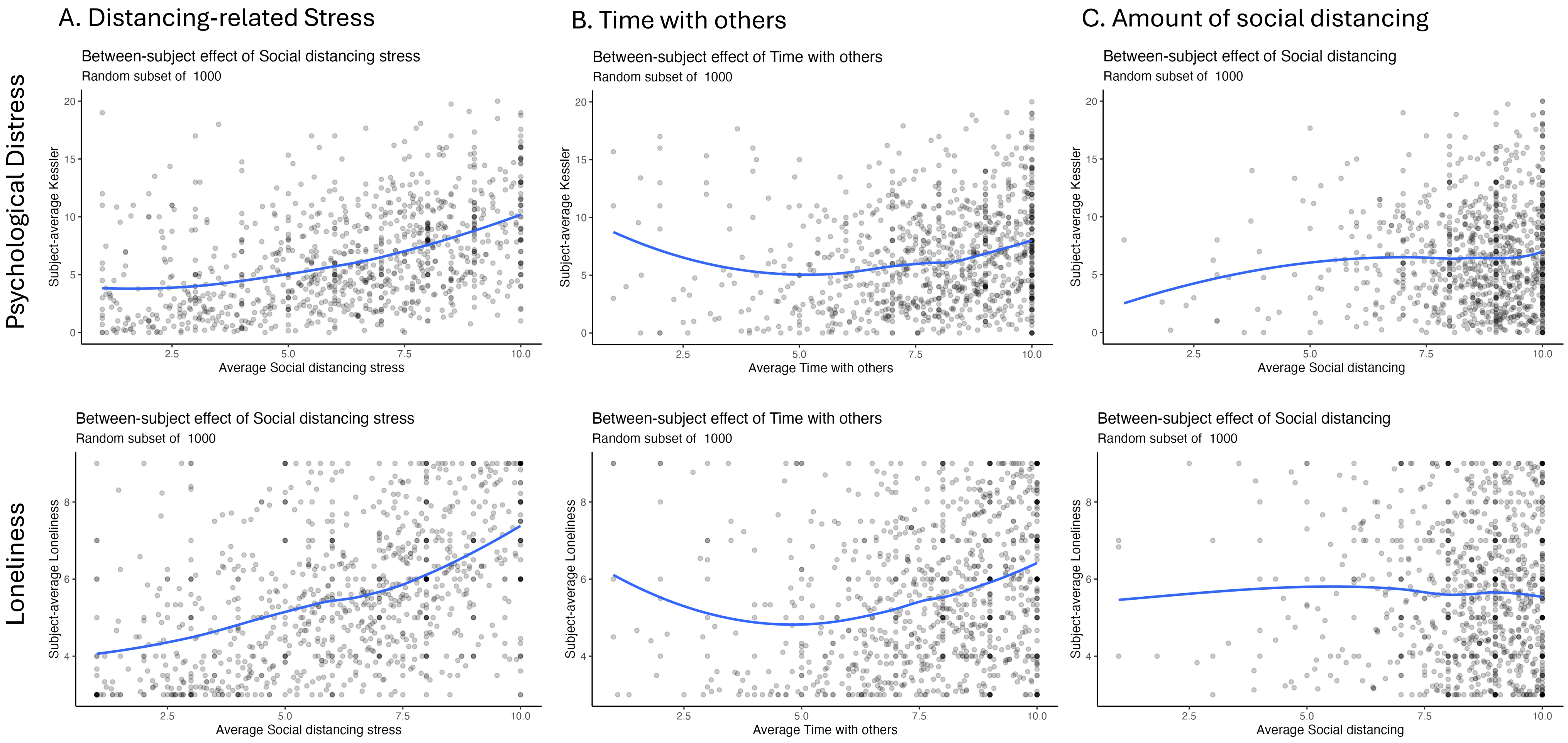

Figure S8. *Associations between self-reported social-distancing, psychological distress, and loneliness.* Although we observed positive associations with all measures when they were tested independently, only individual differences in distancing-related stress were found to be practically significant when all measures were included in the same model. For complete results, see Tables S14 and S15.

Table S1. Pair-wise correlations between mental health outcome measures.[a]

Visit week	Kessler-5, PHQ-2	PHQ-2, DSM-XC-G	Kessler-5, DSM-XC-G
0	0.79	0.83	0.88
2	0.78	0.82	0.88
4	0.79	0.81	0.88
6	0.80	0.81	0.88
8	0.79	0.81	0.88
10	0.80	0.81	0.89
12	0.80	0.81	0.88
14	0.81	0.81	0.88
16	0.80	0.81	0.88
18	0.80	0.80	0.88
20	0.81	0.81	0.88
22	0.81	0.80	0.89
24	0.81	0.80	0.88

[a.] This table presents pair-wise correlations between mental health outcomes (Kessler-5[38], PHQ-2[40], and a general mental health factor score from the DSM-XC[39,41]) as a function of visit week (Duration). Pearson correlations were computed using the “cor” function of the R program “stats”[66] and based on pairwise complete observations.

Table S2. Pair-wise correlations between social distancing measures.[b]

Visit week	Community Distancing, Distancing Magnitude	Distancing stress, Distancing Magnitude	Distancing Stress, Community Distancing	Time with Others, Distancing Magnitude	Time with Others, Distancing Stress	Time with Others, Community Distancing
0	0.10	-0.03	-0.10	0.19	0.38	0.01
2	0.05	-0.01	-0.11	0.18	0.37	0.00
4	0.01	-0.02	-0.05	0.21	0.39	0.01
6	-0.04	0.03	-0.03	0.25	0.42	0.01
8	-0.04	0.04	0.01	0.30	0.44	0.04
10	-0.04	0.02	0.00	0.27	0.41	-0.01
12	-0.04	0.00	0.03	0.24	0.41	0.01
14	-0.02	-0.02	0.01	0.24	0.41	0.03
16	0.05	0.02	-0.03	0.26	0.46	0.05
18	0.04	0.03	-0.01	0.24	0.48	0.02
20	0.01	0.03	-0.03	0.28	0.47	0.01
22	-0.07	0.06	0.03	0.27	0.47	0.03
24	-0.03	0.08	0.03	0.30	0.48	0.00

[b.] This table presents pair-wise correlations between social distancing measures, operationalized as responses to items embedded in the biweekly survey (Distancing magnitude = "How much have you been social distancing?"; Distancing stress = "How stressful has it been for you to maintain social distancing?"; Time with others = "How much has your time with other people changed compared to how you acted before the COVID-19 outbreak?") and community distancing data generated from the NIEHS Pandemic Vulnerability Index's "Social Distancing Metrics" (inverse scored to maintain consistency with other outcome measures, such that higher values reflect more distancing) [49]. Consistent with Table S1, Pearson correlations were computed using the "cor" function of the R program "stats"[66] and based on pairwise complete observations.

Table S3. Longitudinal model of distress with age and gender interactions.[c]

	LMER[d]					Standardized[e]		BRMS[f]		
term	estimate	std.error	t-statistic	df	p.value	Std. Coef.	95% CI	Median	95% CI	% in ROPE
**(Intercept)**	6.24	0.05	3488	119.38	0.000	0.00	[0.00, 0.00]	6.24	[6.14, 6.34]	0%
Age	-0.04	0.00	3497	-11.03	0.000	-0.15	[-0.17, -0.12]	-0.04	[-0.05, -0.03]	100%
Gender	0.15	0.07	3476	2.02	0.043	0.03	[0.00, 0.06]	0.15	[0.00, 0.30]	100%
**PPS**	0.80	0.02	3460	32.55	0.000	0.43	[0.41, 0.46]	0.80	[0.75, 0.85]	0%
Mean participation date (Time between)	-0.08	0.02	3577	-3.43	0.001	-0.05	[-0.07, -0.02]	-0.08	[-0.12, -0.03]	100%
Duration (Time over time)	-0.03	0.01	2634	-2.11	0.035	-0.02	[-0.04, 0.00]	-0.03	[-0.05, 0.00]	100%
**Mean loneliness (Loneliness between)**	0.97	0.03	3532	32.52	0.000	0.43	[0.41, 0.46]	0.97	[0.91, 1.03]	0%
**Loneliness over time (Loneliness over time)**	0.56	0.02	2042	29.81	0.000	0.26	[0.24, 0.27]	0.56	[0.52, 0.60]	0%
Age x PPS	0.00	0.00	3495	-2.54	0.011	-0.03	[-0.06, -0.01]	0.00	[-0.01, 0.00]	100%
Age x Mean participation date	0.00	0.00	3554	2.59	0.010	0.04	[0.01, 0.06]	0.00	[0.00, 0.01]	100%
PPS x Mean participation date	-0.03	0.01	3577	-3.04	0.002	-0.04	[-0.07, -0.01]	-0.03	[-0.05, -0.01]	100%
Age x Duration	0.00	0.00	2667	4.35	0.000	0.04	[0.02, 0.06]	0.00	[0.00, 0.01]	100%
Gender x Duration	-0.07	0.02	2669	-4.36	0.000	-0.05	[-0.07, -0.03]	-0.07	[-0.11, -0.04]	100%
PPS x Duration	-0.02	0.01	2592	-4.23	0.000	-0.04	[-0.06, -0.02]	-0.02	[-0.03, -0.01]	100%
Mean participation date x Duration	-0.03	0.01	2584	-5.75	0.000	-0.06	[-0.08, -0.04]	-0.03	[-0.04, -0.02]	100%
PPS x Mean loneliness	0.04	0.01	3491	3.10	0.002	0.04	[0.01, 0.06]	0.04	[0.02, 0.07]	100%
Duration x Mean loneliness	0.03	0.01	2555	3.61	0.000	0.04	[0.02, 0.06]	0.02	[0.01, 0.04]	100%
PPS x Loneliness over time	0.03	0.01	2067	2.89	0.004	0.02	[0.01, 0.04]	0.03	[0.01, 0.04]	100%
Mean loneliness x Loneliness over time	0.05	0.01	2638	4.20	0.000	0.03	[0.02, 0.05]	0.05	[0.03, 0.08]	100%
Age x PPS x Mean participation date	0.00	0.00	3576	2.19	0.029	0.03	[0.00, 0.06]	0.00	[0.00, 0.00]	100%
Age x PPS x Duration	0.00	0.00	2643	-2.20	0.028	-0.02	[-0.04, 0.00]	0.00	[0.00, 0.00]	100%
Age x PPS x Mean loneliness	0.00	0.00	3517	3.02	0.003	0.04	[0.01, 0.07]	0.00	[0.00, 0.00]	100%
Age x Mean participation date x Mean loneliness	0.00	0.00	3639	-2.18	0.030	-0.03	[-0.06, 0.00]	0.00	[0.00, 0.00]	100%
Mean participation date x Duration x Mean loneliness	0.01	0.00	2512	2.02	0.043	0.02	[0.00, 0.04]	0.01	[0.00, 0.01]	100%
Age x PPS x Mean participation date x Duration	0.00	0.00	2643	2.09	0.037	0.02	[0.00, 0.05]	0.00	[0.00, 0.00]	100%

Gender x PPS x Duration x Mean loneliness	0.01	0.00	2494	2.53	0.012	0.03	[0.01, 0.05]	0.01	[0.00, 0.02]	100%
PPS x Mean participation date x Duration x Mean loneliness	0.00	0.00	2475	2.95	0.003	0.03	[0.01, 0.05]	0.00	[0.00, 0.01]	100%
Age x Gender x Duration x Loneliness over time	0.00	0.00	22320	2.40	0.016	0.02	[0.00, 0.03]	0.00	[0.00, 0.00]	100%
Gender x Mean participation date x Mean loneliness x Loneliness over time	-0.03	0.01	3443	-3.28	0.001	-0.03	[-0.05, -0.01]	-0.03	[-0.05, -0.01]	100%
Age x Duration x Mean loneliness x Loneliness over time	0.00	0.00	20680	-2.40	0.017	-0.02	[-0.03, 0.00]	0.00	[0.00, 0.00]	100%
Age x Gender x PPS x Mean participation date x Loneliness over time	0.00	0.00	2760	-2.02	0.043	-0.02	[-0.04, 0.00]	0.00	[0.00, 0.00]	100%
Age x Gender x Mean participation date x Duration x Loneliness over time	0.00	0.00	21920	-2.31	0.021	-0.02	[-0.04, 0.00]	0.00	[0.00, 0.00]	100%
Age x Gender x Duration x Mean loneliness x Loneliness over time	0.00	0.00	20490	-2.70	0.007	-0.02	[-0.04, -0.01]	0.00	[0.00, 0.00]	100%
Gender x Mean participation date x Duration x Mean loneliness x Loneliness over time	-0.01	0.00	20820	-2.69	0.007	-0.02	[-0.04, -0.01]	-0.01	[-0.02, 0.00]	100%

[c]. This table reports results of linear mixed models predicting psychological distress (measured by the Kessler-5[38]) as a function of Time, Loneliness, Patient Probability Score (PPS), Age, and Gender in 3585 participants with sufficient data. The model is equivalent to Table 2 in the main manuscript, but tests for interactions with age and gender rather than modeling factors as demographic covariates. Thus the intercept reflects the mean score, rather than the mean for the dominant subgroup. All predictors were mean-centered to facilitate interpretation of coefficients and interactions. The table reports terms whose associations were statistically significant based on frequentist models (p < .05, two-sided, no multiplicity correction). Effects that are both statistically and practically significant (< 2.5% of posterior estimates in ROPE) are bolded, effects that are statistically significant but of uncertain practical significance (between 2.5% and 95% of posterior estimates in ROPE) are italicized, and effects that are consistent with the null hypothesis (>97.5% in ROPE) are written in plain text. For complete results, including statistics for non-significant factors, see the spreadsheet “Complete_Supplementary_Tables.xls” available at https://osf.io/e7jrd/.

[d.] Frequentist results were evaluated using the function lmer from the R package lme4[51] using the following model:

distress~ Age*Gender*PPS*Time between*Time within*Loneliness between*Loneliness within + (1 + Time within + Loneliness within | SUBJECT_NUMBER)

[e.] Bayesian results were evaluated using the function brms from the R package brms [53] using the same model specified for lme4. See Table 2 for details of model specification.

[f.] We evaluated pseudo-standardized coefficients and confidence Durations using the package “effectsize” [52].

Table S4. Main longitudinal model controlling for changes in pandemic vulnerability.[g]

		LMER					Standardized		BRMS		
		Estimate	Std. Error	df	t value	p-value	Std. Coef.	95% CI	Median	95% CI	% in ROPE
Main effects	**(Intercept)**	1.04	0.18	3392	5.71	0.000	0.00	[0.00, 0.00]	1.05	[0.70, 1.40]	0.04%
	**Mean loneliness**	0.93	0.03	3417	31.25	0.000	0.42	[0.39, 0.45]	0.93	[0.88, 0.99]	0%
	PPS	0.59	0.08	3345	7.68	0.000	0.32	[0.24, 0.40]	0.59	[0.44, 0.74]	3.08%
	Loneliness over time	0.25	0.08	2810	3.19	0.001	0.11	[0.04, 0.19]	0.25	[0.10, 0.40]	99.41%
	Time within	-0.14	0.04	2494	-3.43	0.001	-0.12	[-0.19, -0.05]	-0.15	[-0.23, -0.07]	100%
	Mean participation date	-0.18	0.08	3454	-2.29	0.022	-0.11	[-0.20, -0.02]	-0.17	[-0.33, -0.03]	100%
	PVI over time (PVI over time) – *N.S.*	-3.28	2.95	2800	-1.12	0.265	-0.03	[-0.09, 0.02]	-1.31	[-4.95, 2.32]	15.24%
	Mean PVI (PVI between) – *N.S.*	1.57	3.79	3358	0.41	0.679	0.02	[-0.07, 0.10]	0.52	[-3.52, 4.59]	16.59%
Interactions	**Duration x Mean PVI x PPS**	-1.47	0.42	2622	-3.50	0.000	-0.11	[-0.17, -0.05]	-1.31	[-2.08, -0.53]	1.47%
	Mean PVI x Loneliness over time	3.65	1.77	3002	2.07	0.039	0.07	[0.00, 0.14]	1.95	[-0.78, 4.64]	9.70%
	PVI over time x Loneliness over time	-7.87	3.52	18770	-2.24	0.025	-0.08	[-0.15, -0.01]	-2.24	[-6.13, 1.56]	9.99%
	Mean participation date x Duration x PVI over time x Mean PVI x Loneliness over time	57.42	25.85	11860	2.22	0.026	0.09	[0.01, 0.16]	0.39	[-4.50, 5.19]	14.16%
	Mean participation date x PVI over time x Mean PVI	-92.99	36.90	2051	-2.52	0.012	-0.06	[-0.11, -0.01]	-0.35	[-5.17, 4.39]	14.49%
	Mean participation date x Duration x PVI over time x Mean PVI x Mean loneliness x Loneliness over time	-11.22	4.54	11440	-2.47	0.013	-0.10	[-0.17, -0.02]	-1.14	[-3.19, 0.86]	18.66%
	Mean participation date x PVI over time x Mean PVI x Mean loneliness	16.02	6.25	1888	2.56	0.010	0.06	[0.02, 0.11]	0.56	[-2.35, 3.46]	21.88%
	PVI over time x Mean loneliness x Loneliness over time	1.20	0.61	18970	1.98	0.047	0.07	[0.00, 0.15]	0.28	[-0.41, 1.00]	65.32%
	Mean PVI x Mean loneliness x Loneliness over time	-0.64	0.30	2978	-2.09	0.037	-0.08	[-0.15, 0.00]	-0.35	[-0.83, 0.13]	65.79%
	Duration x Mean PVI x PPS x Mean loneliness	0.25	0.07	2674	3.34	0.001	0.11	[0.04, 0.17]	0.23	[0.09, 0.36]	99.91%

	Mean loneliness x Loneliness over time	0.06	0.01	2722	4.07	0.000	0.15	[0.08, 0.22]	0.06	[0.03, 0.08]	100%
	Mean participation date x Duration	-0.07	0.02	2280	-3.79	0.000	-0.14	[-0.21, -0.07]	-0.07	[-0.11, -0.03]	100%
	Mean participation date x Duration x PPS	-0.03	0.01	2252	-3.67	0.000	-0.12	[-0.19, -0.06]	-0.03	[-0.04, -0.01]	100%
	PPS x Mean loneliness	0.03	0.01	3364	2.40	0.016	0.10	[0.02, 0.18]	0.03	[0.00, 0.06]	100%
	Mean participation date x Duration x PPS x Mean loneliness	0.00	0.00	2265	3.24	0.001	0.10	[0.04, 0.16]	0.00	[0.00, 0.01]	100%
	Duration x Mean loneliness	0.02	0.01	2515	2.71	0.007	0.09	[0.03, 0.16]	0.02	[0.01, 0.04]	100%
	Mean participation date x Duration x Mean loneliness	0.01	0.00	2292	2.33	0.020	0.08	[0.01, 0.14]	0.01	[0.00, 0.01]	100%
	PPS x Loneliness over time	0.08	0.04	2936	2.11	0.035	0.07	[0.01, 0.14]	0.08	[0.01, 0.15]	100%
	Mean participation date x PPS x Loneliness over time	-0.03	0.02	3123	-2.00	0.045	-0.07	[-0.14, 0.00]	-0.03	[-0.07, 0.00]	100%
	Mean participation date x PPS x Mean loneliness x Loneliness over time	0.01	0.00	2949	2.11	0.035	0.07	[0.01, 0.14]	0.01	[0.00, 0.01]	100%
Covariates	*Education: less than Associates degree*	1.00	0.31	3513	3.25	0.001	0.04	[0.02, 0.06]	0.99	[0.41, 1.59]	3.48%
	Racial identity: AA	-0.73	0.26	3503	-2.77	0.006	-0.03	[-0.06, -0.01]	-0.72	[-1.24, -0.20]	15.18%
	Education: less than bachelors degree	0.59	0.15	3434	3.89	0.000	0.05	[0.02, 0.08]	0.58	[0.29, 0.87]	18.58%
	Racial identity: AAPI	-0.54	0.27	3323	-2.00	0.045	-0.02	[-0.05, 0.00]	-0.54	[-1.06, -0.01]	37.21%
	Gender: Man	-0.47	0.13	3310	-3.68	0.000	-0.04	[-0.07, -0.02]	-0.47	[-0.73, -0.21]	43.88%
	Age	-0.04	0.00	3274	-11.11	0.000	-0.14	[-0.16, -0.11]	-0.04	[-0.04, -0.03]	100%

[g.] The table reports terms whose associations with psychological distress (Kessler-5[38] scores) were statistically significant based on frequentist models (p < .05, two-sided, no multiplicity correction), as well as results for associations with Pandemic Vulnerability Index (PVI; main effects written in red), which was not included in the main longitudinal model reported in Table 2 in the main manuscript. 3411 participants had sufficient data and were included in analyses. Practically significant factors are bolded, statistically significant factors of undecided practical significance are italicized, and effects that were consistent with the null hypothesis are reported in plain text. Additional factors that were included in the model but were not statistically significant (p > 0.05) are omitted for the purpose of brevity and are identical to non-significant covariates and interactions reported in Table 2 in the main manuscript. For complete results, including statistics for non-significant factors, see the spreadsheet “Complete_Supplementary_Tables.xls” available at https://osf.io/e7jrd/. The complete model was:

distress ~ Gender + Education + Ethnicity + Racial identity + Setting + Age + Time between * Time within * PVI between * PVI within * PPS * Loneliness between * Loneliness within + (1 + Time within + PVI within + Loneliness within | SUBJECT_NUMBER)

Table S5. Longitudinal model of mental health as operationalized by PHQ2.[h]

		LMER					Standardized		BRMS		
	term	estimate	std.error	t-statistic	df	p-value	Coefficient (std.)	95% CI	Median	95% CI	% in ROPE
Main effects	**(Intercept)**	1.31	0.03	3361	41.49	0.000	0.00	[0.00, 0.00]	1.31	[1.25, 1.37]	0%
	**Mean loneliness**	0.34	0.01	3499	31.06	0.000	0.43	[0.40, 0.46]	0.34	[0.32, 0.36]	0%
	**Patient probability score (PPS)**	0.27	0.01	3375	28.51	0.000	0.41	[0.38, 0.44]	0.26	[0.25, 0.28]	0%
	**Loneliness over time**	0.25	0.01	1889	29.23	0.000	0.23	[0.22, 0.25]	0.25	[0.23, 0.26]	0%
	Duration	-0.03	0.00	2562	-7.14	0.000	-0.06	[-0.08, -0.04]	-0.03	[-0.04, -0.03]	100%
	Mean participation date	-0.02	0.01	3562	-2.79	0.005	-0.04	[-0.06, -0.01]	-0.02	[-0.04, -0.01]	100%
Interactions	PPS x Mean loneliness	0.05	0.00	3404	10.40	0.000	0.13	[0.10, 0.15]	0.05	[0.04, 0.06]	100%
	PPS x Duration	-0.01	0.00	2443	-5.33	0.000	-0.05	[-0.06, -0.03]	-0.01	[-0.02, -0.01]	100%
	PPS x Loneliness over time	0.02	0.00	1852	6.26	0.000	0.05	[0.03, 0.06]	0.02	[0.02, 0.03]	100%
	Mean loneliness x Loneliness over time	0.04	0.01	2490	6.27	0.000	0.05	[0.03, 0.06]	0.04	[0.03, 0.05]	100%
	PPS x Mean participation date	-0.01	0.00	3567	-2.94	0.003	-0.04	[-0.07, -0.01]	-0.01	[-0.02, 0.00]	100%
	Mean participation date x Duration x Mean loneliness	0.00	0.00	2405	2.07	0.039	0.02	[0.00, 0.03]	0.00	[0.00, 0.01]	100%
	Mean participation date x Mean loneliness x Loneliness over time	-0.01	0.00	2726	-2.00	0.045	-0.01	[-0.03, 0.00]	-0.01	[-0.01, 0.00]	100%
Covariates	**Education: less than bachelors**	0.28	0.06	3564	5.01	0.000	0.07	[0.04, 0.09]	0.28	[0.17, 0.39]	2.43%
	Education: less than associates	0.39	0.11	3683	3.49	0.000	0.04	[0.02, 0.07]	0.38	[0.16, 0.60]	2.96%
	Racial identity: AAPI	-0.26	0.10	3399	-2.60	0.009	-0.03	[-0.06, -0.01]	-0.26	[-0.46, -0.06]	19.23%
	Education: less than advanced degree	0.08	0.04	3338	2.09	0.036	0.03	[0.00, 0.05]	0.08	[0.00, 0.16]	98.85%

h. The table reports terms whose associations with PHQ-2 scores were statistically significant based on frequentist models ($p < .05$, two-sided, no multiplicity correction) in 3587 participants with sufficient data. Factors that had significant associations with PHQ-2, but not psychological distress (Kessler-5[38] scores), are denoted in blue. Factors that predicted psychological distress but not PHQ-2 were Man gender, Age, Duration x Mean loneliness, and Mean participation date x Duration. Practically significant factors are bolded, statistically significant factors of undecided practical significance are italicized, and effects that were consistent with the null

hypothesis are reported in plain text. Additional factors that were included in the model but were not statistically significant are omitted for the purpose of brevity. For complete results, including statistics for non-significant factors, see the spreadsheet “Complete_Supplementary_Tables.xls” available at https://osf.io/e7jrd/. The complete model was:

PHQ-2 ~ Gender + Education + Ethnicity + Racial identity + Setting + Age + PPS*Time between + PPS*Time within + PPS*Loneliness between*Loneliness within +Loneliness between*Loneliness within*Time between*Time within + (1 + Time within + Loneliness within | SUBJECT_NUMBER)

Table S6. Longitudinal model of mental health as operationalized by DSM-XC general mental health factor score.[i]

		LMER					Standardized		BRMS		
	Term	estimate	Std error	df	t-statistic	p.value	Coefficient (std.)	95% CI	Median	95% CI	% in ROPE
	**Patient prediction score (PPS)**	0.16	0.00	3502	38.28	0.000	0.47	[0.44, 0.49]	0.16	[0.15, 0.17]	0%
	**Mean loneliness**	0.18	0.00	3568	36.95	0.000	0.44	[0.41, 0.46]	0.18	[0.17, 0.19]	0%
	**Loneliness over time**	0.10	0.00	1837	35.50	0.000	0.28	[0.26, 0.29]	0.10	[0.09, 0.11]	0%
	Duration	-0.02	0.00	2637	-8.99	0.000	-0.08	[-0.10, -0.06]	-0.02	[-0.02, -0.01]	100%
Main effects	Mean participation date	-0.01	0.00	3631	-2.56	0.010	-0.03	[-0.05, -0.01]	-0.01	[-0.02, 0.00]	100%
	PPS x Mean participation date	-0.01	0.00	3635	-4.34	0.000	-0.05	[-0.07, -0.03]	-0.01	[-0.01, 0.00]	100%
	Mean loneliness x Duration	0.01	0.00	2571	5.17	0.000	0.05	[0.03, 0.07]	0.01	[0.00, 0.01]	100%
	Mean loneliness x Mean participation date	0.01	0.00	3725	3.68	0.000	0.04	[0.02, 0.07]	0.01	[0.00, 0.01]	100%
	Mean participation date x Duration	0.00	0.00	2613	-4.42	0.000	-0.04	[-0.06, -0.02]	0.00	[-0.01, 0.00]	100%
	Mean participation date x Duration x Mean loneliness	0.00	0.00	2517	2.90	0.004	0.03	[0.01, 0.05]	0.00	[0.00, 0.00]	100%
Interactions	PPS x Duration	0.00	0.00	2506	-2.43	0.015	-0.02	[-0.04, 0.00]	0.00	[0.00, 0.00]	100%
	**Education: less than associates**	0.20	0.05	3738	4.26	0.000	0.05	[0.02, 0.07]	0.20	[0.11, 0.30]	0.54%
	**Education: less than bachelors**	0.13	0.02	3624	5.34	0.000	0.06	[0.04, 0.08]	0.13	[0.08, 0.17]	2.45%
	Ethnicity: unknown	0.25	0.09	3943	2.86	0.004	0.03	[0.01, 0.05]	0.25	[0.07, 0.41]	3.00%
	Gender: Man	-0.05	0.02	3452	-2.45	0.014	-0.03	[-0.05, -0.01]	-0.05	[-0.09, -0.01]	92.93%
	Education: less than advanced degree	0.04	0.02	3429	2.24	0.025	0.02	[0.00, 0.05]	0.04	[0.00, 0.07]	99.58%
Covariates	Age	-0.01	0.00	3423	-9.67	0.000	-0.10	[-0.12, -0.08]	-0.01	[-0.01, 0.00]	100%

[i.] The table reports terms whose associations with a general mental health factor from the DSM-XC[41] were statistically significant based on frequentist models (p < .05, two-sided, no multiplicity correction) in 3563 participants with sufficient data. Factors that had significant associations with the general mental health factor, but not psychological distress (Kessler-5[38] scores), are denoted in blue font. The only factors that predicted psychological distress but not general mental health were interactions between mean loneliness and loneliness over time. Practically significant factors are bolded, statistically significant factors of undecided practical significance are italicized, and effects that were consistent with the null hypothesis are reported in plain text. Additional factors that were included

in the model but were not statistically significant are omitted for the purpose of brevity. For complete results, including statistics for non-significant factors, see the spreadsheet “Complete_Supplementary_Tables.xls” available at https://osf.io/e7jrd/. The complete model was:

> DSM-XC Bifac-G ~ Gender + Education + Ethnicity + Racial identity + Setting + Age + PPS*Time between + PPS*Time within + PPS*Loneliness between*Loneliness within +Loneliness between*Loneliness within*Time between*Time within + (1 + Time within + Loneliness within | SUBJECT_NUMBER)

Table S7. Longitudinal model of clinically-significant mental health.[j]

	term	estimate	std.error	z-score	p.value	Coefficient (std.)	95% CI
Main effects	(Intercept)	-2.51	0.10	-24.94	0.000	0.00	[0.00, 0.00]
	Mean loneliness	0.88	0.04	24.85	0.000	1.58	[1.45, 1.70]
	Patient prediction score (PPS)	0.72	0.03	23.85	0.000	1.57	[1.44, 1.70]
	Loneliness over time	0.65	0.04	17.18	0.000	0.63	[0.56, 0.71]
	Duration	-0.14	0.03	-5.36	0.000	-0.24	[-0.33, -0.15]
	Mean participation date	-0.08	0.02	-3.35	0.001	-0.19	[-0.30, -0.08]
Interactions	PPS x Mean participation date	-0.03	0.01	-2.67	0.008	-0.16	[-0.28, -0.04]
	Mean loneliness x Duration	0.03	0.01	2.48	0.013	0.10	[0.02, 0.17]
	Mean participation date x Duration	-0.02	0.01	-2.71	0.007	-0.10	[-0.17, -0.03]
	Mean participation date x Mean loneliness x Duration	0.01	0.01	2.79	0.005	0.10	[0.03, 0.18]
	Duration x Loneliness over time	0.05	0.02	3.24	0.001	0.09	[0.04, 0.15]
	Mean participation date x Mean loneliness x Loneliness over time	-0.03	0.01	-2.90	0.004	-0.08	[-0.14, -0.03]
Covariates	Cov: Age	-0.01	0.00	-4.07	0.000	-0.22	[-0.33, -0.11]
	Education: less than bachelors	0.63	0.16	3.93	0.000	0.21	[0.11, 0.32]
	Gender: Man	-0.32	0.15	-2.17	0.030	-0.12	[-0.23, -0.01]

[j.] The table reports terms that were associated with one's likelihood of reporting clinically significant values on any mental health outcome based on logistic regression in 3583 participants with sufficient data. We report factors that were statistically significant based on frequentist statistics ($p < .05$, two-sided, no multiplicity correction). The complete model was:

ClinicalScore ~ Gender + Education + Ethnicity + Racial identity + Setting + Age + PPS*Time between + PPS*Time within + PPS*Loneliness between*Loneliness within +Loneliness between*Loneliness within*Time between*Time within + (1 + Time within + Loneliness within | SUBJECT_NUMBER)

Table S8. Longitudinal model of distress as a function of household size.[j]

		LMER					Standardized		BRMS		
	term	estimate	Std error	t-statistic	df	p-value	Coefficient (std.)	95% CI	Median	95% CI	% in ROPE
	**(Intercept)**	6.25	0.08	3455	73.88	0.000	0.00	[0.00, 0.00]	6.25	[6.09, 6.41]	0%
	**Mean loneliness**	0.98	0.03	3529	32.94	0.000	0.44	[0.41, 0.46]	0.98	[0.91, 1.04]	0%
	**PPS**	0.77	0.03	3451	30.54	0.000	0.41	[0.39, 0.44]	0.77	[0.72, 0.82]	0%
Main effects	**Loneliness over time**	0.57	0.01	22330	39.40	0.000	0.26	[0.25, 0.27]	0.57	[0.54, 0.60]	0%
	Mean household size	0.29	0.05	3535	5.74	0.000	0.08	[0.05, 0.10]	0.3	[0.19, 0.40]	99.80%
	Mean participation date	-0.07	0.02	3592	-3.11	0.002	-0.04	[-0.07, -0.02]	-0.07	[-0.12, -0.03]	100%
	Duration	-0.03	0.01	2671	-2.03	0.042	-0.02	[-0.04, 0.00]	-0.03	[-0.05, 0.00]	100%
	Household size over time	0.10	0.05	21630	2.12	0.034	0.02	[0.00, 0.04]	0.10	[0.01, 0.19]	100%
	Mean participation date x Duration	-0.03	0.01	2698	-5.13	0.000	-0.06	[-0.08, -0.03]	-0.03	[-0.04, -0.02]	100%
	Mean participation date x PPS	-0.04	0.01	3609	-3.54	0.000	-0.05	[-0.07, -0.02]	-0.04	[-0.06, -0.02]	100%
	Duration x PPS	-0.03	0.01	2659	-5.04	0.000	-0.05	[-0.07, -0.03]	-0.03	[-0.04, -0.02]	100%
	Mean loneliness x Duration	0.03	0.01	2647	4.08	0.000	0.04	[0.02, 0.06]	0.03	[0.02, 0.04]	100%
	Mean loneliness x Loneliness over time x Household size over time x Mean household size	-0.06	0.02	22720	-2.93	0.003	-0.04	[-0.07, -0.01]	-0.06	[-0.10, -0.02]	100%
	Mean participation date x Duration x Household size over time x Mean household size x PPS	0.02	0.01	22130	2.92	0.004	0.04	[0.01, 0.06]	0.02	[0.01, 0.04]	100%
	Mean loneliness x Loneliness over time	0.05	0.01	22340	5.17	0.000	0.03	[0.02, 0.05]	0.05	[0.03, 0.07]	100%
	Mean loneliness x Mean household size	0.06	0.03	3545	2.22	0.026	0.03	[0.00, 0.06]	0.06	[0.01, 0.11]	100%
	Mean loneliness x PPS	0.04	0.01	3489	2.81	0.005	0.03	[0.01, 0.06]	0.04	[0.01, 0.06]	100%
	Mean loneliness x Mean participation date x Duration	0.01	0.00	2684	2.53	0.011	0.03	[0.01, 0.05]	0.01	[0.00, 0.02]	100%
Interactions	Mean loneliness x Mean participation date x Duration x PPS	0.00	0.00	2668	2.74	0.006	0.03	[0.01, 0.05]	0.00	[0.00, 0.01]	100%
	Mean participation date x Duration x Household size over time x PPS	-0.02	0.01	23320	-3.11	0.002	-0.03	[-0.05, -0.01]	-0.02	[-0.04, -0.01]	100%
	Mean loneliness x Mean participation date x Duration x Household size over time x Mean household size	-0.02	0.01	23460	-2.44	0.015	-0.03	[-0.06, -0.01]	-0.02	[-0.04, 0.00]	100%
	Loneliness over time x PPS	0.02	0.01	22310	3.56	0.000	0.02	[0.01, 0.04]	0.02	[0.01, 0.04]	100%
	Mean loneliness x Household size over time x PPS	-0.03	0.01	21650	-2.07	0.038	-0.02	[-0.04, 0.00]	-0.03	[-0.05, 0.00]	100%
	Mean loneliness x Mean participation date x Duration x Household size over time	0.02	0.01	23230	2.46	0.014	0.02	[0.00, 0.04]	0.02	[0.00, 0.04]	100%
	Mean loneliness x loneliness over time x Mean participation date x Household size over time x PPS	-0.01	0.01	22990	-1.99	0.046	-0.02	[-0.04, 0.00]	-0.01	[-0.03, 0.00]	100%

	Mean loneliness x Loneliness over time x PPS	-0.01	0.00	22340	-2.07	0.039	-0.01	[-0.03, 0.00]	-0.01	[-0.02, 0.00]	100%
Covariates	*Ethnicity: Unknown*	1.01	0.51	4043	1.98	0.048	0.02	[0.00, 0.05]	0.97	[0.00, 1.93]	14.52%
	Education: Less than Associates	0.75	0.29	3691	2.58	0.010	0.03	[0.01, 0.05]	0.74	[0.20, 1.32]	15.07%
	Education: Less than Bachelors	0.53	0.15	3598	3.57	0.000	0.04	[0.02, 0.07]	0.53	[0.24, 0.81]	29.79%
	Racial identity: African-American	-0.58	0.26	3669	-2.26	0.024	-0.03	[-0.05, 0.00]	-0.56	[-1.07, -0.07]	32.35%
	Gender: Man	-0.51	0.12	3435	-4.10	0.000	-0.05	[-0.07, -0.02]	-0.51	[-0.75, -0.27]	32.61%
	Ethnicity: Latino	0.49	0.21	3503	2.32	0.021	0.03	[0.00, 0.05]	0.49	[0.09, 0.89]	42.55%
	Age	-0.03	0.00	3405	-9.72	0.000	-0.12	[-0.14, -0.09]	-0.03	[-0.04, -0.03]	100%

[j.] This table reports results of linear mixed models predicting distress as a function of Time, Loneliness, Patient Probability Score (PPS), and Household size, while controlling for demographic categories in 3584 participants with sufficient data. Household size was modeled both within individuals (household size over time) and between individuals (average household size). We report results that were significant at p < .05 (two-sided, no multiplicity correction) for brevity. Practically significant factors are bolded, statistically significant factors of undecided practical significance are italicized, and effects that were consistent with the null hypothesis are reported in plain text. For complete results, including statistics for non-significant factors, see the spreadsheet "Complete_Supplementary_Tables.xls" available at https://osf.io/e7jrd/. Results were evaluated using the following model:

distress ~ Gender + Education + Ethnicity + Racial identity + Setting + Age + Time between*Time within*Loneliness between*Loneliness within*Household size within * Household size between *PPS + (1 + Time within + Loneliness within | SUBJECT_NUMBER)

Table S9. Longitudinal model of loneliness as a function of household size.[k]

		LMER					Standardized		BRMS		
	Term	Estimate	Std. Error	df	t value	Pr(>|t|)	Std. Coef.	95% CI	Median	95% CI	% in ROPE
Main effects	**(Intercept)**	5.51	0.05	3551	114.90	0.000	0.00	[0.00, 0.00]	5.50	[5.41, 5.59]	0%
	**PPS**	0.33	0.01	3548	25.25	0.000	0.41	[0.37, 0.44]	0.34	[0.31, 0.36]	0%
	**Average household size**	-0.28	0.03	3555	-10.27	0.000	-0.16	[-0.19, -0.13]	-0.27	[-0.33, -0.22]	0.11%
	Household size over time	-0.19	0.01	285200	-32.24	0.000	-0.08	[-0.08, -0.07]	-0.19	[-0.20, -0.18]	99.17%
	Duration	-0.02	0.01	1771	-2.16	0.031	-0.03	[-0.06, 0.00]	-0.02	[-0.04, 0.00]	100%
Interactions	Average participation date x Household size over time	-0.05	0.00	283800	-16.64	0.000	-0.04	[-0.05, -0.04]	-0.05	[-0.06, -0.05]	100%
	Average participation date x Duration x Average household size x PPS	0.01	0.00	1897	2.77	0.006	0.04	[0.01, 0.07]	0.01	[0.00, 0.01]	100%
	Duration x Average household size	0.02	0.01	1785	2.10	0.036	0.03	[0.00, 0.06]	0.02	[0.00, 0.03]	100%
	Household size over time x Average household size	0.04	0.00	285200	11.23	0.000	0.03	[0.02, 0.03]	0.04	[0.03, 0.05]	100%
	Household size over time x PPS	-0.03	0.00	285000	-12.90	0.000	-0.03	[-0.04, -0.03]	-0.03	[-0.04, -0.03]	100%
	Average participation date x Household size over time x Average household size	0.04	0.00	276900	12.95	0.000	0.03	[0.03, 0.04]	0.04	[0.03, 0.04]	100%
	Duration x Household size over time x Average household size	0.01	0.00	284600	6.61	0.000	0.02	[0.01, 0.03]	0.01	[0.01, 0.02]	100%
	Duration x Household size over time	0.02	0.00	285000	4.33	0.000	0.01	[0.01, 0.02]	0.02	[0.01, 0.02]	100%
	Duration x Household size over time x PPS	-0.01	0.00	284900	-4.05	0.000	-0.01	[-0.02, -0.01]	-0.01	[-0.01, 0.00]	100%
	Average participation date x Duration x Household size over time x PPS	0.00	0.00	284700	-3.16	0.002	-0.01	[-0.01, 0.00]	0.00	[0.00, 0.00]	100%
	Duration x Household size over time x Average household size x PPS		0.00	285000	2.69	0.007	0.01	[0.00, 0.01]	0.00	[0.00, 0.00]	100%
	Average participation date x Household size over time x Average household size x PPS	0.00	0.00	275900	3.22	0.001	0.01	[0.00, 0.01]	0.00	[0.00, 0.01]	100%
	Household size over time x Average household size x PPS	0.00	0.00	285200	2.64	0.008	0.01	[0.00, 0.01]	0.00	[0.00, 0.01]	100%
	Average participation date x Duration x Household size over time	0.00	0.00	284400	-1.97	0.048	-0.01	[-0.01, 0.00]	0.00	[-0.01, 0.00]	100%
	Education: Less than Bachelors	0.30	0.09	3560	3.57	0.000	0.06	[0.03, 0.09]	0.30	[0.09, 0.51]	13.88%
	Education: Less than advanced degree	0.12	0.06	3547	2.05	0.041	0.03	[0.00, 0.06]	0.13	[0.02, 0.23]	90.52%
	Age	-0.01	0.00	3546	-6.17	0.000	-0.10	[-0.13, -0.07]	-0.01	[-0.02, -0.01]	100%

[k.] This table reports results of linear mixed models predicting loneliness as a function of Time, Loneliness, Patient Probability Score (PPS), and Household size (see Table S8), while controlling for demographic categories in 3588 participants with sufficient data. We report results that were significant at $p < .05$ (two-sided, no multiplicity correction) for brevity. Practically significant factors are bolded, statistically significant factors of undecided practical significance are italicized, and effects that were consistent with the null hypothesis are reported in plain text. For complete results, including statistics for non-significant factors, see the spreadsheet "Complete_Supplementary_Tables.xls" available at https://osf.io/e7jrd/. Results were evaluated using the following model:

Loneliness ~ Gender + Education + Ethnicity + Racial identity + Setting + Age + Time between*Time within*Household size within * Household size between *PPS + (1 + Time within | SUBJECT_NUMBER)

Table S10. Longitudinal model of distress as a function of relationship quality.[l]

		LMER					Standardized		BRMS		
	term	estimate	Std. error	df	t-statistic	p.value	Coefficient (std.)	95% CI	Median	95% CI	% in ROPE
Main effects	**(Intercept)**	6.27	0.09	3475	73.61	0.000	0.00	[0.00, 0.00]	6.27	[6.11, 6.45]	0%
	**PPS**	0.75	0.03	3476	28.55	0.000	0.41	[0.38, 0.43]	0.75	[0.70, 0.80]	0%
	**Average loneliness**	0.82	0.03	3547	25.42	0.000	0.37	[0.34, 0.39]	0.82	[0.76, 0.89]	0%
	**Loneliness over time**	0.50	0.02	1999	25.58	0.000	0.23	[0.21, 0.24]	0.5	[0.46, 0.53]	0.88%
	Relationship quality over time	-0.21	0.01	1965	-14.27	0.000	-0.12	[-0.14, -0.10]	-0.21	[-0.24, -0.18]	100%
	Average relationship quality	-0.28	0.03	3584	-8.21	0.000	-0.12	[-0.15, -0.09]	-0.29	[-0.35, -0.22]	100%
	Average participation date	-0.09	0.02	3599	-3.70	0.000	-0.05	[-0.08, -0.02]	-0.09	[-0.13, -0.04]	100%
	Duration	-0.05	0.01	2738	-3.81	0.000	-0.04	[-0.06, -0.02]	-0.05	[-0.07, -0.02]	100%
Interactions	Average participation date x Duration	-0.03	0.01	2689	-5.20	0.000	-0.06	[-0.08, -0.04]	-0.03	[-0.04, -0.02]	100%
	Average participation date x PPS	-0.03	0.01	3620	-3.04	0.002	-0.04	[-0.07, -0.02]	-0.03	[-0.06, -0.01]	100%
	Duration x PPS	-0.02	0.01	2718	-3.72	0.000	-0.04	[-0.06, -0.02]	-0.02	[-0.03, -0.01]	100%
	Average loneliness x Loneliness over time	0.05	0.01	2526	3.44	0.001	0.03	[0.01, 0.05]	0.05	[0.02, 0.08]	100%
	Average loneliness x Duration	0.02	0.01	2654	2.35	0.019	0.03	[0.00, 0.05]	0.02	[0.00, 0.03]	100%
	Average loneliness x Duration x Average relationship quality	-0.01	0.00	2712	-2.26	0.024	-0.03	[-0.05, 0.00]	-0.01	[-0.02, 0.00]	100%
	Average participation date x Relationship quality over time x PPS	-0.01	0.00	2057	-3.20	0.001	-0.03	[-0.05, -0.01]	-0.01	[-0.02, 0.00]	100%
	Average loneliness x Average participation date x Duration x PPS	0.00	0.00	2612	2.76	0.006	0.03	[0.01, 0.06]	0.00	[0.00, 0.01]	100%
	Average loneliness x Loneliness over time x Average participation date x Duration x Relationship quality over time x Average relationship quality	0.01	0.00	13700	3.09	0.002	0.03	[0.01, 0.05]	0.01	[0.00, 0.01]	100%
	Average participation date x Relationship quality over time	-0.01	0.01	2003	-2.12	0.034	-0.02	[-0.03, 0.00]	-0.01	[-0.03, 0.00]	100%
	Relationship quality over time x PPS	-0.01	0.01	1931	-2.06	0.039	-0.02	[-0.03, 0.00]	-0.01	[-0.03, 0.00]	100%
	Average participation date x Duration x Relationship quality over time	-0.01	0.00	18660	-2.74	0.006	-0.02	[-0.04, -0.01]	-0.01	[-0.02, 0.00]	100%
	Average participation date x Duration x Relationship quality over time x PPS	0.00	0.00	19300	-2.29	0.022	-0.02	[-0.04, 0.00]	0.00	[-0.01, 0.00]	100%
	Loneliness over time x Duration x Average relationship quality x PPS	0.01	0.00	20890	2.11	0.035	0.02	[0.00, 0.04]	0.01	[0.00, 0.01]	100%
	Loneliness over time x Average participation date x Duration x Relationship quality over time x Average relationship quality	-0.01	0.00	14410	-2.05	0.040	-0.02	[-0.04, 0.00]	-0.01	[-0.01, 0.00]	100%

	Loneliness over time x Average participation date x Relationship quality over time x Average relationship quality x PPS	0.00	0.00	20430	-2.16	0.031	-0.02	[-0.04, 0.00]	0.00	[-0.01, 0.00]	100%
Covariates	*Education: Less than Associates*	0.82	0.29	3670	2.85	0.004	0.03	[0.01, 0.06]	0.80	[0.23, 1.35]	10.92%
	Racial identity: AA	-0.63	0.25	3645	-2.49	0.013	-0.03	[-0.05, -0.01]	-0.63	[-1.14, -0.12]	24.04%
	Education: Less than Bachelors	0.53	0.15	3586	3.64	0.000	0.05	[0.02, 0.07]	0.53	[0.25, 0.82]	28.80%
	Gender: Man	-0.49	0.12	3425	-3.94	0.000	-0.04	[-0.07, -0.02]	-0.49	[-0.72, -0.25]	37.92%
	Ethnicity: Latino	0.44	0.21	3482	2.09	0.036	0.03	[0.00, 0.05]	0.43	[0.03, 0.83]	53.75%
	Age	-0.03	0.00	3418	-10.18	0.000	-0.12	[-0.15, -0.10]	-0.03	[-0.04, -0.03]	100%

[I.] This table reports results of linear mixed models predicting distress as a function of Time, Loneliness, Patient Probability Score (PPS), and Relationship quality, while controlling for demographic categories in 3573 participants with sufficient data. Relationship quality was modeled both within individuals (relationship quality over time) and between individuals (average relationship quality). We report results that were significant at $p < .05$ (two-sided, no multiplicity correction) for brevity. Practically significant factors are bolded, statistically significant factors of undecided practical significance are italicized, and effects that were consistent with the null hypothesis are reported in plain text. For complete results, including statistics for non-significant factors, see the spreadsheet "Complete_Supplementary_Tables.xls" available at https://osf.io/e7jrd/. Results were evaluated using the following model:

distress ~ Gender + Education + Ethnicity + Racial identity + Setting + Age + Time between*Time within*Loneliness between*Loneliness within*Relationship quality within * Relationship quality between *PPS + (1 + Time within + Loneliness within + Relationship quality within | SUBJECT_NUMBER)

Table S11. Longitudinal model of loneliness as a function of relationship quality.[m]

		LMER					Standardized		BRMS		
		Estimate	Std. Error	df	t value	Pr(>|t|)	Coefficient (std.)	95% CI	Median	95% CI	% in ROPE
Main effects	**(Intercept)**	5.47	0.04	3536	124.22	0.000	0.00	[0.00, 0.00]	5.47	[5.38, 5.55]	0%
	**Average relationship quality**	-0.43	0.02	3544	-28.41	0.000	-0.42	[-0.45, -0.39]	-0.43	[-0.46, -0.41]	0%
	**PPS**	0.24	0.01	3533	19.24	0.000	0.29	[0.26, 0.32]	0.24	[0.22, 0.27]	0%
	Relationship quality over time	-0.17	0.01	2298	-17.23	0.000	-0.21	[-0.24, -0.19]	-0.17	[-0.19, -0.15]	99.99%
	Duration	-0.02	0.01	1838	-2.95	0.003	-0.04	[-0.07, -0.01]	-0.02	[-0.04, -0.01]	100%
Interactions	Duration x Average relationship quality	-0.02	0.01	1853	-3.59	0.000	-0.05	[-0.08, -0.02]	-0.02	[-0.03, -0.01]	100%
	Duration x Relationship quality over time x Average relationship quality	0.00	0.00	280600	5.02	0.000	0.01	[0.01, 0.02]	0.00	[0.00, 0.01]	100%
	Duration x Relationship quality over time x Average relationship quality x PPS	0.00	0.00	280700	-4.19	0.000	-0.01	[-0.02, -0.01]	0.00	[0.00, 0.00]	100%
	Average participation date x Duration x Relationship quality over time x Average relationship quality x PPS	0.00	0.00	280600	4.34	0.000	0.01	[0.01, 0.01]	0.00	[0.00, 0.00]	100%
	Duration x Relationship quality over time x PPS	0.00	0.00	281000	3.16	0.002	0.01	[0.00, 0.01]	0.00	[0.00, 0.00]	100%
	Average participation date x Duration x Relationship quality over time x PPS	0.00	0.00	280800	-2.69	0.007	-0.01	[-0.01, 0.00]	0.00	[0.00, 0.00]	100%
	Average participation date x Duration x Relationship quality over time	0.00	0.00	280700	-2.31	0.021	-0.01	[-0.01, 0.00]	0.00	[0.00, 0.00]	100%
Covariates	*Education: Less than Bachelors*	0.25	0.08	3549	3.16	0.002	0.05	[0.02, 0.08]	0.25	[0.11, 0.41]	23.08%
	Setting: Urban	0.16	0.05	3528	3.01	0.003	0.04	[0.02, 0.07]	0.17	[0.07, 0.29]	71.08%

[m.] This table reports results of linear mixed models predicting loneliness as a function of Time, Loneliness, Patient Probability Score (PPS), and Relationship quality, while controlling for demographic categories, in 3576 participants with sufficient data. We report results that were significant at $p < .05$ (two-sided, no multiplicity correction) for brevity. Practically significant factors are bolded, statistically significant factors of undecided practical significance are italicized, and effects that were consistent with the null hypothesis are reported in plain text. For complete results, including statistics for non-significant factors, see the spreadsheet “Complete_Supplementary_Tables.xls” available at https://osf.io/e7jrd/. Results were evaluated using the following model:

Loneliness ~ Gender + Education + Ethnicity + Racial identity + Setting + Age + Time between*Time within * Relationship quality between *PPS + (1 + Time within + Relationship quality within | SUBJECT_NUMBER)

Table S12. Longitudinal model of distress as a function of social and emotional support.[n]

		LMER					Standardized		BRMS		
		estimate	std.error	t-statistic	df	p.value	Std. Coef.	95% CI	Median	95% CI	% in ROPE
Main effects	**(Intercept)**	6.30	0.10	2231	60.13	0.000	0.00	[0.00, 0.00]	6.30	[6.09, 6.50]	0%
	**Average loneliness**	0.98	0.05	2202	21.79	0.000	0.44	[0.40, 0.48]	0.99	[0.90, 1.07]	0%
	**PPS**	0.70	0.03	2208	20.36	0.000	0.39	[0.35, 0.43]	0.69	[0.63, 0.76]	0%
	**Loneliness over time**	0.57	0.02	1489	25.11	0.000	0.26	[0.24, 0.28]	0.57	[0.52, 0.61]	0%
	Social Support	0.04	0.01	2199	4.82	0.000	0.09	[0.06, 0.13]	0.04	[0.02, 0.05]	100%
	Emotional Support	-0.04	0.01	2214	-3.88	0.000	-0.08	[-0.12, -0.04]	-0.04	[-0.06, -0.02]	100%
	Duration	-0.03	0.01	2220	-2.36	0.018	-0.03	[-0.05, 0.00]	-0.03	[-0.06, -0.01]	100%
Interactions	Average participation date x Duration	-0.03	0.01	2162	-3.97	0.000	-0.05	[-0.08, -0.03]	-0.03	[-0.04, -0.01]	100%
	Average loneliness x Loneliness over time	0.06	0.02	1935	3.96	0.000	0.04	[0.02, 0.06]	0.06	[0.03, 0.10]	100%
	Duration x Emotional Support	-0.01	0.00	2253	-2.56	0.011	-0.04	[-0.06, -0.01]	-0.01	[-0.01, 0.00]	100%
	Duration x PPS	-0.02	0.01	2219	-3.55	0.000	-0.04	[-0.07, -0.02]	-0.02	[-0.04, -0.01]	100%
	Loneliness over time x PPS	0.03	0.01	1538	2.55	0.011	0.03	[0.01, 0.05]	0.03	[0.01, 0.05]	100%
	Average loneliness x Loneliness over time x Emotional Support x Social Support x PPS	0.00	0.00	2435	2.35	0.019	0.03	[0.01, 0.06]	0.00	[0.00, 0.00]	100%
	Average loneliness x Loneliness over time x PPS	-0.02	0.01	2024	-2.13	0.033	-0.02	[-0.04, 0.00]	-0.02	[-0.03, 0.00]	100%
	Average loneliness x Loneliness over time x Average participation date x PPS	0.01	0.00	2008	1.97	0.049	0.02	[0.00, 0.04]	0.01	[0.00, 0.01]	100%
Covariates	*Ethnicity: unknown*	1.74	0.72	2255	2.42	0.015	0.03	[0.01, 0.06]	1.65	[0.33, 2.98]	3.86%
	Racial identity: AA	-0.89	0.35	2243	-2.51	0.012	-0.04	[-0.06, -0.01]	-0.89	[-1.56, -0.17]	11.28%
	Education: Less than bachelors	0.61	0.19	2219	3.30	0.001	0.05	[0.02, 0.08]	0.61	[0.26, 0.98]	18.17%
	Gender: Man	-0.48	0.15	2205	-3.25	0.001	-0.05	[-0.07, -0.02]	-0.47	[-0.76, -0.19]	41.67%
	Age	-0.04	0.00	2200	-9.27	0.000	-0.13	[-0.16, -0.11]	-0.04	[-0.04, -0.03]	100%

[n.] This table reports results of linear mixed models predicting distress as a function of Time, Loneliness, Patient Probability Score (PPS), Social Support, and Emotional Support, while controlling for demographic categories in 2256 participants with complete data. Social and emotional support were reported at the end of the study, and thus there was one value per subject. We report results that were significant at $p < .05$ (two-sided, no multiplicity correction) for brevity. Practically significant factors are bolded, statistically significant factors of undecided practical significance are italicized, and effects that were consistent with the null hypothesis are reported in plain text. For complete results, including statistics for non-significant factors, see the spreadsheet "Complete_Supplementary_Tables.xls" available at https://osf.io/e7jrd/. Results were evaluated using the following model:

Distress ~ Gender + Education + Ethnicity + Racial identity + Setting + Age + Time between*Time within*Loneliness between*Loneliness within*Social Support * Emotional Support *PPS + (1 + Time within + Loneliness within | SUBJECT_NUMBER)

Table S13. Longitudinal model of loneliness as a function of social and emotional support.[o]

		LMER					Standardized		BRMS		
		Estimate	Std. Error	df	t value	Pr(>|t|)	Coefficient (std.)	95% CI	Median	95% CI	% in ROPE
Main effects	**(Intercept)**	5.50	0.05	2231	100.68	0.000	0.00	[0.00, 0.00]	5.54	[5.45, 5.66]	0%
	**PPS**	0.24	0.02	2222	14.41	0.000	0.30	[0.26, 0.34]	0.23	[0.20, 0.25]	2.08%
	Social Support	-0.04	0.00	2220	-10.71	0.000	-0.23	[-0.27, -0.19]	-0.04	[-0.04, -0.03]	100%
	Emotional Support	-0.07	0.01	2220	-12.94	0.000	-0.29	[-0.33, -0.24]	-0.07	[-0.09, -0.06]	100%
Interactions	Average Participation Date x Duration x Emotional Support x Social Support	0.00	0.00	2140	2.29	0.022	0.03	[0.00, 0.06]	0.00	[0.00, 0.00]	100%
	Average Participation Date x Duration x Emotional Support	0.00	0.00	2129	2.56	0.011	0.04	[0.01, 0.07]	0.00	[0.00, 0.00]	100%
	Duration x Emotional Support	-0.01	0.00	2137	-5.64	0.000	-0.08	[-0.11, -0.05]	-0.01	[-0.01, 0.00]	100%
Covariates	*Racial identity: African-American*	-0.50	0.19	2220	-2.61	0.009	-0.04	[-0.08, -0.01]	-0.44	[-0.79, -0.02]	17.08%
	Ethnicity: missing	0.34	0.17	2224	1.98	0.048	0.03	[0.00, 0.07]	0.36	[-0.08, 0.74]	25.35%
	Gender: Man	-0.19	0.08	2218	-2.39	0.017	-0.04	[-0.07, -0.01]	-0.21	[-0.34, -0.06]	44.22%
	Age	-0.01	0.00	2218	-4.83	0.000	-0.09	[-0.12, -0.05]	-0.01	[-0.02, -0.01]	100%

[o]. This table reports results of linear mixed models predicting loneliness as a function of Time, Patient Probability Score (PPS), Social Support, and Emotional Support, while controlling for demographic categories in 2256 participants with complete data. We report results that were significant at $p < .05$ (two-sided, no multiplicity correction) for brevity. Practically significant factors are bolded, statistically significant factors of undecided practical significance are italicized, and effects that were consistent with the null hypothesis are reported in plain text. For complete results, including statistics for non-significant factors, see the spreadsheet "Complete_Supplementary_Tables.xls" available at https://osf.io/e7jrd/. Results were evaluated using the following model:

Loneliness ~ Gender + Education + Ethnicity + Racial identity + Setting + Age + Time between*Time within* Social Support * Emotional Support *PPS + (1 + Time within | SUBJECT_NUMBER)

Table S14. Longitudinal model of distress as a function of social distancing.[p]

	Social distancing stress (n = 3594)								Social distancing magnitude (n = 3593)								Time with others (n = 3594)							
	LMER			Standardized		BRMS			LMER			Standardized		BRMS			LMER			Standardized		BRMS		
Term	Est.	t value	p-value	Std. Coef.	95% CI	Median	95% CI	% in ROPE	Est.	t value	p-value	Std. Coef.	95% CI	Median	95% CI	% in ROPE	Est.	t value	p-value	Std. Coef.	95% CI	Median	95% CI	% in ROPE
**(Intercept)**	6.11	69.97	0.000	-0.05	[-0.09, -0.01]	6.11	[5.94, 6.28]	0%	6.19	66.24	0.000	-0.02	[-0.06, 0.02]	6.19	[6.01, 6.39]	0%	6.14	67.43	0.000	-0.04	[-0.08, 0.00]	6.14	[5.96, 6.32]	0%
Mean participation date	-0.10	-4.59	0.000	-0.05	[-0.07, -0.03]	-0.11	[-0.15, -0.06]	100%	-0.08	-3.20	0.001	-0.04	[-0.06, -0.01]	-0.08	[-0.12, -0.03]	100%	-0.09	-3.81	0.000	-0.04	[-0.07, -0.02]	-0.09	[-0.13, -0.04]	100%
Duration	-0.04	-3.35	0.001	-0.02	[-0.03, -0.01]	-0.04	[-0.06, -0.02]	100%	-0.02	-2.01	0.044	-0.02	[-0.02, -0.01]	-0.02	[-0.05, 0.00]	100%	-0.03	-2.21	0.027	-0.02	[-0.02, -0.01]	-0.03	[-0.05, 0.00]	100%
Social distancing over time	0.21	18.97	0.000	0.07	[0.06, 0.08]	0.21	[0.19, 0.23]	100%	0.07	3.98	0.000	0.02	[0.01, 0.03]	0.07	[0.04, 0.11]	100%	0.11	10.28	0.000	0.04	[0.03, 0.05]	0.11	[0.09, 0.13]	100%
**Mean social distancing**	0.51	23.55	0.000	0.27	[0.25, 0.30]	0.51	[0.47, 0.56]	0.13%	0.17	4.19	0.000	0.05	[0.02, 0.07]	0.17	[0.09, 0.25]	100%	0.39	13.73	0.000	0.17	[0.14, 0.19]	0.39	[0.34, 0.45]	97.54%
**PPS**	0.95	38.45	0.000	0.45	[0.43, 0.48]	0.95	[0.91, 1.00]	0%	1.09	42.11	0.000	0.52	[0.50, 0.55]	1.09	[1.04, 1.14]	0%	1.07	42.36	0.000	0.51	[0.49, 0.54]	1.07	[1.02, 1.12]	0%
Mean participation date x Duration	-0.03	-4.76	0.000	-0.02	[-0.03, -0.01]	-0.03	[-0.04, -0.02]	100%	-0.03	-5.12	0.000	-0.02	[-0.03, -0.02]	-0.03	[-0.04, -0.02]	100%	-0.03	-4.95	0.000	-0.02	[-0.03, -0.01]	-0.03	[-0.04, -0.02]	100%
Duration x Social distancing over time	-0.01	-2.49	0.013	-0.01	[-0.01, 0.00]	-0.01	[-0.03, 0.00]	100%	*n.s.*								*n.s.*							
Mean participation date x Mean Social distancing	0.02	1.99	0.047	0.02	[0.00, 0.04]	0.02	[0.00, 0.04]	100%	*n.s.*								0.04	3.01	0.003	0.03	[0.01, 0.05]	0.04	[0.01, 0.06]	100%
Duration x Mean social distancing	*n.s.*								0.04	4.92	0.000	0.02	[0.01, 0.03]	0.04	[0.03, 0.06]	100%	*n.s.*							
Social distancing over time x Mean Social distancing	0.04	7.40	0.000	0.03	[0.02, 0.04]	0.04	[0.03, 0.05]	100%	*n.s.*								0.01	2.29	0.022	0.01	[0.00, 0.02]	0.01	[0.00, 0.02]	100%
Mean participation date x PPS	-0.03	-3.06	0.002	-0.04	[-0.06, -0.01]	-0.03	[-0.05, -0.01]	100%	-0.03	-2.43	0.015	-0.03	[-0.05, -0.01]	-0.03	[-0.05, -0.01]	100%	-0.03	-2.93	0.003	-0.04	[-0.06, -0.01]	-0.03	[-0.05, -0.01]	100%
Duration x PPS	-0.02	-4.28	0.000	-0.02	[-0.03, -0.01]	-0.02	[-0.03, -0.01]	100%	-0.02	-3.60	0.000	-0.02	[-0.03, -0.01]	-0.02	[-0.03, -0.01]	100%	-0.02	-3.86	0.000	-0.02	[-0.03, -0.01]	-0.02	[-0.03, -0.01]	100%
Social distancing over time x PPS	*n.s.*								*n.s.*								0.02	3.15	0.002	0.01	[0.01, 0.02]	0.02	[0.01, 0.03]	100%
Mean social distancing x PPS	*n.s.*								0.05	2.74	0.006	0.03	[0.01, 0.06]	0.05	[0.01, 0.08]	100%	*n.s.*							
Mean participation date x Duration x Mean social distancing	*n.s.*								-0.01	-2.28	0.023	-0.01	[-0.02, 0.00]	-0.01	[-0.02, 0.00]	100%	*n.s.*							
Duration x Social distancing over time x PPS	0.01	2.34	0.019	0.01	[0.00, 0.01]	0.01	[0.00, 0.01]	100%	n.s.								*n.s.*							
Mean participation date x Mean social distancing x PPS	0.01	2.14	0.033	0.02	[0.00, 0.04]	0.01	[0.00, 0.02]	100%	*n.s.*								n.s.							

[p.] The table reports terms whose associations were statistically significant based on frequentist models (p < .05, two-sided, no multiplicity correction) for models that measured associations between social distancing, as measured by each individual social distancing measure, and distress, as measured by the Kessler-5[38]. Practically significant factors are bolded, statistically significant factors of undecided practical significance are italicized, and effects that were consistent with the null hypothesis are reported in plain text. Sample sizes for each analysis are included in column headers. The table omits significant covariates, which were largely

consistent with the main longitudinal model (Table 2). For complete results, including statistics for non-significant factors, see the spreadsheet “Complete_Supplementary_Tables.xls” available at https://osf.io/e7jrd/.

Table S15. Longitudinal model of loneliness as a function of social distancing.[q]

	Social distancing stress (n = 3589)								Social distancing magnitude (n = 3588)								Time with others (n = 3589)							
	LMER			Standardized		BRMS			LMER			Standardized		BRMS			LMER			Standardized		BRMS		
Term	Estimate	t value	p-value	Std. Coef.	95% CI	Median	95% CI	% in ROPE	Estimate	t value	p-value	Std. Coef.	95% CI	Median	95% CI	% in ROPE	Estimate	Std. Error	Pr(>|t|)	Coefficient (std.)	95% CI	Median	95% CI	% in ROPE
**(Intercept)**	5.41	122.52	0.000	0.00	[0.00, 0.00]	5.39	[5.31, 5.48]	0%	5.46	112.92	0.000	0.00	[0.00, 0.00]	5.47	[5.36, 5.56]	0%	5.42	117.52	0.000	0.00	[0.00, 0.00]	5.42	[5.31, 5.50]	0%
Duration	-0.02	-2.56	0.011	-0.04	[-0.07, -0.01]	-0.02	[-0.04, 0.00]	100%					*n.s.*								*n.s.*			
Social distancing over time	0.11	12.37	0.000	0.16	[0.14, 0.19]	0.11	[0.09, 0.14]	100%	0.07	5.22	0.000	0.08	[0.05, 0.10]	0.07	[0.05, 0.10]	100%	0.09	9.14	0.000	0.14	[0.11, 0.17]	0.09	[0.07, 0.10]	100%
**Mean social distancing**	0.30	27.80	0.000	0.40	[0.38, 0.43]	0.30	[0.28, 0.32]	0%	0.05	2.25	0.025	0.04	[0.00, 0.07]	0.04	[0.00, 0.09]	100%	0.27	19.21	0.000	0.29	[0.26, 0.31]	0.28	[0.25, 0.30]	0%
**PPS**	0.26	20.88	0.000	0.32	[0.29, 0.34]	0.26	[0.23, 0.28]	0%	0.34	25.68	0.000	0.41	[0.38, 0.45]	0.34	[0.31, 0.37]	0%	0.33	25.83	0.000	0.40	[0.37, 0.43]	0.33	[0.30, 0.35]	0%
Duration x Social distancing over time	-0.01	-15.23	0.000	-0.04	[-0.04, -0.03]	-0.01	[-0.01, -0.01]	100%					*n.s.*				0.00	0.00	0.01	-0.01	[-0.01, 0.00]	0.00	[0.00, 0.00]	100%
Mean participation date x Mean social distancing	0.01	2.80	0.005	0.04	[0.01, 0.07]	0.01	[0.00, 0.02]	100%	0.02	2.02	0.044	0.03	[0.00, 0.06]	0.02	[0.00, 0.04]	100%	0.02	3.59	0.000	0.05	[0.02, 0.08]	0.02	[0.01, 0.03]	100%
Duration x Mean social distancing					*n.s.*				0.03	4.84	0.000	0.07	[0.04, 0.10]	0.03	[0.02, 0.04]	100%					*n.s.*			
Social distancing over time x Mean social distancing	0.01	3.66	0.000	0.04	[0.02, 0.07]	0.02	[0.01, 0.02]	100%					*n.s.*								*n.s.*			
Mean social distancing x PPS					*n.s.*				0.02	2.80	0.005	0.04	[0.01, 0.07]	0.03	[0.01, 0.04]	100%					*n.s.*			
Mean participation date x Duration x Social distancing over time					*n.s.*				0.00	4.75	0.000	0.01	[0.01, 0.02]	0.00	[0.00, 0.01]	100%	0.00	0.00	0.00	0.02	[0.01, 0.03]	0.00	[0.00, 0.00]	100%
Mean participation date x Duration x Mean social distancing					*n.s.*				-0.01	-2.56	0.011	-0.04	[-0.07, -0.01]	-0.01	[-0.01, 0.00]	100%					*n.s.*			
Duration x Social distancing over time x Mean social distancing	0.00	-2.18	0.030	-0.01	[-0.01, 0.00]	0.00	[0.00, 0.00]	100%	0.00	2.50	0.012	0.01	[0.00, 0.01]	0.00	[0.00, 0.00]	100%					*n.s.*			
Duration x Social distancing over time x PPS	0.00	4.51	0.000	0.01	[0.01, 0.02]	0.00	[0.00, 0.00]	100%	-0.01	-6.87	0.000	-0.02	[-0.03, -0.02]	-0.01	[-0.01, 0.00]	100%	0.00	-6.25	0.000	-0.02	[-0.03, -0.01]	0.00	[0.00, 0.00]	100%
Mean participation date x Duration x Social distancing over time x Mean social distancing	0.00	-2.25	0.025	-0.01	[-0.01, 0.00]	0.00	[0.00, 0.00]	100%					*n.s.*								*n.s.*			
Mean participation date x Duration x Social distancing over time x PPS	0.00	-5.46	0.000	-0.01	[-0.02, -0.01]	0.00	[0.00, 0.00]	100%					*n.s.*								*n.s.*			
Mean participation date x Duration x Mean social distancing x PPS					*n.s.*				0.00	-2.29	0.022	-0.03	[-0.06, 0.00]	0.00	[-0.01, 0.00]	100%					*n.s.*			
Duration x Social distancing over time x Mean social distancing x PPS	0.00	4.96	0.000	0.01	[0.01, 0.02]	0.00	[0.00, 0.00]	100%					*n.s.*				0.00	-4.64	0.000	-0.02	[-0.02, -0.01]	0.00	[0.00, 0.00]	100%
Mean participation date x Duration x Social distancing over time x Mean social distancing x PPS	0.00	-4.39	0.000	-0.01	[-0.02, -0.01]	0.00	[0.00, 0.00]	100%	0.00	-2.60	0.009	-0.01	[-0.01, 0.00]	0.00	[0.00, 0.00]	100%	0.00	-2.94	0.003	-0.01	[-0.02, 0.00]	0.00	[0.00, 0.00]	100%

[q.] The table reports terms whose associations were statistically significant based on frequentist models ($p < .05$, two-sided, no multiplicity correction) for models that measured associations between social distancing, as measured by each individual social distancing measure, and loneliness. Sample sizes are included in column headers. Practically significant factors are bolded, statistically significant factors of undecided practical significance are italicized, and effects that were consistent with the null hypothesis are reported in plain text. The table omits significant covariates, which were largely consistent with the main longitudinal model (Table 2). For complete results, including statistics for non-significant factors, see the spreadsheet "Complete_Supplementary_Tables.xls" available at https://osf.io/e7jrd/.

Table S16. Longitudinal model of distress as a function of social distancing, age, and gender.[r]

	LMER					Standardized	
	Estimate	Std. Error	Df	t value	Pr(>|t|)	Std. Coef.	95% CI
(Intercept)	6.25	0.06	3470	113.52	0.000	0.00	[0.00, 0.00]
Age	-0.04	0.00	3495	-11.46	0.000	-0.16	[-0.19, -0.13]
Gender	-0.10	0.03	3558	-3.96	0.000	-0.06	[-0.09, -0.03]
Mean participation date	-0.03	0.01	2706	-2.30	0.021	-0.02	[-0.05, 0.00]
Duration	0.99	0.03	3440	38.53	0.000	0.53	[0.51, 0.56]
PPS	0.13	0.04	3489	3.02	0.003	0.05	[0.02, 0.07]
Mean social distancing	0.19	0.01	2247	17.11	0.000	0.14	[0.12, 0.15]
Social distancing stress over time	0.52	0.03	3550	19.28	0.000	0.31	[0.28, 0.34]
Mean social distancing stress	-0.10	0.03	3558	-3.96	0.000	-0.06	[-0.09, -0.03]
Time with others over time	-0.03	0.01	2706	-2.30	0.021	-0.02	[-0.05, 0.00]
Age x Mean participation date	0.00	0.00	3566	2.31	0.021	0.04	[0.01, 0.07]
Age x Duration	0.00	0.00	2711	3.32	0.001	0.04	[0.02, 0.06]
Gender x Duration	-0.08	0.02	2889	-4.04	0.000	-0.05	[-0.07, -0.02]
Mean participation date x Duration	-0.03	0.01	2573	-4.21	0.000	-0.05	[-0.07, -0.03]
Age x PPS	-0.01	0.00	3480	-3.30	0.001	-0.05	[-0.08, -0.02]
Duration x PPS	-0.03	0.01	2673	-4.65	0.000	-0.05	[-0.07, -0.03]
Social distancing stress over time x Mean social distancing stress	0.04	0.01	2648	6.27	0.000	0.05	[0.03, 0.07]
Age x Social distancing stress over time	0.00	0.00	2216	2.22	0.027	0.02	[0.00, 0.03]
Age x Time with others over time	0.00	0.00	1652	-3.11	0.002	-0.03	[-0.04, -0.01]
Gender x Time with others over time	0.03	0.02	1752	2.13	0.034	0.02	[0.00, 0.04]
Gender x Mean time with others	0.10	0.05	3539	2.06	0.040	0.04	[0.00, 0.07]
Duration x Mean social distancing	0.03	0.01	2792	3.13	0.002	0.03	[0.01, 0.06]
Duration x Social distancing stress over time	-0.02	0.01	19550	-3.22	0.001	-0.03	[-0.04, -0.01]
Duration x Time with others over time	0.02	0.01	9578	2.40	0.016	0.02	[0.00, 0.04]
PPS x Mean social distancing	0.06	0.02	3440	3.02	0.003	0.05	[0.02, 0.07]
Age x Mean participation date x Duration	0.00	0.00	2618	2.15	0.031	0.03	[0.00, 0.05]
Age x Duration x PPS	0.00	0.00	2691	-2.07	0.038	-0.02	[-0.05, 0.00]
Gender x Time with others over time x Mean time with others	0.02	0.01	1633	2.22	0.027	0.02	[0.00, 0.04]
Age x Duration x Social distancing over time	0.00	0.00	11810	2.22	0.027	0.02	[0.00, 0.04]
Gender x Duration x Mean social distancing stress	-0.02	0.01	2667	-2.01	0.044	-0.03	[-0.06, 0.00]
Mean participation date x Duration x Mean social distancing	-0.02	0.01	2561	-3.03	0.003	-0.04	[-0.06, -0.01]
Mean participation date x PPS x Time with others over time	0.01	0.00	2049	2.51	0.012	0.02	[0.00, 0.04]
Age x Mean participation date x Duration x PPS	0.00	0.00	2634	2.28	0.023	0.03	[0.00, 0.06]
Gender x Mean participation date x Time with others over time x Mean time with others	-0.01	0.00	1813	-3.38	0.001	-0.04	[-0.06, -0.01]
Age x Mean participation date x Duration x Time with others over time	0.00	0.00	13760	-1.96	0.050	-0.02	[-0.04, 0.00]

Gender x Mean participation date x PPS x Mean time with others	-0.02	0.01	3659	-1.97	0.049	-0.04	[-0.08, 0.00]
Mean participation date x Duration x PPS x Mean social distancing stress	0.00	0.00	2623	2.33	0.020	0.03	[0.01, 0.06]
Age x Mean participation date x Duration x Social distancing over time x Mean social distancing	0.00	0.00	11280	-2.88	0.004	-0.03	[-0.05, -0.01]
Age x Gender x Mean participation date x Duration x Social distancing over time	0.00	0.00	13500	2.45	0.014	0.03	[0.01, 0.06]
Gender x Mean participation date x PPS x Time with others over time x Mean time with others	-0.01	0.00	1822	-3.30	0.001	-0.04	[-0.06, -0.01]
Age x Gender x Duration x PPS x Time with others over time	0.00	0.00	10520	2.04	0.041	0.02	[0.00, 0.05]
Age x Mean participation date x Duration x PPS x Time with others over time	0.00	0.00	12890	-2.12	0.034	-0.02	[-0.04, 0.00]
Age x Mean participation date x Duration x PPS x Social distancing over time x Mean social distancing	0.00	0.00	9625	-2.02	0.043	-0.02	[-0.04, 0.00]

r. This table reports results of linear mixed models predicting psychological distress (measured by the Kessler-5[38]) as a function of Time, Patient Probability Score (PPS), Age, Gender, and Social Distancing as measured by all three self-report measures. The model is equivalent to Extended Data Table 3 in the main manuscript, but tests for interactions with age and gender rather than modeling factors as demographic covariates. 3593 participants had sufficient data to be included in the model. We did not evaluate Bayesian models due to the large number of factors. We evaluated the following model and report results that were significant at $p < .05$ (two-sided, no multiplicity correction) for brevity, although we include the main effect of Gender for completeness. For complete results, including statistics for non-significant factors, see the spreadsheet “Complete_Supplementary_Tables.xls” available at https://osf.io/e7jrd/. The complete model was:

> distress ~ Age*Gender*Time between*Time within*Social distancing within*Social Distancing between-subjects*PPS + Age*Gender*Time between*Time within*Social Distancing Stress within-subjects*Social Distancing Stress between-subjects*PPS+ Age*Gender*Time between*Time within*Time with others within-subjects*Time with others between*PPS+ (1 + Time within + Social distancing within+Social Distancing Stress within-subjects+Time with others within-subjects| SUBJECT_NUMBER)

Table S17. Longitudinal model of loneliness as a function of social distancing, age, and gender.[s]

	LMER					Standardized	
	Estimate	Std. Error	df	t value	Pr(>|t|)	Std. Coef.	95% CI
(Intercept)	5.58	0.03	3521	204.56	0.000	0.00	[0.00, 0.00]
Age	-0.01	0.00	3518	-3.68	0.000	-0.06	[-0.09, -0.03]
Gender	-0.16	0.04	3528	-4.11	0.000	-0.07	[-0.10, -0.03]
PPS	0.27	0.01	3518	21.31	0.000	0.33	[0.30, 0.36]
Social distancing over time	0.04	0.02	2136	2.45	0.014	0.05	[0.01, 0.08]
Social distancing stress over time	0.09	0.01	2193	8.95	0.000	0.14	[0.11, 0.17]
Mean social distancing stress	0.26	0.01	3523	19.40	0.000	0.35	[0.31, 0.38]
Time with others over time	0.06	0.01	2140	5.46	0.000	0.10	[0.06, 0.13]
Mean time with others	0.10	0.02	3531	5.85	0.000	0.11	[0.07, 0.14]
Age x Gender	0.01	0.00	3521	2.36	0.018	0.04	[0.01, 0.07]
Age x Mean participation date	0.00	0.00	3520	2.09	0.037	0.04	[0.00, 0.07]
Age x Mean social distancing stress	0.00	0.00	3520	1.97	0.049	0.04	[0.00, 0.07]
Gender x Mean social distancing stress	0.04	0.02	3520	2.31	0.021	0.05	[0.01, 0.09]
Duration x Social distancing stress over time	-0.01	0.00	284300	-16.37	0.000	-0.04	[-0.05, -0.04]
Duration x Mean social distancing stress	0.01	0.00	2187	2.03	0.043	0.04	[0.00, 0.08]
PPS x Mean social distancing	0.03	0.01	3505	2.97	0.003	0.05	[0.02, 0.08]
Age x Mean participation date x Mean social distancing	0.00	0.00	3512	-2.04	0.041	-0.04	[-0.07, 0.00]
Duration x Social distancing over time x Mean social distancing	0.00	0.00	284100	-4.35	0.000	-0.01	[-0.02, -0.01]
Duration x Social distancing stress over time x Mean social distancing stress	0.00	0.00	282500	-6.99	0.000	-0.02	[-0.02, -0.01]
Duration x Time with others over time x Mean time with others	0.00	0.00	282800	-2.13	0.034	-0.01	[-0.01, 0.00]
Age x Duration x Social distancing stress over time	0.00	0.00	284600	8.64	0.000	0.02	[0.02, 0.03]
Age x Duration x Time with others over time	0.00	0.00	282400	3.63	0.000	0.01	[0.01, 0.02]
Gender x Duration x Social distancing over time	0.02	0.00	280300	7.53	0.000	0.03	[0.02, 0.03]
Gender x Duration x Social distancing stress over time	0.00	0.00	283000	-2.84	0.005	-0.01	[-0.01, 0.00]
Gender x Duration x Time with others over time	0.01	0.00	282000	4.10	0.000	0.02	[0.01, 0.02]
Mean participation date x Duration x Social distancing over time	0.00	0.00	281900	2.33	0.020	0.01	[0.00, 0.01]
Mean participation date x Duration x Time with others over time	0.00	0.00	282000	7.91	0.000	0.03	[0.02, 0.03]
Duration x PPS x Social distancing over time	0.00	0.00	281500	-4.28	0.000	-0.01	[-0.02, -0.01]
Duration x PPS x Social distancing stress over time	0.00	0.00	284600	3.55	0.000	0.01	[0.00, 0.01]
Duration x PPS x Time with others over time	0.00	0.00	281800	-5.26	0.000	-0.02	[-0.02, -0.01]
Gender x Duration x Social distancing stress over time x Mean social distancing stress	0.00	0.00	281800	-2.62	0.009	-0.01	[-0.01, 0.00]
Mean participation date x Duration x Social distancing over time x Mean social distancing	0.00	0.00	283800	-5.47	0.000	-0.02	[-0.03, -0.01]
Age x Mean participation date x Duration x Social distancing over time	0.00	0.00	282000	4.95	0.000	0.02	[0.01, 0.02]
Age x Mean participation date x Duration x Social distancing stress over time	0.00	0.00	284600	4.15	0.000	0.01	[0.01, 0.02]
Age x Mean participation date x Duration x Time with others over time	0.00	0.00	283000	-9.66	0.000	-0.03	[-0.04, -0.03]
Gender x Mean participation date x Duration x Social distancing over time	-0.01	0.00	282000	-6.20	0.000	-0.02	[-0.03, -0.02]
Gender x Mean participation date x Duration x Social distancing stress over time	0.00	0.00	283500	6.70	0.000	0.02	[0.02, 0.03]
Gender x Mean participation date x Duration x Time with others over time	-0.01	0.00	282300	-9.13	0.000	-0.04	[-0.04, -0.03]
Age x Mean participation date x PPS x Time with others over time	0.00	0.00	2204	2.39	0.017	0.05	[0.01, 0.08]
Duration x PPS x Social distancing stress over time x Mean social distancing stress	0.00	0.00	282200	3.46	0.001	0.01	[0.00, 0.02]
Duration x PPS x Time with others over time x Mean time with others	0.00	0.00	282700	-5.37	0.000	-0.02	[-0.03, -0.01]

Age x Duration x PPS x Mean social distancing	0.00	0.00	2219	-2.39	0.017	-0.04	[-0.07, -0.01]
Gender x Duration x PPS x Mean social distancing	0.00	0.00	281400	3.40	0.001	0.01	[0.01, 0.02]
Gender x Duration x PPS x Social distancing over time	-0.01	0.00	284100	-9.26	0.000	-0.03	[-0.03, -0.02]
Gender x Duration x PPS x Social distancing stress over time	0.00	0.00	282800	3.63	0.000	0.01	[0.01, 0.02]
Gender x Duration x PPS x Time with others over time	0.00	0.00	284400	-2.23	0.026	-0.01	[-0.01, 0.00]
Mean participation date x Duration x PPS x Social distancing over time	0.00	0.00	282200	3.46	0.001	0.01	[0.00, 0.02]
Age x Gender x Duration x Social distancing over time x Mean social distancing	0.00	0.00	280100	-3.86	0.000	-0.02	[-0.02, -0.01]
Age x Gender x Duration x Social distancing stress over time x Mean social distancing stress	0.00	0.00	282700	2.41	0.016	0.01	[0.00, 0.01]
Age x Mean participation date x Duration x Social distancing over time x Mean social distancing	0.00	0.00	284200	-9.15	0.000	-0.03	[-0.04, -0.03]
Age x Mean participation date x Duration x Time with others over time x Mean time with others	0.00	0.00	283000	-4.84	0.000	-0.02	[-0.02, -0.01]
Gender x Mean participation date x Duration x Social distancing over time x Mean social distancing	0.00	0.00	283000	-2.93	0.003	-0.01	[-0.02, 0.00]
Gender x Mean participation date x Duration x Social distancing stress over time x Mean social distancing stress	0.00	0.00	283000	-5.93	0.000	-0.02	[-0.03, -0.01]
Age x Gender x Mean participation date x Duration x Social distancing over time	0.00	0.00	280700	3.44	0.001	0.02	[0.01, 0.02]
Age x Gender x Mean participation date x Duration x Time with others over time	0.00	0.00	283100	7.59	0.000	0.03	[0.02, 0.04]
Age x Mean participation date x PPS x Time with others over time x Mean time with others	0.00	0.00	2258	2.81	0.005	0.06	[0.02, 0.10]
Age x Duration x PPS x Social distancing stress over time x Mean social distancing stress	0.00	0.00	280000	-8.79	0.000	-0.03	[-0.03, -0.02]
Gender x Duration x PPS x Social distancing over time x Mean social distancing	0.00	0.00	282100	5.03	0.000	0.02	[0.01, 0.03]
Age x Gender x Duration x PPS x Social distancing over time	0.00	0.00	279500	-4.49	0.000	-0.02	[-0.03, -0.01]
Age x Gender x Duration x PPS x Social distancing stress over time	0.00	0.00	284200	4.04	0.000	0.01	[0.01, 0.02]
Age x Gender x Duration x PPS x Time with others over time	0.00	0.00	283000	-3.65	0.000	-0.01	[-0.02, -0.01]
Mean participation date x Duration x PPS x Social distancing stress over time x Mean social distancing stress	0.00	0.00	283800	-3.94	0.000	-0.01	[-0.02, -0.01]
Mean participation date x Duration x PPS x Time with others over time x Mean time with others	0.00	0.00	281300	-2.29	0.022	-0.01	[-0.02, 0.00]
Age x Mean participation date x Duration x PPS x Social distancing over time	0.00	0.00	277100	-7.74	0.000	-0.03	[-0.04, -0.02]
Age x Mean participation date x Duration x PPS x Social distancing stress over time	0.00	0.00	283700	6.80	0.000	0.02	[0.02, 0.03]
Age x Mean participation date x Duration x PPS x Time with others over time	0.00	0.00	282000	6.05	0.000	0.02	[0.02, 0.03]
Gender x Mean participation date x Duration x PPS x Social distancing stress over time	0.00	0.00	283500	5.85	0.000	0.02	[0.01, 0.03]
Gender x Mean participation date x Duration x PPS x Time with others over time	0.00	0.00	283900	-12.43	0.000	-0.05	[-0.06, -0.04]
Age x Gender x Mean participation date x Duration x Social distancing stress over time x Mean social distancing stress	0.00	0.00	282400	5.96	0.000	0.03	[0.02, 0.04]
Age x Gender x Mean participation date x Duration x Time with others over time x Mean time with others	0.00	0.00	283300	3.30	0.001	0.02	[0.01, 0.03]
Age x Gender x Duration x PPS x Social distancing over time x Mean social distancing	0.00	0.00	282400	-4.31	0.000	-0.02	[-0.03, -0.01]
Age x Gender x Duration x PPS x Time with others over time x Mean time with others	0.00	0.00	283500	-2.50	0.013	-0.01	[-0.02, 0.00]
Age x Mean participation date x Duration x PPS x Social distancing over time x Mean social distancing	0.00	0.00	280100	-5.32	0.000	-0.02	[-0.03, -0.01]
Age x Mean participation date x Duration x PPS x Social distancing stress over time x Mean social distancing stress	0.00	0.00	283200	7.77	0.000	0.03	[0.02, 0.03]
Age x Mean participation date x Duration x PPS x Time with others over time x Mean time with others	0.00	0.00	281100	2.24	0.025	0.01	[0.00, 0.01]
Gender x Mean participation date x Duration x PPS x Social distancing stress over time x Mean social distancing stress	0.00	0.00	283000	-4.34	0.000	-0.02	[-0.02, -0.01]
Gender x Mean participation date x Duration x PPS x Time with others over time x Mean time with others	0.00	0.00	283700	-8.96	0.000	-0.04	[-0.04, -0.03]
Age x Gender x Mean participation date x Duration x PPS x Social distancing over time	0.00	0.00	276900	-2.28	0.022	-0.01	[-0.02, 0.00]
Age x Gender x Mean participation date x Duration x PPS x Social distancing stress over time	0.00	0.00	283900	-2.92	0.003	-0.01	[-0.02, 0.00]
Age x Gender x Mean participation date x Duration x PPS x Time with others over time	0.00	0.00	283000	11.23	0.000	0.05	[0.04, 0.06]
Age x Gender x Mean participation date x Duration x PPS x Social distancing stress over time x Mean social distancing stress	0.00	0.00	283400	2.59	0.010	0.01	[0.00, 0.02]

Age x Gender x Mean participation date x Duration x PPS x Time with others over time x Mean time with others	0.00	0.00	283300	8.28	0.000	0.04	[0.03, 0.05]

s. This table reports results of linear mixed models predicting loneliness as a function of Time, Patient Probability Score (PPS), Age, Gender, and Social Distancing as measured by all three self-report measures in 3588 participants with sufficient data. The model is equivalent to Extended Data Table 4 in the main manuscript, but tests for interactions with age and gender rather than modeling factors as demographic covariates. We did not evaluate Bayesian models due to the large number of factors. We evaluated the following model and report results that were significant at p < .05 (two-sided, no multiplicity correction) for brevity. For complete results, including statistics for non-significant factors, see the spreadsheet “Complete_Supplementary_Tables.xls” available at https://osf.io/e7jrd/. The complete model was:

distress ~ Age*Gender*Time between*Time within*Social distancing within*Social Distancing between-subjects*PPS + Age*Gender*Time between*Time within*Social Distancing Stress within-subjects*Social Distancing Stress between-subjects*PPS+ Age*Gender*Time between*Time within*Time with others within-subjects*Time with others between*PPS+ (1 + Time within + Social distancing within+Social Distancing Stress within-subjects+Time with others within-subjects| SUBJECT_NUMBER)

Table S18. Longitudinal model of distress as a function of community-based social distancing.[t]

		LMER					Standardized		BRMS		
		Estimate	Std. Error	df	t value	P-value	Std. Coef.	95% CI	Median	95% CI	% in ROPE
Main effects	**(Intercept)**	6.22	0.10	3452	63.69	0.000	0.00	[0.00, 0.00]	6.22	[6.03, 6.41]	0%
	**PPS**	1.08	0.03	3468	39.31	0.000	0.58	[0.55, 0.61]	1.08	[1.02, 1.13]	0%
	Mean participation date	-0.10	0.02	3474	-4.13	0.000	-0.06	[-0.09, -0.03]	-0.1	[-0.15, -0.05]	100%
	Duration	-0.03	0.01	2825	-2.16	0.031	-0.02	[-0.05, 0.00]	-0.03	[-0.05, 0.00]	100%
	Regional distancing over time	-0.1	0.03	3393	-3.67	0	-0.06	[-0.09, -0.03]	-0.1	[-0.15, -0.05]	100%
	Mean regional distancing	-0.03	0.01	2738	-2.36	0.018	-0.03	[-0.05, 0.00]	-0.03	[-0.06, -0.01]	100%
Interactions	*Duration x Regional distancing over time*	0.35	0.10	6597	3.40	0.001	0.03	[0.01, 0.05]	0.35	[0.15, 0.55]	83.82%
	Mean participation date x Duration	-0.03	0.01	2683	-5.06	0.000	-0.06	[-0.08, -0.04]	-0.03	[-0.04, -0.02]	100%
	Mean participation date x Duration x Regional distancing over time	-0.17	0.05	3986	-3.75	0.000	-0.04	[-0.05, -0.02]	-0.17	[-0.26, -0.09]	100%
	PPS x Duration	-0.02	0.01	2837	-2.54	0.011	-0.03	[-0.05, -0.01]	-0.02	[-0.03, 0.00]	100%
Covariates	**Education: less than associates**	1.36	0.35	3592	3.90	0.000	0.05	[0.03, 0.08]	1.36	[0.67, 2.03]	0.47%
	**Education: less than bachelors**	0.82	0.17	3499	4.79	0.000	0.07	[0.04, 0.10]	0.82	[0.49, 1.14]	1.40%
	Ethnicity: unknown	1.18	0.58	3853	2.02	0.043	0.03	[0.00, 0.06]	1.12	[-0.02, 2.25]	11.49%
	Racial identity: African American	-0.62	0.30	3480	-2.09	0.037	-0.03	[-0.05, 0.00]	-0.6	[-1.21, -0.02]	29.89%
	Gender: Man	-0.51	0.14	3279	-3.58	0.000	-0.05	[-0.07, -0.02]	-0.51	[-0.79, -0.23]	32.84%
	Age	-0.04	0.00	3296	-12.02	0.000	-0.17	[-0.19, -0.14]	-0.04	[-0.05, -0.04]	100%

[t.] This table reports results of linear mixed models predicting distress as a function of Regional Distancing (based on regional cell phone mobility data[49] within 3420 US participants; higher values = less mobility / more distancing), Time, and Patient Probability Score (PPS), while controlling for demographic categories. We report results that were significant at $p < .05$ (two-sided, no multiplicity correction) for brevity. Practically significant factors are bolded, statistically significant factors of undecided practical significance are italicized, and effects that were consistent with the null hypothesis are reported in plain text. For complete results, including statistics for non-significant factors, see the spreadsheet "Complete_Supplementary_Tables.xls" at https://osf.io/e7jrd/. Results were evaluated using the following model:

distress ~ Gender + Education + Ethnicity + Racial identity + Setting + Age + Time between*Time within*Regional distancing within* Regional distancing between*PPS + (1 + Time within + Regional distancing within| SUBJECT_NUMBER)

Table S19. Reverse mediation models.[u]

	Result	Statistic	Path *a*	Path *b*	Path *c′*	Path *c*	Path a*b
Across participants (i.e., single level mediation)	With Moderators: Controlling for Moderators	Coeff	0.64	0.25	0.67	0.84	0.16
		CI	[0.6, 0.69]	[0.2, 0.3]	[0.6, 0.74]	[0.77, 0.91]	[0.11, 0.22]
		p	< 0.001	< 0.001	< 0.001	<0.001	<0.001
	With Moderators: Moderation by Living Alone	Coeff	0.16	-0.05	-0.02	-0.02	N/A
		CI	[0.06, 0.26]	[-0.17, 0.08]	[-0.2, 0.15]	[-0.18, 0.14]	
		p	*n.s.*	*n.s.*	*n.s.*	*n.s.*	
	With Moderators: Moderation by PPS	Coeff	-0.07	-0.01	0.07	0.04	
		CI	[-0.1, -0.04]	[-0.04, 0.01]	[0.04, 0.11]	[0.02, 0.07]	
		P	< 0.001	*n.s*	< 0.001	*n.s.*	
	With Moderators: Moderation by PPS x Living Alone	Coeff	0.06	-0.01	0.02	0.02	
		CI	[-0.01, 0.13]	[-0.07, 0.04]	[-0.06, 0.09]	[-0.05, 0.09]	
		p	*n.s.*	*n.s.*	*n.s.*	*n.s.*	
Within participants (i.e., multilevel mediation)	With Moderators: First level	Coeff	0.23	0.15	0.50	0.54	0.03
		CI	[0.21, 0.26]	[0.13, 0.17]	[0.47, 0.54]	[0.51, 0.58]	[0.03, 0.04]
		p	< 0.001	<0.001	<0.001	<0.001	N/A
	With Moderators: Second Level moderation by Living Alone	Coeff	0.06	0.02	0.08	0.1	
		CI	[0.01, 0.11]	[-0.03, 0.06]	[0.01, 0.15]	[0.03, 0.17]	
		p	*n.s*	*n.s.*	*n.s.*	*n.s.*	
	With Moderators: Second Level moderation by PPS	Coeff	-0.01	0.01	0.04	0.04	
		CI	[-0.02, 0.00]	[0.00, 0.02]	[0.02, 0. 06]	[0.02, 0.06]	
		p	*n.s.*	*n.s.*	< 0.001	< 0.001	
	With Moderators: Second Level moderation by PPS x Living Alone	Coeff	0.01	-0.02	0.01	0.02	
		CI	[-0.01, 0.04]	[-0.04, 0.00]	[-0.02, 0.05]	[-0.02, 0.05]	
		p	*n.s.*	*n.s*	*n.s.*	*n.s.*	

[u.] This table reports results of mediation models evaluating whether social distancing-related stress mediates associations between loneliness (X) and distress (Y), i.e. reversing the input variable and mediator relative to the models reported in the main manuscript. For each approach, Path a evaluates associations between loneliness and social distancing-related stress. Path b evaluates associations between distancing-related stress and distress while controlling for loneliness. Path c evaluates associations between loneliness and psychological distress without controlling for social distancing-related stress, while c` evaluates relationships when controlling for social distancing-related stress. Path a*b evaluates the overall mediation effect, or indirect pathway through social distancing-related stress; we did not evaluate moderation of Path a*b in this analysis due to its exploratory nature. All factors except Psychological Distress were centered in all models. Hypothesis tests were conducted using “hypothesis” within brms, and p-values are two-sided without multiplicity correction.

Table S20. Longitudinal associations with psychological distress: Complete results.[v]

	LMER					Standardized		BRMS		
	Estimate	Std. Error	df	t value	P-value	Std. Coef.	95% CI	Median	95% CI	% in ROPE
(Intercept)	6.29	0.08	3477	75.04	0.000	0.00	[0.00, 0.00]	6.29	[6.12, 6.46]	0%
gender: male	-0.48	0.12	3455	-3.89	0.000	-0.04	[-0.07, -0.02]	-0.49	[-0.72, -0.23]	38.43%
gender: nonconforming	0.34	0.43	3667	0.80	0.424	0.01	[-0.01, 0.03]	0.33	[-0.52, 1.16]	56.62%
gender: trans	0.99	0.88	3188	1.12	0.261	0.01	[-0.01, 0.03]	0.87	[-0.77, 2.48]	25.12%
gender: other	-0.29	0.77	3574	-0.38	0.701	0.00	[-0.03, 0.02]	-0.28	[-1.70, 1.13]	42.69%
gender: missing	0.29	0.43	3535	0.68	0.500	0.01	[-0.01, 0.03]	0.28	[-0.52, 1.09]	62.24%
education: less than advanced	-0.01	0.10	3436	-0.10	0.923	0.00	[-0.02, 0.02]	-2.77E-03	[-0.22, 0.20]	100%
education: less than BS	0.57	0.15	3610	3.84	0.000	0.05	[0.02, 0.07]	0.57	[0.28, 0.85]	19.98%
education: less than AA	0.84	0.29	3698	2.91	0.004	0.03	[0.01, 0.06]	0.84	[0.28, 1.39]	8.38%
education: missing	-0.55	0.87	3521	-0.63	0.531	-0.01	[-0.03, 0.02]	-0.48	[-2.09, 1.08]	36.38%
ethnicity: Latino	0.46	0.21	3508	2.17	0.030	0.03	[0.00, 0.05]	0.46	[0.05, 0.88]	48.65%
ethnicity: unknown	0.89	0.51	4047	1.75	0.080	0.02	[0.00, 0.04]	0.86	[-0.11, 1.85]	19.78%
ethnicity: missing	-0.13	0.26	3547	-0.51	0.611	-0.01	[-0.03, 0.02]	-0.13	[-0.65, 0.39]	87.58%
racial identity: AA	-0.62	0.26	3685	-2.40	0.017	-0.03	[-0.05, -0.01]	-0.61	[-1.12, -0.11]	26.24%
racial identity: AAPI	-0.53	0.27	3479	-1.98	0.048	-0.02	[-0.05, 0.00]	-0.52	[-1.05, 0.00]	39.75%
racial identity: AI	-0.22	0.49	3793	-0.45	0.656	-0.01	[-0.03, 0.02]	-0.23	[-1.16, 0.71]	60.14%
racial identity: multiple	0.28	0.35	3387	0.79	0.430	0.01	[-0.01, 0.03]	0.27	[-0.42, 0.97]	67.38%
racial identity: unknown	0.03	0.78	3603	0.04	0.969	0.00	[-0.02, 0.02]	0.02	[-1.45, 1.45]	45.86%
racial identity: missing	0.53	0.45	3478	1.19	0.234	0.01	[-0.01, 0.04]	0.51	[-0.37, 1.42]	42.76%
setting: urban	-0.05	0.10	3427	-0.50	0.621	-0.01	[-0.03, 0.02]	-0.05	[-0.25, 0.16]	100%
setting: rural	0.15	0.14	3573	1.04	0.297	0.01	[-0.01, 0.04]	0.15	[-0.12, 0.41]	98.64%
setting: missing	-0.26	0.76	3654	-0.34	0.733	0.00	[-0.03, 0.02]	-0.25	[-1.64, 1.18]	43.75%
age	-0.04	0.00	3427	-11.30	0.000	-0.13	[-0.16, -0.11]	-0.04	[-0.04, -0.03]	100%
PPS	0.77	0.02	3471	31.10	0.000	0.42	[0.39, 0.44]	0.77	[0.72, 0.82]	0%
mean participation date	-0.07	0.02	3606	-3.49	0.000	-0.04	[-0.07, -0.02]	-0.07	[-0.11, -0.03]	100%
duration	-0.03	0.01	2636	-2.80	0.005	-0.02	[-0.04, -0.01]	-0.03	[-0.05, -0.01]	100%
mean loneliness	0.95	0.03	3551	32.60	0.000	0.42	[0.40, 0.45]	0.95	[0.89, 1.00]	0%
loneliness over time	0.57	0.02	2065	30.94	0.000	0.26	[0.24, 0.28]	0.57	[0.53, 0.60]	0%
PPS x mean participation date	-0.04	0.01	3611	-3.47	0.001	-0.05	[-0.07, -0.02]	-0.04	[-0.06, -0.02]	100%
PPS x duration	-0.03	0.01	2514	-5.08	0.000	-0.05	[-0.07, -0.03]	-0.03	[-0.04, -0.02]	100%
PPS x mean loneliness	0.03	0.01	3488	2.69	0.007	0.03	[0.01, 0.05]	0.03	[0.01, 0.06]	100%
PPS x loneliness over time	0.02	0.01	2017	2.69	0.007	0.02	[0.01, 0.04]	0.02	[0.01, 0.04]	100%
mean loneliness x loneliness over time	0.06	0.01	2640	4.53	0.000	0.04	[0.02, 0.05]	0.06	[0.03, 0.08]	100%
mean participation date x mean loneliness	0.02	0.01	3690	1.28	0.199	0.02	[-0.01, 0.04]	0.02	[-0.01, 0.04]	100%
mean participation date x loneliness over time	0.01	0.01	2192	1.02	0.309	0.01	[-0.01, 0.02]	7.79E-03	[-0.01, 0.02]	100%
duration x mean loneliness	0.03	0.01	2568	3.99	0.000	0.04	[0.02, 0.06]	0.03	[0.01, 0.04]	100%

duration x loneliness over time	0.01	0.01	21710	0.74	0.458	0.01	[-0.01, 0.02]	5.99E-03	[-0.01, 0.02]	100%
mean participation date x duration	-0.02	0.00	2597	-4.68	0.000	-0.04	[-0.06, -0.02]	-0.02	[-0.03, -0.01]	100%
PPS x mean loneliness x loneliness over time	-0.01	0.01	2637	-1.49	0.135	-0.01	[-0.03, 0.00]	-8.39E-03	[-0.02, 0.00]	100%
mean participation date x mean loneliness x loneliness over time	-0.01	0.01	2885	-1.29	0.197	-0.01	[-0.02, 0.01]	-7.08E-03	[-0.02, 0.00]	100%
duration x mean loneliness x loneliness over time	0.00	0.01	19860	0.11	0.916	0.00	[-0.01, 0.01]	6.46E-04	[-0.01, 0.01]	100%
mean participation date x duration x mean loneliness	0.00	0.00	2496	0.97	0.330	0.01	[-0.01, 0.03]	2.73E-03	[0.00, 0.01]	100%
mean participation date x duration x loneliness over time	0.00	0.00	21340	-0.28	0.781	0.00	[-0.02, 0.01]	-1.03E-03	[-0.01, 0.01]	100%
mean participation date x duration x mean loneliness x loneliness over time	0.00	0.00	19660	-0.98	0.327	-0.01	[-0.02, 0.01]	-2.68E-03	[-0.01, 0.00]	100%

[v.] This table reports complete results of linear mixed models predicting psychological distress (measured by the Kessler-5[38]) as a function of Time, Loneliness, and Patient Probability Score (PPS), while controlling for demographic categories in 3585 participants with sufficient data. Complete information about the model is reported in main manuscript Table 2.

Table S21. Longitudinal model of distress as a function of loneliness and social isolation: Complete results.[w]

	LMER					Standardized		BRMS		
	Estimate	Std. Error	df	t value	P-value	Std. Coef.	95% CI	Median	95% CI	% in ROPE
(Intercept)	6.40	0.09	3631	75.08	0.000	0.00	[0.00, 0.00]	6.40	[6.23, 6.57]	0%
gender: male	-0.48	0.12	3431	-3.85	0.000	-0.04	[-0.07, -0.02]	-0.47	[-0.71, -0.23]	42.79%
gender: nonconforming	0.33	0.42	3635	0.79	0.430	0.01	[-0.01, 0.03]	0.34	[-0.44, 1.13]	57.67%
gender: trans	1.04	0.88	3167	1.19	0.235	0.01	[-0.01, 0.03]	0.92	[-0.68, 2.56]	23.11%
gender: other	-0.36	0.76	3540	-0.48	0.631	-0.01	[-0.03, 0.02]	-0.32	[-1.74, 1.11]	43.33%
gender: missing	0.31	0.43	3506	0.72	0.472	0.01	[-0.01, 0.03]	0.32	[-0.48, 1.14]	59.28%
education: less than advanced	-0.02	0.10	3406	-0.24	0.812	0.00	[-0.03, 0.02]	-0.02	[-0.22, 0.18]	100%
education: less than BS	0.52	0.15	3586	3.56	0.000	0.04	[0.02, 0.07]	0.51	[0.23, 0.80]	32.87%
education: less than AA	0.85	0.29	3679	2.94	0.003	0.03	[0.01, 0.06]	0.83	[0.26, 1.37]	9.88%
education: missing	-0.42	0.86	3517	-0.49	0.625	-0.01	[-0.03, 0.02]	-0.37	[-1.91, 1.23]	38.22%
ethnicity: Latino	0.45	0.21	3480	2.12	0.034	0.03	[0.00, 0.05]	0.45	[0.03, 0.88]	49.83%
ethnicity: unknown	0.94	0.51	4014	1.85	0.064	0.02	[0.00, 0.05]	0.89	[-0.08, 1.85]	17.82%
ethnicity: missing	-0.13	0.26	3522	-0.51	0.610	-0.01	[-0.03, 0.02]	-0.14	[-0.62, 0.35]	88.55%
racial identity: AA	-0.58	0.26	3668	-2.28	0.023	-0.03	[-0.05, 0.00]	-0.58	[-1.09, -0.10]	30.23%
racial identity: AAPI	-0.44	0.27	3453	-1.66	0.097	-0.02	[-0.04, 0.00]	-0.45	[-0.96, 0.10]	50.34%
racial identity: AI	-0.27	0.48	3757	-0.57	0.572	-0.01	[-0.03, 0.02]	-0.26	[-1.16, 0.67]	58.64%
racial identity: multiple	0.31	0.35	3359	0.88	0.381	0.01	[-0.01, 0.03]	0.32	[-0.37, 1.01]	63.78%
racial identity: unknown	0.02	0.78	3566	0.03	0.978	0.00	[-0.02, 0.02]	0.03	[-1.44, 1.51]	45.04%
racial identity: missing	0.52	0.44	3442	1.17	0.243	0.01	[-0.01, 0.04]	0.49	[-0.36, 1.36]	44.93%
setting: urban	0.02	0.10	3444	0.19	0.853	0.00	[-0.02, 0.03]	0.02	[-0.17, 0.22]	100%
setting: rural	0.14	0.14	3540	1.03	0.303	0.01	[-0.01, 0.04]	0.16	[-0.12, 0.42]	98.53%
setting: missing	-0.29	0.75	3632	-0.38	0.705	0.00	[-0.03, 0.02]	-0.25	[-1.73, 1.13]	44.23%
age	-0.03	0.00	3433	-10.58	0.000	-0.13	[-0.15, -0.10]	-0.03	[-0.04, -0.03]	100%
mean loneliness	1.00	0.03	4278	30.91	0.000	0.44	[0.42, 0.47]	1.00	[0.93, 1.06]	0%
loneliness over time	0.56	0.02	2265	26.86	0.000	0.26	[0.24, 0.28]	0.56	[0.52, 0.60]	0%
mean participation date	-0.10	0.02	4161	-3.95	0.000	-0.06	[-0.09, -0.03]	-0.10	[-0.14, -0.05]	100%
duration	-0.02	0.01	2863	-1.71	0.087	-0.02	[-0.04, 0.00]	-0.02	[-0.05, 0.00]	100%
living alone	-0.56	0.09	14340	-6.28	0.000	-0.11	[-0.15, -0.08]	-0.56	[-0.74, -0.39]	10.69%
PPS	0.78	0.03	3985	29.48	0.000	0.42	[0.39, 0.45]	0.78	[0.73, 0.83]	0%
mean loneliness x loneliness over time	0.05	0.01	2822	3.42	0.001	0.03	[0.01, 0.05]	0.05	[0.02, 0.08]	100%
mean loneliness x mean participation date	0.01	0.01	4388	0.80	0.422	0.01	[-0.02, 0.04]	0.01	[-0.02, 0.04]	100%
loneliness over time x mean participation date	0.00	0.01	2342	0.14	0.891	0.00	[-0.02, 0.02]	0.00	[-0.02, 0.02]	100%
mean loneliness x duration	0.03	0.01	2808	3.36	0.001	0.04	[0.02, 0.06]	0.03	[0.01, 0.04]	100%
loneliness over time x duration	0.01	0.01	21670	1.07	0.287	0.01	[-0.01, 0.03]	0.01	[-0.01, 0.03]	100%
mean participation date x duration	-0.02	0.01	2784	-3.84	0.000	-0.05	[-0.07, -0.02]	-0.02	[-0.04, -0.01]	100%
mean loneliness x living alone	-0.08	0.05	11840	-1.62	0.105	-0.04	[-0.08, 0.01]	-0.08	[-0.18, 0.02]	100%
loneliness over time x living alone	0.04	0.04	2545	0.82	0.414	0.01	[-0.01, 0.03]	0.04	[-0.05, 0.12]	100%
mean participation date x living alone	0.09	0.04	10520	2.13	0.033	0.05	[0.00, 0.10]	0.09	[0.01, 0.17]	100%
duration x living alone	-0.01	0.03	3481	-0.49	0.622	-0.01	[-0.03, 0.02]	-0.01	[-0.07, 0.04]	100%
mean loneliness x PPS	0.02	0.01	4206	1.66	0.098	0.02	[0.00, 0.05]	0.02	[0.00, 0.05]	100%

loneliness over time x PPS	0.02	0.01	2320	2.25	0.024	0.02	[0.00, 0.04]	0.02	[0.00, 0.04]	100%
mean participation date x PPS	-0.04	0.01	4246	-3.23	0.001	-0.05	[-0.08, -0.02]	-0.04	[-0.06, -0.02]	100%
duration x PPS	-0.02	0.01	2825	-3.89	0.000	-0.04	[-0.07, -0.02]	-0.02	[-0.04, -0.01]	100%
living alone x PPS	-0.08	0.04	13690	-1.93	0.054	-0.04	[-0.08, 0.00]	-0.08	[-0.16, 0.00]	100%
mean loneliness x loneliness over time x mean participation date	-0.01	0.01	2944	-1.01	0.312	-0.01	[-0.03, 0.01]	-0.01	[-0.02, 0.01]	100%
mean loneliness x loneliness over time x duration	0.00	0.01	19590	0.08	0.940	0.00	[-0.02, 0.02]	0.00	[-0.01, 0.01]	100%
mean loneliness x mean participation date x duration	0.01	0.00	2744	2.63	0.009	0.03	[0.01, 0.06]	0.01	[0.00, 0.02]	100%
loneliness over time x mean participation date x duration	0.00	0.00	21020	-0.09	0.925	0.00	[-0.02, 0.02]	0.00	[-0.01, 0.01]	100%
mean loneliness x loneliness over time x living alone	0.02	0.03	3114	0.59	0.555	0.01	[-0.01, 0.03]	0.02	[-0.04, 0.08]	100%
mean loneliness x mean participation date x living alone	0.01	0.02	8460	0.31	0.760	0.01	[-0.04, 0.06]	0.01	[-0.04, 0.05]	100%
loneliness over time x mean participation date x living alone	0.01	0.02	2672	0.43	0.669	0.00	[-0.02, 0.03]	0.01	[-0.03, 0.05]	100%
mean loneliness x duration x living alone	0.00	0.02	3277	0.00	0.998	0.00	[-0.02, 0.02]	0.00	[-0.03, 0.03]	100%
loneliness over time x duration x living alone	-0.01	0.02	20350	-0.50	0.620	0.00	[-0.02, 0.01]	-0.01	[-0.05, 0.03]	100%
mean participation date x duration x living alone	-0.01	0.01	3129	-0.84	0.400	-0.01	[-0.04, 0.01]	-0.01	[-0.04, 0.01]	100%
mean loneliness x loneliness over time x PPS	-0.01	0.01	2957	-2.06	0.039	-0.02	[-0.04, 0.00]	-0.01	[-0.03, 0.00]	100%
mean loneliness x mean participation date x PPS	0.00	0.01	4464	-0.01	0.990	0.00	[-0.03, 0.03]	0.00	[-0.01, 0.01]	100%
loneliness over time x mean participation date x PPS	0.00	0.00	2336	0.23	0.817	0.00	[-0.02, 0.02]	0.00	[-0.01, 0.01]	100%
mean loneliness x duration x PPS	0.00	0.00	2720	-0.50	0.618	-0.01	[-0.03, 0.02]	0.00	[-0.01, 0.01]	100%
loneliness over time x duration x PPS	0.01	0.00	21690	1.67	0.096	0.01	[0.00, 0.03]	0.01	[0.00, 0.02]	100%
mean participation date x duration x PPS	-0.01	0.00	2789	-2.29	0.022	-0.03	[-0.05, 0.00]	-0.01	[-0.01, 0.00]	100%
mean loneliness x living alone x PPS	0.05	0.02	11430	2.49	0.013	0.06	[0.01, 0.10]	0.05	[0.01, 0.10]	100%
loneliness over time x living alone x PPS	0.00	0.02	2629	0.19	0.849	0.00	[-0.02, 0.02]	0.00	[-0.03, 0.04]	100%
mean participation date x living alone x PPS	0.00	0.02	11250	0.26	0.794	0.01	[-0.04, 0.05]	0.01	[-0.03, 0.04]	100%
duration x living alone x PPS	-0.01	0.01	3307	-0.62	0.538	-0.01	[-0.03, 0.02]	-0.01	[-0.03, 0.02]	100%
mean loneliness x loneliness over time x mean participation date x duration	0.00	0.00	19140	-0.83	0.408	-0.01	[-0.03, 0.01]	0.00	[-0.01, 0.00]	100%
mean loneliness x loneliness over time x mean participation date x living alone	0.00	0.01	3367	0.20	0.840	0.00	[-0.02, 0.02]	0.00	[-0.02, 0.03]	100%
mean loneliness x loneliness over time x duration x living alone	-0.01	0.01	19040	-0.65	0.515	-0.01	[-0.02, 0.01]	-0.01	[-0.04, 0.02]	100%
mean loneliness x mean participation date x duration x living alone	-0.01	0.01	2923	-1.36	0.175	-0.02	[-0.04, 0.01]	-0.01	[-0.02, 0.00]	100%
loneliness over time x mean participation date x duration x living alone	0.00	0.01	21180	-0.07	0.949	0.00	[-0.02, 0.02]	0.00	[-0.02, 0.02]	100%
mean loneliness x loneliness over time x mean participation date x PPS	0.00	0.00	3174	1.16	0.248	0.01	[-0.01, 0.03]	0.00	[0.00, 0.01]	100%
mean loneliness x loneliness over time x duration x PPS	0.00	0.00	19650	-1.42	0.157	-0.01	[-0.03, 0.00]	0.00	[-0.01, 0.00]	100%
mean loneliness x mean participation date x duration x PPS	0.00	0.00	2728	1.13	0.259	0.01	[-0.01, 0.04]	0.00	[0.00, 0.01]	100%
loneliness over time x mean participation date x duration x PPS	0.00	0.00	21500	-0.08	0.934	0.00	[-0.02, 0.02]	0.00	[0.00, 0.00]	100%
mean loneliness x loneliness over time x living alone x PPS	0.01	0.01	3304	0.91	0.363	0.01	[-0.01, 0.03]	0.01	[-0.01, 0.04]	100%
mean loneliness x mean participation date x living alone x PPS	0.01	0.01	9977	1.07	0.285	0.03	[-0.02, 0.07]	0.01	[-0.01, 0.03]	100%
loneliness over time x mean participation date x living alone x PPS	0.00	0.01	2760	-0.03	0.973	0.00	[-0.02, 0.02]	0.00	[-0.02, 0.02]	100%

mean loneliness x duration x living alone x PPS	0.00	0.01	3062	0.51	0.614	0.01	[-0.02, 0.03]	0.00	[-0.01, 0.02]	100%
loneliness over time x duration x living alone x PPS	-0.02	0.01	20800	-1.95	0.051	-0.02	[-0.03, 0.00]	-0.02	[-0.04, 0.00]	100%
mean participation date x duration x living alone x PPS	0.01	0.01	3109	1.97	0.048	0.02	[0.00, 0.05]	0.01	[0.00, 0.02]	100%
mean loneliness x loneliness over time x mean participation date x duration x living alone	0.00	0.01	19950	0.22	0.829	0.00	[-0.02, 0.02]	0.00	[-0.01, 0.01]	100%
mean loneliness x loneliness over time x mean participation date x duration x PPS	0.00	0.00	19440	-0.05	0.959	0.00	[-0.02, 0.02]	0.00	[0.00, 0.00]	100%
mean loneliness x loneliness over time x mean participation date x living alone x PPS	0.00	0.01	3669	0.05	0.964	0.00	[-0.02, 0.02]	0.00	[-0.01, 0.01]	100%
mean loneliness x loneliness over time x duration x living alone x PPS	0.02	0.01	19050	2.63	0.009	0.02	[0.01, 0.04]	0.02	[0.00, 0.03]	100%
mean loneliness x mean participation date x duration x living alone x PPS	0.00	0.00	2914	1.64	0.101	0.02	[0.00, 0.05]	0.00	[0.00, 0.01]	100%
loneliness over time x mean participation date x duration x living alone x PPS	0.00	0.00	21690	0.02	0.985	0.00	[-0.02, 0.02]	0.00	[-0.01, 0.01]	100%
mean loneliness x loneliness over time x mean participation date x duration x living alone x PPS	0.00	0.00	20600	0.25	0.806	0.00	[-0.02, 0.02]	0.00	[-0.01, 0.01]	100%

[w.] This table reports complete results of linear mixed predicting psychological distress as a function of Social Distancing, Time, and Patient Probability Score (PPS), while controlling for demographic categories in 3584 participants with sufficient data. Social isolation was modeled as a categorical factor, with those who lived with others as the intercept; the term "Living alone" thus accounts for differences in distress as a function of living alone, relative to living with others. Complete information about the model is reported in main manuscript Extended Data Table 1.

Table S22. Longitudinal model of loneliness as function of social isolation: Complete results.[x]

	LMER					Standardized		BRMS		
	Estimate	Std. Error	df	t value	P-value	Std. Coef.	95% CI	Median	95% CI	% in ROPE
(Intercept)	5.36	0.05	3569	113.14	0.000	0.00	[0.00, 0.00]	5.36	[5.26, 5.45]	0.00
gender: male	-0.01	0.07	3548	-0.11	0.912	0.00	[-0.03, 0.03]	-0.02	[-0.15, 0.11]	1.00
gender: nonconforming	-0.01	0.24	3561	-0.03	0.975	0.00	[-0.03, 0.03]	0.00	[-0.47, 0.46]	0.57
gender: trans	0.28	0.52	3516	0.53	0.598	0.01	[-0.02, 0.04]	0.26	[-0.71, 1.24]	0.28
gender: other	0.80	0.44	3548	1.82	0.069	0.03	[0.00, 0.06]	0.77	[-0.08, 1.60]	0.08
gender: missing	0.18	0.25	3553	0.72	0.474	0.01	[-0.02, 0.04]	0.18	[-0.30, 0.68]	0.47
education: less than advanced	0.12	0.06	3543	2.00	0.045	0.03	[0.00, 0.06]	0.12	[0.01, 0.25]	0.88
education: less than BS	0.29	0.08	3557	3.49	0.000	0.06	[0.02, 0.09]	0.31	[0.16, 0.47]	0.07
education: less than AA	0.19	0.16	3561	1.17	0.243	0.02	[-0.01, 0.05]	0.20	[-0.17, 0.53]	0.50
education: missing	0.03	0.50	3553	0.07	0.946	0.00	[-0.03, 0.03]	0.04	[-0.95, 1.00]	0.33
ethnicity: Latino	-0.11	0.12	3552	-0.87	0.383	-0.01	[-0.04, 0.02]	-0.10	[-0.34, 0.14]	0.79
ethnicity: unknown	0.13	0.28	3590	0.46	0.645	0.01	[-0.02, 0.04]	0.13	[-0.43, 0.67]	0.48
ethnicity: missing	0.22	0.15	3548	1.51	0.132	0.02	[-0.01, 0.05]	0.22	[-0.07, 0.50]	0.45
racial identity: AA	0.00	0.15	3558	0.01	0.990	0.00	[-0.03, 0.03]	0.00	[-0.28, 0.30]	0.83
racial identity: AAPI	0.12	0.16	3540	0.76	0.450	0.01	[-0.02, 0.04]	0.10	[-0.22, 0.43]	0.70
racial identity: AI	0.08	0.27	3575	0.28	0.779	0.00	[-0.02, 0.03]	0.05	[-0.48, 0.55]	0.55
racial identity: multiple	0.15	0.21	3535	0.72	0.471	0.01	[-0.02, 0.04]	0.13	[-0.27, 0.52]	0.59
racial identity: unknown	-0.19	0.45	3554	-0.43	0.666	-0.01	[-0.04, 0.02]	-0.18	[-1.01, 0.69]	0.32
racial identity: missing	-0.01	0.25	3548	-0.05	0.959	0.00	[-0.03, 0.03]	-0.01	[-0.55, 0.45]	0.59
setting: urban	0.06	0.06	3552	0.99	0.323	0.02	[-0.02, 0.05]	0.07	[-0.05, 0.19]	0.98
setting: rural	0.09	0.08	3556	1.18	0.238	0.02	[-0.01, 0.05]	0.10	[-0.06, 0.27]	0.85
setting: missing	0.34	0.42	3586	0.82	0.412	0.01	[-0.02, 0.04]	0.34	[-0.45, 1.17]	0.28
age	-0.01	0.00	3549	-5.04	0.000	-0.08	[-0.11, -0.05]	-0.01	[-0.01, -0.01]	1.00
mean participation date	-0.03	0.01	3730	-2.83	0.005	-0.04	[-0.08, -0.01]	-0.03	[-0.05, -0.01]	1.00
duration	-0.02	0.01	1930	-1.96	0.051	-0.03	[-0.06, 0.00]	-0.02	[-0.03, 0.00]	1.00
living alone	0.49	0.02	255700	31.82	0.000	0.21	[0.20, 0.23]	0.49	[0.46, 0.52]	0.00
PPS	0.32	0.01	3647	24.45	0.000	0.39	[0.36, 0.42]	0.32	[0.29, 0.34]	0.00
mean participation date x duration	0.00	0.00	2065	1.18	0.238	0.02	[-0.01, 0.05]	0.00	[0.00, 0.01]	1.00
mean participation date x living alone	0.09	0.01	207300	11.31	0.000	0.11	[0.09, 0.13]	0.09	[0.07, 0.10]	1.00
duration x living alone	-0.01	0.01	62260	-0.73	0.467	-0.01	[-0.02, 0.01]	-0.01	[-0.02, 0.01]	1.00
mean participation date x PPS	0.01	0.01	3698	1.51	0.130	0.02	[-0.01, 0.05]	0.01	[0.00, 0.02]	1.00
duration x PPS	-0.01	0.00	1916	-1.86	0.063	-0.03	[-0.06, 0.00]	-0.01	[-0.01, 0.00]	1.00
living alone x PPS	0.06	0.01	250500	8.26	0.000	0.06	[0.05, 0.08]	0.06	[0.04, 0.07]	1.00
mean participation date x duration x living alone	0.00	0.00	34750	0.78	0.434	0.01	[-0.01, 0.03]	0.00	[-0.01, 0.01]	1.00
mean participation date x duration x PPS	0.00	0.00	2023	1.81	0.071	0.03	[0.00, 0.06]	0.00	[0.00, 0.01]	1.00
mean participation date x living alone x PPS	-0.01	0.00	226500	-2.14	0.032	-0.02	[-0.04, 0.00]	-0.01	[-0.01, 0.00]	1.00
duration x living alone x PPS	0.02	0.00	57450	5.54	0.000	0.04	[0.03, 0.06]	0.02	[0.01, 0.03]	1.00
mean participation date x duration x living alone x PPS	-0.01	0.00	43200	-2.91	0.004	-0.03	[-0.04, -0.01]	-0.01	[-0.01, 0.00]	1.00

[x]. This table reports complete results of linear mixed models predicting loneliness as a function of Time, Patient Probability Score (PPS), and social isolation, while controlling for demographic categories in 3588 participants with sufficient data. Complete information about the model is reported in main manuscript Extended Data Table 2.

Table S23. Psychological distress as a function of social distancing: Complete results.[y]

	LMER					Standardized		BRMS		
	Estimate	Std. Error	df	t value	P-value	Std. Coef.	95% CI	Median	95% CI	% in ROPE
(Intercept)	6.11	0.09	3485	69.88	0.000	0.00	[0.00, 0.00]	6.11	[5.94, 6.28]	0.00
gender: male	-0.04	0.13	3454	-0.27	0.790	0.00	[-0.03, 0.02]	-0.03	[-0.30, 0.23]	1.00
gender: nonconforming	0.45	0.45	3652	1.00	0.320	0.01	[-0.01, 0.04]	0.43	[-0.42, 1.32]	0.49
gender: trans	1.41	0.94	3229	1.50	0.133	0.02	[-0.01, 0.04]	1.20	[-0.48, 2.92]	0.16
gender: other	0.66	0.81	3537	0.82	0.411	0.01	[-0.01, 0.03]	0.60	[-0.88, 2.14]	0.34
gender: missing	0.53	0.45	3527	1.17	0.243	0.01	[-0.01, 0.04]	0.53	[-0.34, 1.38]	0.42
education: less than advanced	0.15	0.11	3425	1.35	0.178	0.02	[-0.01, 0.04]	0.14	[-0.08, 0.35]	1.00
education: less than BS	0.82	0.16	3600	5.30	0.000	0.07	[0.04, 0.10]	0.82	[0.52, 1.13]	0.01
education: less than AA	1.12	0.31	3684	3.65	0.000	0.05	[0.02, 0.07]	1.09	[0.50, 1.69]	0.02
education: missing	-0.17	0.92	3512	-0.19	0.850	0.00	[-0.03, 0.02]	-0.16	[-1.89, 1.51]	0.40
ethnicity: Latino	0.41	0.23	3498	1.80	0.072	0.02	[0.00, 0.05]	0.40	[-0.03, 0.84]	0.59
ethnicity: unknown	0.79	0.54	3985	1.48	0.140	0.02	[-0.01, 0.04]	0.75	[-0.28, 1.77]	0.27
ethnicity: missing	-0.10	0.27	3540	-0.36	0.717	0.00	[-0.03, 0.02]	-0.09	[-0.63, 0.44]	0.88
racial identity: AA	-0.31	0.27	3662	-1.14	0.253	-0.01	[-0.04, 0.01]	-0.30	[-0.83, 0.23]	0.71
racial identity: AAPI	-0.02	0.28	3445	-0.07	0.944	0.00	[-0.02, 0.02]	-0.02	[-0.57, 0.53]	0.89
racial identity: AI	-0.31	0.51	3783	-0.61	0.543	-0.01	[-0.03, 0.02]	-0.30	[-1.29, 0.67]	0.55
racial identity: multiple	0.45	0.37	3370	1.19	0.233	0.01	[-0.01, 0.04]	0.44	[-0.29, 1.18]	0.50
racial identity: unknown	-0.72	0.82	3543	-0.88	0.380	-0.01	[-0.04, 0.01]	-0.65	[-2.16, 0.87]	0.32
racial identity: missing	0.50	0.47	3457	1.06	0.289	0.01	[-0.01, 0.04]	0.50	[-0.43, 1.39]	0.43
setting: urban	0.03	0.11	3420	0.28	0.776	0.00	[-0.02, 0.03]	0.03	[-0.18, 0.24]	1.00
setting: rural	0.22	0.15	3563	1.53	0.127	0.02	[-0.01, 0.04]	0.23	[-0.06, 0.51]	0.93
setting: missing	0.12	0.78	3713	0.16	0.873	0.00	[-0.02, 0.03]	0.11	[-1.33, 1.56]	0.45
age	-0.04	0.00	3442	-11.85	0.000	-0.16	[-0.18, -0.13]	-0.04	[-0.05, -0.04]	1.00
mean participation date	-0.10	0.02	3600	-4.40	0.000	-0.06	[-0.09, -0.03]	-0.10	[-0.15, -0.06]	1.00
duration	-0.03	0.01	2706	-2.61	0.009	-0.03	[-0.05, -0.01]	-0.03	[-0.06, -0.01]	1.00
PPS	0.95	0.02	3498	38.30	0.000	0.52	[0.49, 0.54]	0.04	[0.00, 0.07]	1.00
social distancing over time	0.04	0.02	1978	2.10	0.036	0.02	[0.00, 0.04]	0.14	[0.06, 0.22]	1.00
mean social distancing	0.14	0.04	3578	3.53	0.000	0.05	[0.02, 0.08]	0.95	[0.90, 1.00]	0.00
social distancing stress over time	0.19	0.01	2259	17.54	0.000	0.14	[0.12, 0.15]	0.19	[0.17, 0.21]	1.00
mean social distancing stress	0.50	0.03	3585	19.06	0.000	0.30	[0.27, 0.33]	0.50	[0.44, 0.55]	0.04
time with others over time	0.07	0.01	1910	6.45	0.000	0.05	[0.04, 0.07]	0.07	[0.05, 0.09]	1.00
mean time with others	0.04	0.03	3537	1.12	0.263	0.02	[-0.01, 0.05]	0.04	[-0.03, 0.11]	1.00
mean participation date x duration	-0.03	0.01	2610	-5.10	0.000	-0.05	[-0.08, -0.03]	-0.03	[-0.04, -0.02]	1.00
mean participation date x PPS	-0.03	0.01	3607	-2.88	0.004	-0.04	[-0.07, -0.01]	0.00	[-0.01, 0.02]	1.00
duration x PPS	-0.02	0.01	2700	-4.41	0.000	-0.04	[-0.06, -0.02]	0.00	[-0.02, 0.02]	1.00
social distancing over time x mean social distancing	0.01	0.01	1071	1.09	0.277	0.01	[-0.01, 0.03]	0.00	[-0.04, 0.03]	1.00
social distancing stress over time x mean social distancing stress	0.04	0.01	2670	6.94	0.000	0.05	[0.04, 0.07]	0.04	[0.02, 0.06]	1.00
time with others over time x mean time with others	0.01	0.01	1762	1.81	0.070	0.01	[0.00, 0.03]	0.01	[-0.01, 0.03]	1.00
mean participation date x social distancing over time	0.00	0.01	1895	0.51	0.610	0.00	[-0.01, 0.02]	-0.03	[-0.05, -0.01]	1.00

mean participation date x mean social distancing	0.00	0.02	3656	-0.24	0.807	0.00	[-0.03, 0.02]	-0.02	[-0.04, -0.01]	1.00
mean participation date x social distancing stress over time	0.00	0.00	2332	-0.11	0.915	0.00	[-0.02, 0.01]	0.00	[-0.02, 0.02]	1.00
mean participation date x mean social distancing stress	0.01	0.01	3786	1.12	0.262	0.02	[-0.01, 0.05]	0.05	[0.02, 0.08]	1.00
mean participation date x time with others over time	0.00	0.01	2269	0.04	0.970	0.00	[-0.02, 0.02]	0.00	[-0.01, 0.01]	1.00
mean participation date x mean time with others	0.01	0.01	3754	0.57	0.567	0.01	[-0.02, 0.04]	-0.02	[-0.03, -0.01]	1.00
duration x social distancing over time	0.00	0.01	11260	0.40	0.692	0.00	[-0.01, 0.02]	0.01	[-0.01, 0.03]	1.00
duration x mean social distancing	0.04	0.01	2879	4.21	0.000	0.04	[0.02, 0.06]	0.01	[0.00, 0.02]	1.00
duration x social distancing stress over time	-0.02	0.01	19170	-2.98	0.003	-0.02	[-0.04, -0.01]	0.04	[0.03, 0.05]	1.00
duration x mean social distancing stress	0.01	0.01	2679	1.47	0.141	0.02	[-0.01, 0.04]	0.00	[-0.01, 0.01]	1.00
duration x time with others over time	0.01	0.01	9811	2.26	0.024	0.02	[0.00, 0.04]	0.01	[-0.01, 0.03]	1.00
duration x mean time with others	-0.01	0.01	2721	-0.81	0.418	-0.01	[-0.03, 0.01]	0.00	[-0.01, 0.01]	1.00
PPS x social distancing over time	0.00	0.01	2008	0.13	0.898	0.00	[-0.02, 0.02]	0.01	[0.00, 0.03]	1.00
PPS x mean social distancing	0.05	0.02	3517	3.03	0.002	0.04	[0.01, 0.07]	0.01	[-0.02, 0.04]	1.00
PPS x social distancing stress over time	0.00	0.01	2267	-0.51	0.609	0.00	[-0.02, 0.01]	-0.01	[-0.02, 0.01]	1.00
PPS x mean social distancing stress	0.01	0.01	3526	1.01	0.311	0.02	[-0.01, 0.04]	0.01	[0.00, 0.02]	1.00
PPS x time with others over time	0.01	0.00	1902	2.41	0.016	0.02	[0.00, 0.04]	0.01	[0.00, 0.02]	1.00
PPS x mean time with others	-0.02	0.02	3493	-1.53	0.127	-0.02	[-0.05, 0.01]	-0.02	[-0.05, 0.01]	1.00
mean participation date x duration x PPS	0.00	0.00	2623	-0.40	0.691	0.00	[-0.03, 0.02]	0.00	[-0.01, 0.01]	1.00
mean participation date x social distancing over time x mean social distancing	0.00	0.01	1159	0.17	0.868	0.00	[-0.02, 0.02]	-0.01	[-0.02, 0.00]	1.00
mean participation date x social distancing stress over time x mean social distancing stress	0.00	0.00	2993	-0.60	0.549	0.00	[-0.02, 0.01]	0.00	[-0.01, 0.01]	1.00
mean participation date x time with others over time x mean time with others	0.00	0.00	1603	-0.57	0.572	0.00	[-0.02, 0.01]	0.00	[-0.01, 0.01]	1.00
duration x social distancing over time x mean social distancing	0.00	0.01	12420	0.48	0.632	0.00	[-0.01, 0.02]	0.00	[-0.01, 0.00]	1.00
duration x social distancing stress over time x mean social distancing stress	-0.01	0.00	15740	-1.64	0.100	-0.01	[-0.03, 0.00]	0.00	[0.00, 0.01]	1.00
duration x time with others over time x mean time with others	0.00	0.00	10380	-0.66	0.512	-0.01	[-0.02, 0.01]	0.00	[-0.01, 0.01]	1.00
mean participation date x duration x social distancing over time	0.00	0.00	9262	-0.50	0.620	0.00	[-0.02, 0.01]	0.00	[-0.01, 0.02]	1.00
mean participation date x duration x mean social distancing	-0.01	0.00	2701	-2.20	0.028	-0.02	[-0.04, 0.00]	0.00	[-0.01, 0.01]	1.00
mean participation date x duration x social distancing stress over time	0.00	0.00	18690	0.59	0.558	0.00	[-0.01, 0.02]	0.00	[-0.01, 0.01]	1.00
mean participation date x duration x mean social distancing stress	0.00	0.00	2620	0.54	0.588	0.01	[-0.02, 0.03]	0.00	[0.00, 0.01]	1.00
mean participation date x duration x time with others over time	0.00	0.00	12680	-0.29	0.771	0.00	[-0.02, 0.02]	0.00	[0.00, 0.01]	1.00
mean participation date x duration x mean time with others	0.00	0.00	2644	0.19	0.852	0.00	[-0.02, 0.03]	0.00	[-0.01, 0.00]	1.00
PPS x social distancing over time x mean social distancing	0.00	0.00	1126	0.25	0.802	0.00	[-0.02, 0.02]	0.00	[-0.01, 0.00]	1.00
PPS x social distancing stress over time x mean social distancing stress	0.00	0.00	2722	-0.01	0.996	0.00	[-0.02, 0.02]	0.00	[0.00, 0.00]	1.00
PPS x time with others over time x mean time with others	0.00	0.00	1619	1.58	0.115	0.01	[0.00, 0.03]	0.01	[0.00, 0.01]	1.00
mean participation date x PPS x social distancing over time	0.00	0.00	1817	1.10	0.270	0.01	[-0.01, 0.03]	0.01	[0.00, 0.02]	1.00
mean participation date x PPS x mean social distancing	0.00	0.01	3596	0.11	0.914	0.00	[-0.02, 0.03]	0.00	[-0.01, 0.00]	1.00
mean participation date x PPS x social distancing stress over time	0.00	0.00	2241	0.10	0.923	0.00	[-0.02, 0.02]	0.00	[0.00, 0.00]	1.00
mean participation date x PPS x mean social distancing stress	0.01	0.01	3778	1.59	0.111	0.03	[-0.01, 0.06]	0.00	[-0.01, 0.01]	1.00
mean participation date x PPS x time with others over time	0.00	0.00	2079	1.81	0.070	0.02	[0.00, 0.03]	0.00	[-0.01, 0.01]	1.00
mean participation date x PPS x mean time with others	0.00	0.01	3738	0.11	0.909	0.00	[-0.03, 0.03]	0.00	[-0.01, 0.00]	1.00

duration x PPS x social distancing over time	0.00	0.00	13500	-0.59	0.554	-0.01	[-0.02, 0.01]	0.00	[-0.01, 0.00]	1.00
duration x PPS x mean social distancing	0.00	0.00	2843	-0.21	0.833	0.00	[-0.02, 0.02]	0.00	[0.00, 0.01]	1.00
duration x PPS x social distancing stress over time	0.01	0.00	19580	1.97	0.049	0.02	[0.00, 0.03]	0.00	[0.00, 0.01]	1.00
duration x PPS x mean social distancing stress	0.00	0.00	2624	-0.57	0.566	-0.01	[-0.03, 0.02]	0.00	[-0.01, 0.01]	1.00
duration x PPS x time with others over time	0.00	0.00	11030	1.68	0.093	0.02	[0.00, 0.03]	0.00	[-0.01, 0.01]	1.00
duration x PPS x mean time with others	0.00	0.00	2650	0.00	0.997	0.00	[-0.02, 0.02]	0.00	[0.00, 0.01]	1.00
mean participation date x duration x social distancing over time x mean social distancing	0.00	0.00	11580	0.41	0.683	0.00	[-0.01, 0.02]	0.00	[0.00, 0.01]	1.00
mean participation date x duration x social distancing stress over time x mean social distancing stress	0.00	0.00	16170	0.34	0.731	0.00	[-0.01, 0.02]	0.00	[0.00, 0.01]	1.00
mean participation date x duration x time with others over time x mean time with others	0.00	0.00	15000	-0.01	0.989	0.00	[-0.02, 0.02]	0.00	[0.00, 0.01]	1.00
mean participation date x PPS x social distancing over time x mean social distancing	0.00	0.00	1234	1.30	0.193	0.01	[-0.01, 0.03]	0.00	[0.00, 0.01]	1.00
mean participation date x PPS x social distancing stress over time x mean social distancing stress	0.00	0.00	2682	0.04	0.967	0.00	[-0.02, 0.02]	0.00	[0.00, 0.01]	1.00
mean participation date x PPS x time with others over time x mean time with others	0.00	0.00	1597	0.92	0.357	0.01	[-0.01, 0.02]	0.00	[0.00, 0.00]	1.00
duration x PPS x social distancing over time x mean social distancing	0.00	0.00	13000	0.92	0.359	0.01	[-0.01, 0.03]	0.00	[0.00, 0.00]	1.00
duration x PPS x social distancing stress over time x mean social distancing stress	0.00	0.00	16220	1.03	0.305	0.01	[-0.01, 0.02]	0.00	[0.00, 0.01]	1.00
duration x PPS x time with others over time x mean time with others	0.00	0.00	9542	0.74	0.457	0.01	[-0.01, 0.02]	0.00	[0.00, 0.00]	1.00
mean participation date x duration x PPS x social distancing over time	0.00	0.00	8418	0.46	0.646	0.00	[-0.01, 0.02]	0.00	[0.00, 0.00]	1.00
mean participation date x duration x PPS x mean social distancing	0.00	0.00	2745	0.87	0.382	0.01	[-0.01, 0.03]	0.00	[0.00, 0.00]	1.00
mean participation date x duration x PPS x social distancing stress over time	0.00	0.00	17930	-0.57	0.570	0.00	[-0.02, 0.01]	0.00	[0.00, 0.00]	1.00
mean participation date x duration x PPS x mean social distancing stress	0.00	0.00	2624	2.19	0.029	0.03	[0.00, 0.06]	0.00	[-0.01, 0.00]	1.00
mean participation date x duration x PPS x time with others over time	0.00	0.00	11540	-1.17	0.244	-0.01	[-0.03, 0.01]	0.00	[0.00, 0.00]	1.00
mean participation date x duration x PPS x mean time with others	0.00	0.00	2666	-1.60	0.111	-0.02	[-0.05, 0.00]	0.00	[0.00, 0.00]	1.00
mean participation date x duration x PPS x social distancing over time x mean social distancing	0.00	0.00	8726	0.89	0.375	0.01	[-0.01, 0.02]	0.00	[0.00, 0.00]	1.00
mean participation date x duration x PPS x social distancing stress over time x mean social distancing stress	0.00	0.00	16030	-0.52	0.602	0.00	[-0.02, 0.01]	0.00	[0.00, 0.00]	1.00
mean participation date x duration x PPS x time with others over time x mean time with others	0.00	0.00	10770	-1.25	0.212	-0.01	[-0.03, 0.01]	0.00	[0.00, 0.00]	1.00

[y] This table reports results of linear mixed models predicting psychological distress as a function of Social Distancing, Time, and Patient Probability Score (PPS), while controlling for demographic categories in 3593 participants with sufficient data. Complete information about the model is reported in main manuscript Extended Data Table 3.

Table S24. Loneliness as a function of social distancing: Complete results.[z]

	LMER					Standardized		BRMS		
	Estimate	Std. Error	df	t value	P-value	Std. Coef.	95% CI	Median	95% CI	% in ROPE
(Intercept)	5.41	0.04	3545	122.84	0.000	0.00	[0.00, 0.00]	5.40	[5.32, 5.49]	0%
gender: male	0.27	0.07	3539	4.01	0.000	0.06	[0.03, 0.08]	0.28	[0.15, 0.42]	11.75%
gender: nonconforming	0.05	0.22	3541	0.22	0.827	0.00	[-0.02, 0.03]	0.05	[-0.40, 0.48]	62.88%
gender: trans	0.51	0.48	3499	1.07	0.286	0.01	[-0.01, 0.04]	0.50	[-0.43, 1.42]	19.62%
gender: other	0.77	0.40	3532	1.91	0.056	0.03	[0.00, 0.05]	0.76	[-0.01, 1.55]	6.69%
gender: missing	0.16	0.23	3572	0.70	0.484	0.01	[-0.02, 0.04]	0.16	[-0.29, 0.61]	51.44%
education: less than advanced	0.13	0.06	3532	2.35	0.019	0.03	[0.01, 0.06]	0.13	[0.02, 0.24]	90.28%
education: less than BS	0.29	0.08	3550	3.75	0.000	0.06	[0.03, 0.08]	0.29	[0.14, 0.46]	10.90%
education: less than AA	0.26	0.15	3550	1.71	0.088	0.02	[0.00, 0.05]	0.26	[-0.04, 0.56]	34.13%
education: missing	0.12	0.46	3547	0.27	0.790	0.00	[-0.02, 0.03]	0.12	[-0.76, 1.00]	32.02%
ethnicity: Latino	-0.16	0.11	3533	-1.43	0.152	-0.02	[-0.05, 0.01]	-0.17	[-0.40, 0.06]	61.06%
ethnicity: unknown	0.12	0.26	3566	0.47	0.641	0.01	[-0.02, 0.03]	0.13	[-0.38, 0.64]	50.02%
ethnicity: missing	0.15	0.14	3538	1.07	0.284	0.01	[-0.01, 0.04]	0.14	[-0.14, 0.41]	65.96%
racial identity: AA	0.16	0.14	3590	1.21	0.228	0.02	[-0.01, 0.04]	0.16	[-0.10, 0.43]	62.91%
racial identity: AAPI	0.41	0.14	3539	2.88	0.004	0.04	[0.01, 0.07]	0.41	[0.11, 0.69]	8.65%
racial identity: AI	0.06	0.25	3553	0.25	0.804	0.00	[-0.02, 0.03]	0.07	[-0.43, 0.55]	55.87%
racial identity: multiple	0.15	0.19	3524	0.76	0.445	0.01	[-0.02, 0.04]	0.15	[-0.23, 0.50]	58.27%
racial identity: unknown	-0.41	0.41	3536	-1.00	0.318	-0.01	[-0.04, 0.01]	-0.38	[-1.22, 0.42]	25.08%
racial identity: missing	0.00	0.23	3531	0.00	1.000	0.00	[-0.03, 0.03]	0.00	[-0.46, 0.44]	61.38%
setting: urban	0.09	0.05	3531	1.71	0.087	0.02	[0.00, 0.05]	0.10	[-0.01, 0.21]	96.10%
setting: rural	0.09	0.07	3546	1.18	0.238	0.02	[-0.01, 0.05]	0.09	[-0.06, 0.23]	93.62%
setting: missing	0.39	0.39	3735	0.99	0.322	0.01	[-0.01, 0.04]	0.39	[-0.37, 1.14]	25.26%
age	-0.01	0.00	3531	-3.13	0.002	-0.05	[-0.08, -0.02]	-0.01	[-0.01, 0.00]	100%
mean participation date	-0.02	0.01	3550	-1.52	0.129	-0.02	[-0.05, 0.01]	-0.02	[-0.04, 0.00]	100%
duration	0.00	0.01	2171	0.49	0.623	0.01	[-0.02, 0.04]	0.00	[-0.01, 0.02]	100%
PPS	0.27	0.01	3544	21.49	0.000	0.32	[0.29, 0.35]	0.05	[0.01, 0.08]	100%
social distancing over time	0.05	0.02	2183	2.84	0.005	0.05	[0.02, 0.08]	0.00	[-0.04, 0.03]	100%
mean social distancing	0.00	0.02	3544	-0.23	0.820	0.00	[-0.03, 0.03]	0.27	[0.24, 0.29]	0%
social distancing stress over time	0.09	0.01	2226	9.08	0.000	0.14	[0.11, 0.17]	0.09	[0.07, 0.11]	100%
mean social distancing stress	0.25	0.01	3549	19.80	0.000	0.34	[0.31, 0.38]	0.26	[0.23, 0.28]	0.02%
time with others over time	0.06	0.01	2162	5.86	0.000	0.10	[0.07, 0.14]	0.06	[0.04, 0.08]	100%
mean time with others	0.11	0.02	3553	6.29	0.000	0.11	[0.08, 0.15]	0.11	[0.07, 0.14]	100%
mean participation date x duration	0.00	0.00	2183	-0.45	0.652	-0.01	[-0.04, 0.02]	0.00	[-0.01, 0.01]	100%
mean participation date x PPS	0.00	0.01	3548	0.71	0.479	0.01	[-0.02, 0.04]	-0.01	[-0.02, 0.01]	100%
duration x PPS	-0.01	0.00	2167	-1.86	0.063	-0.03	[-0.06, 0.00]	0.00	[-0.01, 0.00]	100%
social distancing over time x mean social distancing	0.00	0.01	2093	0.26	0.798	0.01	[-0.04, 0.05]	0.01	[0.00, 0.03]	100%
social distancing stress over time x mean social distancing stress	0.01	0.00	2319	2.27	0.024	0.03	[0.00, 0.06]	0.01	[0.00, 0.03]	100%
time with others over time x mean time with others	0.01	0.01	2154	0.93	0.353	0.02	[-0.02, 0.05]	0.00	[-0.02, 0.03]	100%

mean participation date x social distancing over time	-0.01	0.01	2192	-0.97	0.331	-0.02	[-0.05, 0.02]	0.00	[-0.01, 0.01]	100%
mean participation date x mean social distancing	0.01	0.01	3543	1.49	0.136	0.02	[-0.01, 0.05]	-0.01	[-0.01, 0.00]	100%
mean participation date x social distancing stress over time	-0.01	0.00	2227	-1.65	0.099	-0.03	[-0.06, 0.00]	0.00	[-0.01, 0.02]	100%
mean participation date x mean social distancing stress	0.01	0.01	3561	1.89	0.059	0.03	[0.00, 0.07]	0.03	[0.01, 0.04]	100%
mean participation date x time with others over time	0.00	0.00	2180	-0.70	0.487	-0.01	[-0.05, 0.02]	-0.01	[-0.02, 0.00]	100%
mean participation date x mean time with others	0.00	0.01	3557	0.28	0.779	0.00	[-0.03, 0.04]	-0.01	[-0.02, -0.01]	100%
duration x social distancing over time	0.00	0.00	282100	-1.25	0.213	0.00	[-0.01, 0.00]	0.01	[0.00, 0.02]	100%
duration x mean social distancing	0.01	0.01	2226	2.11	0.035	0.03	[0.00, 0.06]	0.01	[0.00, 0.02]	100%
duration x social distancing stress over time	-0.01	0.00	284600	-17.15	0.000	-0.04	[-0.05, -0.04]	0.01	[0.00, 0.02]	100%
duration x mean social distancing stress	0.01	0.00	2233	1.90	0.058	0.03	[0.00, 0.07]	0.00	[-0.01, 0.01]	100%
duration x time with others over time	0.00	0.00	282000	-0.71	0.477	0.00	[-0.01, 0.00]	0.00	[-0.01, 0.01]	100%
duration x mean time with others	-0.01	0.01	2248	-1.48	0.140	-0.03	[-0.06, 0.01]	0.00	[-0.01, 0.01]	100%
PPS x social distancing over time	0.00	0.01	2169	0.52	0.606	0.01	[-0.03, 0.04]	0.00	[0.00, 0.00]	100%
PPS x mean social distancing	0.03	0.01	3535	3.10	0.002	0.04	[0.02, 0.07]	0.00	[-0.01, 0.02]	100%
PPS x social distancing stress over time	0.00	0.00	2215	0.39	0.700	0.01	[-0.02, 0.04]	-0.01	[-0.02, 0.00]	100%
PPS x mean social distancing stress	0.00	0.01	3544	0.06	0.954	0.00	[-0.03, 0.03]	0.01	[-0.01, 0.02]	100%
PPS x time with others over time	0.00	0.00	2154	-0.27	0.788	0.00	[-0.04, 0.03]	0.00	[-0.01, 0.01]	100%
PPS x mean time with others	-0.01	0.01	3548	-1.20	0.229	-0.02	[-0.06, 0.01]	-0.01	[-0.02, 0.01]	100%
mean participation date x duration x PPS	0.00	0.00	2189	-0.05	0.964	0.00	[-0.03, 0.03]	0.00	[0.00, 0.00]	100%
mean participation date x social distancing over time x mean social distancing	0.00	0.01	2148	-0.62	0.536	-0.01	[-0.05, 0.03]	0.00	[-0.01, 0.00]	100%
mean participation date x social distancing stress over time x mean social distancing stress	0.00	0.00	2302	-0.12	0.908	0.00	[-0.03, 0.03]	0.00	[-0.02, 0.01]	100%
mean participation date x time with others over time x mean time with others	0.00	0.00	2194	-1.21	0.226	-0.02	[-0.06, 0.01]	0.00	[0.00, 0.00]	100%
duration x social distancing over time x mean social distancing	0.00	0.00	284300	-2.79	0.005	-0.01	[-0.02, 0.00]	0.00	[0.00, 0.00]	100%
duration x social distancing stress over time x mean social distancing stress	0.00	0.00	283200	-6.28	0.000	-0.02	[-0.02, -0.01]	0.00	[-0.01, 0.00]	100%
duration x time with others over time x mean time with others	0.00	0.00	282900	-1.69	0.091	-0.01	[-0.01, 0.00]	0.00	[0.00, 0.00]	100%
mean participation date x duration x social distancing over time	0.00	0.00	282400	2.52	0.012	0.01	[0.00, 0.01]	0.00	[-0.01, 0.01]	100%
mean participation date x duration x social distancing	0.00	0.00	2289	-0.74	0.459	-0.01	[-0.04, 0.02]	0.00	[0.00, 0.01]	100%
mean participation date x duration x social distancing stress over time	0.00	0.00	284700	1.38	0.168	0.00	[0.00, 0.01]	0.01	[0.00, 0.02]	100%
mean participation date x duration x mean social distancing stress	0.00	0.00	2263	-1.06	0.289	-0.02	[-0.06, 0.02]	0.00	[0.00, 0.00]	100%
mean participation date x duration x time with others over time	0.00	0.00	282300	3.32	0.001	0.01	[0.00, 0.02]	0.00	[-0.01, 0.00]	100%
mean participation date x duration x mean time with others	0.00	0.00	2301	1.75	0.080	0.03	[0.00, 0.07]	0.00	[0.00, 0.00]	100%
PPS x social distancing over time x mean social distancing	0.01	0.01	2076	1.08	0.279	0.03	[-0.02, 0.08]	0.00	[0.00, 0.00]	100%
PPS x social distancing stress over time x social distancing stress	0.00	0.00	2311	-0.27	0.789	0.00	[-0.03, 0.02]	0.00	[-0.01, 0.00]	100%
PPS x time with others over time x mean time with others	0.00	0.00	2146	-1.06	0.289	-0.02	[-0.06, 0.02]	0.00	[0.00, 0.00]	100%
mean participation date x PPS x social distancing over time	0.00	0.00	2185	-0.85	0.397	-0.01	[-0.05, 0.02]	0.00	[0.00, 0.01]	100%

mean participation date x PPS x mean social distancing	0.00	0.00	3540	-0.34	0.735	0.00	[-0.03, 0.02]	0.00	[-0.01, 0.00]	100%
mean participation date x PPS x social distancing stress over time	0.00	0.00	2222	-0.87	0.386	-0.01	[-0.05, 0.02]	0.00	[0.00, 0.00]	100%
mean participation date x PPS x mean social distancing stress	0.00	0.00	3561	0.41	0.680	0.01	[-0.03, 0.04]	0.00	[0.00, 0.00]	100%
mean participation date x PPS x time with others over time	0.00	0.00	2175	0.18	0.860	0.00	[-0.03, 0.04]	0.00	[0.00, 0.01]	100%
mean participation date x PPS x mean time with others	0.00	0.00	3560	0.21	0.838	0.00	[-0.03, 0.04]	0.00	[-0.01, 0.00]	100%
duration x PPS x social distancing over time	0.00	0.00	282200	-4.32	0.000	-0.01	[-0.02, -0.01]	0.00	[0.00, 0.00]	100%
duration x PPS x mean social distancing	0.00	0.00	2195	1.16	0.245	0.02	[-0.01, 0.05]	0.00	[0.00, 0.00]	100%
duration x PPS x social distancing stress over time	0.00	0.00	284600	2.23	0.026	0.01	[0.00, 0.01]	0.00	[0.00, 0.00]	100%
duration x PPS x mean social distancing stress	0.00	0.00	2202	-0.67	0.504	-0.01	[-0.05, 0.02]	0.00	[-0.01, 0.01]	100%
duration x PPS x time with others over time	0.00	0.00	282200	-3.53	0.000	-0.01	[-0.02, -0.01]	0.00	[0.00, 0.01]	100%
duration x PPS x mean time with others	0.00	0.00	2216	0.02	0.988	0.00	[-0.03, 0.04]	0.00	[-0.01, 0.00]	100%
mean participation date x duration x social distancing over time x mean social distancing	0.00	0.00	284200	0.65	0.518	0.00	[0.00, 0.01]	0.00	[0.00, 0.00]	100%
mean participation date x duration x social distancing stress over time x mean social distancing stress	0.00	0.00	283700	1.54	0.123	0.00	[0.00, 0.01]	0.00	[0.00, 0.00]	100%
mean participation date x duration x time with others over time x mean time with others	0.00	0.00	283100	-1.59	0.112	0.00	[-0.01, 0.00]	0.00	[0.00, 0.00]	100%
mean participation date x PPS x social distancing over time x mean social distancing	0.00	0.00	2175	0.36	0.717	0.01	[-0.03, 0.05]	0.00	[0.00, 0.01]	100%
mean participation date x PPS x social distancing stress over time x mean social distancing stress	0.00	0.00	2318	-0.11	0.911	0.00	[-0.03, 0.03]	0.00	[0.00, 0.00]	100%
mean participation date x PPS x time with others over time x mean time with others	0.00	0.00	2227	-0.09	0.925	0.00	[-0.04, 0.04]	0.00	[0.00, 0.00]	100%
duration x PPS x social distancing over time x mean social distancing	0.00	0.00	284100	-0.75	0.457	0.00	[-0.01, 0.00]	0.00	[0.00, 0.00]	100%
duration x PPS x social distancing stress over time x mean social distancing stress	0.00	0.00	282700	2.28	0.023	0.01	[0.00, 0.01]	0.00	[0.00, 0.00]	100%
duration x PPS x time with others over time x mean time with others	0.00	0.00	282900	-4.08	0.000	-0.01	[-0.02, -0.01]	0.00	[0.00, 0.00]	100%
mean participation date x duration x PPS x social distancing over time	0.00	0.00	278100	1.11	0.267	0.00	[0.00, 0.01]	0.00	[0.00, 0.00]	100%
mean participation date x duration x PPS x mean social distancing	0.00	0.00	2294	-0.63	0.527	-0.01	[-0.04, 0.02]	0.00	[0.00, 0.00]	100%
mean participation date x duration x PPS x social distancing stress over time	0.00	0.00	284500	-2.40	0.016	-0.01	[-0.01, 0.00]	0.00	[0.00, 0.00]	100%
mean participation date x duration x PPS x mean social distancing stress	0.00	0.00	2259	-0.60	0.547	-0.01	[-0.05, 0.03]	0.00	[0.00, 0.00]	100%
mean participation date x duration x PPS x time with others over time	0.00	0.00	282100	1.97	0.048	0.01	[0.00, 0.01]	0.00	[0.00, 0.00]	100%
mean participation date x duration x PPS x mean time with others	0.00	0.00	2304	1.34	0.180	0.03	[-0.01, 0.06]	0.00	[0.00, 0.00]	100%
mean participation date x duration x PPS x social distancing over time x mean social distancing	0.00	0.00	281200	-0.58	0.565	0.00	[-0.01, 0.00]	0.00	[0.00, 0.00]	100%
mean participation date x duration x PPS x social distancing stress over time x mean social distancing stress	0.00	0.00	284000	-3.06	0.002	-0.01	[-0.01, 0.00]	0.00	[0.00, 0.00]	100%

mean participation date x duration x PPS x time with others over time x mean time with others	0.00	0.00	282600	-1.56	0.119	-0.01	[-0.01, 0.00]	0.00	[0.00, 0.00]	100%

z. This table reports results linear mixed models predicting loneliness as a function of Social Distancing, Time, and Patient Probability Score (PPS), while controlling for demographic categories in 3588 participants with sufficient data. Complete information about the model is reported in main manuscript Extended Data Table 4.

Table S25. Longitudinal model of loneliness as a function of regional social distancing: Complete results.[aa]

	LMER					Standardized		BRMS		
	Estimate	Std. Error	df	t value	P-value	Std. Coef.	95% CI	Median	95% CI	% in ROPE
(Intercept)	5.48	0.05	3386	107.63	0.000	0.00	[0.00, 0.00]	5.47	[5.34, 5.58]	0%
gender: male	-0.01	0.08	3381	-0.12	0.902	0.00	[-0.03, 0.03]	0.02	[-0.13, 0.17]	99.02%
gender: nonconforming	-0.03	0.26	3415	-0.13	0.897	0.00	[-0.03, 0.03]	-0.08	[-0.64, 0.38]	55.31%
gender: trans	0.26	0.53	3350	0.48	0.632	0.01	[-0.02, 0.04]	0.40	[-0.69, 1.20]	20.74%
gender: other	0.72	0.45	3373	1.61	0.108	0.02	[-0.01, 0.06]	0.61	[-0.21, 1.56]	15.56%
gender: missing	0.29	0.26	3401	1.08	0.281	0.02	[-0.01, 0.05]	0.28	[-0.18, 0.74]	35.13%
education: less than advanced	0.11	0.06	3375	1.72	0.085	0.03	[0.00, 0.06]	0.12	[-0.02, 0.23]	89.08%
education: less than BS	0.28	0.09	3386	3.19	0.001	0.05	[0.02, 0.09]	0.28	[0.13, 0.44]	16.27%
education: less than AA	0.31	0.18	3385	1.72	0.086	0.03	[0.00, 0.06]	0.33	[-0.05, 0.66]	24.52%
education: missing	0.37	0.61	3363	0.61	0.541	0.01	[-0.02, 0.04]	0.48	[-0.73, 1.45]	18.18%
ethnicity: Latino	-0.14	0.13	3375	-1.06	0.289	-0.02	[-0.05, 0.01]	-0.14	[-0.39, 0.10]	68.63%
ethnicity: unknown	0.24	0.29	3405	0.81	0.418	0.01	[-0.02, 0.04]	0.25	[-0.34, 0.79]	36.65%
ethnicity: missing	0.27	0.17	3386	1.63	0.104	0.03	[-0.01, 0.06]	0.28	[-0.01, 0.66]	33.91%
racial identity: AA	-0.01	0.15	3383	-0.08	0.937	0.00	[-0.03, 0.03]	-0.03	[-0.32, 0.25]	82.97%
racial identity: AAPI	0.17	0.16	3378	1.05	0.293	0.02	[-0.01, 0.05]	0.18	[-0.12, 0.44]	55.67%
racial identity: AI	-0.09	0.28	3397	-0.32	0.750	0.00	[-0.04, 0.03]	-0.07	[-0.62, 0.50]	48.98%
racial identity: multiple	0.18	0.22	3368	0.84	0.403	0.01	[-0.02, 0.04]	0.19	[-0.27, 0.64]	47.20%
racial identity: unknown	-0.28	0.50	3387	-0.57	0.568	-0.01	[-0.04, 0.02]	-0.22	[-1.33, 0.88]	24.32%
racial identity: missing	-0.03	0.27	3372	-0.11	0.911	0.00	[-0.03, 0.03]	-0.05	[-0.52, 0.50]	57.29%
setting: urban	0.14	0.07	3378	2.13	0.033	0.04	[0.00, 0.07]	0.16	[0.02, 0.27]	74.91%
setting: rural	0.02	0.09	3384	0.21	0.836	0.00	[-0.03, 0.04]	0.00	[-0.14, 0.18]	98.96%
setting: missing	0.66	0.46	3526	1.42	0.155	0.02	[-0.01, 0.05]	0.71	[-0.19, 1.67]	9.69%
age	-0.01	0.00	3375	-4.16	0.000	-0.07	[-0.10, -0.04]	-0.01	[-0.01, 0.00]	100%
mean participation date	-0.01	0.01	3386	-0.54	0.590	-0.01	[-0.04, 0.02]	-0.01	[-0.04, 0.02]	100%
duration	0.00	0.01	1639	-0.31	0.758	-0.01	[-0.04, 0.03]	0.00	[-0.02, 0.01]	100%
regional distancing over time	0.09	0.21	1698	0.44	0.659	0.01	[-0.03, 0.05]	0.03	[-0.43, 0.47]	62.91%
mean regional distancing	0.09	0.21	3390	0.45	0.654	0.01	[-0.03, 0.04]	0.03	[-0.35, 0.42]	66.54%
PPS	0.34	0.01	3385	24.37	0.000	0.41	[0.38, 0.45]	0.35	[0.32, 0.38]	0%
mean participation date x duration	0.00	0.00	1644	0.37	0.711	0.01	[-0.03, 0.05]	0.00	[-0.01, 0.01]	100%
mean participation date x regional distancing over time	-0.10	0.09	1618	-1.09	0.278	-0.03	[-0.07, 0.02]	-0.10	[-0.27, 0.04]	88.67%
duration x regional distancing over time	0.13	0.02	264000	5.97	0.000	0.03	[0.02, 0.04]	0.13	[0.08, 0.17]	100%
mean participation date x mean regional distancing	0.02	0.08	3395	0.24	0.813	0.00	[-0.03, 0.04]	0.02	[-0.15, 0.18]	98.98%
duration x mean regional distancing	0.05	0.07	1699	0.70	0.482	0.01	[-0.02, 0.05]	0.04	[-0.11, 0.19]	98.41%
regional distancing over time x mean regional distancing	0.75	1.52	1706	0.50	0.620	0.01	[-0.03, 0.05]	0.46	[-1.68, 3.03]	13.08%
mean participation date x PPS	0.01	0.01	3382	1.99	0.047	0.03	[0.00, 0.07]	0.01	[0.00, 0.02]	100%
duration x PPS	0.00	0.00	1629	-0.36	0.720	-0.01	[-0.04, 0.03]	0.00	[-0.01, 0.01]	100%
regional distancing over time x PPS	-0.03	0.10	1696	-0.29	0.772	-0.01	[-0.05, 0.04]	-0.04	[-0.22, 0.17]	94.29%
mean regional distancing x PPS	0.19	0.09	3386	2.04	0.041	0.03	[0.00, 0.07]	0.19	[0.00, 0.34]	57.37%
mean participation date x duration x regional distancing over time	0.02	0.01	275200	2.18	0.029	0.01	[0.00, 0.01]	0.02	[0.00, 0.03]	100%

mean participation date x duration x mean regional distancing	-0.01	0.03	1726	-0.45	0.653	-0.01	[-0.05, 0.03]	-0.01	[-0.07, 0.05]	100%
mean participation date x regional distancing over time x mean regional distancing	0.36	0.62	1656	0.58	0.565	0.01	[-0.03, 0.06]	0.26	[-0.76, 1.40]	29.43%
duration x regional distancing over time x mean regional distancing	0.64	0.14	267600	4.63	0.000	0.02	[0.01, 0.02]	0.64	[0.36, 0.91]	0.08%
mean participation date x duration x PPS	0.00	0.00	1640	1.06	0.288	0.02	[-0.02, 0.06]	0.00	[0.00, 0.01]	100%
mean participation date x regional distancing over time x PPS	-0.06	0.04	1614	-1.35	0.179	-0.03	[-0.08, 0.01]	-0.05	[-0.15, 0.02]	100%
duration x regional distancing over time x PPS	-0.02	0.01	265800	-2.47	0.014	-0.01	[-0.02, 0.00]	-0.02	[-0.04, -0.01]	100%
mean participation date x mean regional distancing x PPS	0.02	0.04	3394	0.46	0.648	0.01	[-0.03, 0.04]	0.02	[-0.05, 0.10]	100%
duration x mean regional distancing x PPS	-0.01	0.03	1701	-0.26	0.797	0.00	[-0.04, 0.03]	-0.01	[-0.07, 0.06]	100%
regional distancing over time x mean regional distancing x PPS	-0.12	0.69	1692	-0.17	0.865	0.00	[-0.04, 0.03]	-0.21	[-1.32, 1.14]	20.38%
mean participation date x duration x regional distancing over time x mean regional distancing	0.12	0.05	274200	2.42	0.016	0.01	[0.00, 0.01]	0.12	[0.02, 0.22]	93.35%
mean participation date x duration x regional distancing over time x PPS	0.03	0.00	275300	7.14	0.000	0.03	[0.02, 0.03]	0.03	[0.02, 0.03]	100%
mean participation date x duration x mean regional distancing x PPS	-0.01	0.01	1726	-0.69	0.491	-0.01	[-0.05, 0.02]	-0.01	[-0.03, 0.02]	100%
mean participation date x regional distancing over time x mean regional distancing x PPS	0.45	0.28	1639	1.59	0.113	0.04	[-0.01, 0.09]	0.35	[-0.33, 0.91]	27.21%
duration x regional distancing over time x mean regional distancing x PPS	0.39	0.06	267700	6.46	0.000	0.02	[0.02, 0.03]	0.39	[0.27, 0.51]	0.06%
mean participation date x duration x regional distancing over time x mean regional distancing x PPS	-0.04	0.02	274400	-1.83	0.068	-0.01	[-0.01, 0.00]	-0.04	[-0.09, 0.00]	100%

[aa.] This table reports results of linear mixed models predicting loneliness as a function of Regional Distancing (based on regional cell phone mobility data[49] within US participants; higher values = less mobility / more distancing), Time, and Patient Probability Score (PPS), while controlling for demographic categories in 3415 participants with sufficient data. Complete information about the model is reported in main manuscript Extended Data Table 5.
